# Reciprocal genomic evolution in the ant–fungus agricultural symbiosis

**DOI:** 10.1038/ncomms12233

**Published:** 2016-07-20

**Authors:** Sanne Nygaard, Haofu Hu, Cai Li, Morten Schiøtt, Zhensheng Chen, Zhikai Yang, Qiaolin Xie, Chunyu Ma, Yuan Deng, Rebecca B. Dikow, Christian Rabeling, David R. Nash, William T. Wcislo, Seán G. Brady, Ted R. Schultz, Guojie Zhang, Jacobus J. Boomsma

**Affiliations:** 1 Centre for Social Evolution, Department of Biology, University of Copenhagen, Universitetsparken 15, DK-2100 Copenhagen, Denmark; 2 China National Genbank, BGI-Shenzhen, Shenzhen 518083, China; 3 Smithsonian Institute for Biodiversity Genomics, Smithsonian Institution, Washington DC 20013-7012, USA; 4 Department of Biology, University of Rochester, Rochester, New York 14627, USA; 5 Department of Entomology, National Museum of Natural History, Smithsonian Institution, Washington, DC 20013-7012, USA; 6 Smithsonian Tropical Research Institute, Balboa, Ancón 03092, Panama; 7 State Key Laboratory of Genetic Resources and Evolution, Kunming Institute of Zoology, Chinese Academy of Sciences, Kunming 650223, China

## Abstract

The attine ant–fungus agricultural symbiosis evolved over tens of millions of years, producing complex societies with industrial-scale farming analogous to that of humans. Here we document reciprocal shifts in the genomes and transcriptomes of seven fungus-farming ant species and their fungal cultivars. We show that ant subsistence farming probably originated in the early Tertiary (55–60 MYA), followed by further transitions to the farming of fully domesticated cultivars and leaf-cutting, both arising earlier than previously estimated. Evolutionary modifications in the ants include unprecedented rates of genome-wide structural rearrangement, early loss of arginine biosynthesis and positive selection on chitinase pathways. Modifications of fungal cultivars include loss of a key ligninase domain, changes in chitin synthesis and a reduction in carbohydrate-degrading enzymes as the ants gradually transitioned to functional herbivory. In contrast to human farming, increasing dependence on a single cultivar lineage appears to have been essential to the origin of industrial-scale ant agriculture.

## Abstract (web summary)

Attine ants, including the leaf-cutting ants, cultivate fungi as their sole source of food. Here, Nygaard *et al*. use whole genome and transcriptome sequences from seven ant species and their fungal cultivars to reconstruct the reciprocal genetic changes underlying the evolution of the ant-fungus mutualism.

## Graphical abstract

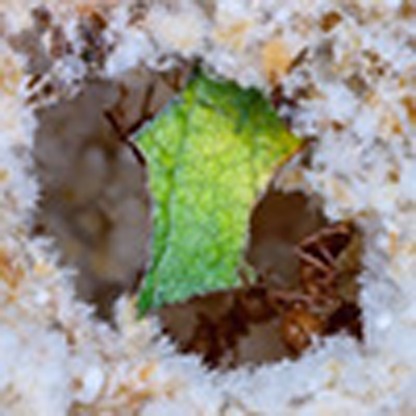

Farming created advanced human civilizations in just a few thousand years^1^, producing a huge diversity of domesticated crops with improved nutrition, growth characteristics and yield, often through polyploidization and asexual propagation^2^. Industrial-scale farming, comparable to that in humans, has evolved in only two non-human organisms, the fungus-growing ants and termites. However, the agricultural mutualisms of ants and termites were gradually modified by natural selection over time spans orders of magnitude longer than those associated with human agriculture^3^,^4^,^5^. Social insect farmers cultivate fungi in subterranean gardens to produce edible proteins, lipids and carbohydrates through decomposition rather than the photosynthesis of most human crops. The fungus-farming attine ants in particular have become model systems for mutualistic symbiosis research^6^,^7^,^8^,^9^,^10^.

Ant farming originated in South America when a hunter–gatherer ancestor irreversibly committed to cultivating fungi^3^,^5^,^11^. The fungi were not truly domesticated until many millions of years later, when one cultivar became reproductively isolated from free-living relatives and began to consistently produce specialized organs (‘gongylidia' clustered into ‘staphylae') to feed the ants^3^,^5^,^12^. The evolution of ant agriculture is thus characterized by three major transitions: (1) the obligate commitment of ancestral attine ants to farming a variety of loosely domesticated fungal cultivars; (2) the irreversible domestication of the first ‘higher attine' fungal cultivar, which fully committed it to the mutualism and severed gene flow with free-living relatives while initiating a new adaptive radiation via bilateral coevolution; and (3) the emergence of obligate functional herbivory in the ancestral leaf-cutting ant^5^. The later transitions gradually led to the abandonment of the use of dead plant material as fungal substrate and increased colony sizes by orders of magnitude. This process was completed by the evolution of polymorphic ant worker castes with complex division of labour, genetically diverse half-sibling colonies produced by multiply-inseminated founding queens and highly productive polyploid cultivars^3^,^5^,^9^,^13^,^14^,^15^.

Recent comparative research suggests that early attine farmers were metabolically less efficient than ant species with traditional diets, a deficiency that persisted until the subsequent transition to irreversible cultivar domestication^16^. Similarly, early human farmers of loosely domesticated crops had poorer health and smaller body stature compared with sympatric hunter–gatherers^17^. It seems reasonable, therefore, to expect that large-scale ant farming required a considerable accumulation of adaptive modifications, and that this process accelerated after crops became truly domesticated and no longer exchanged genes with free-living fungi. However, the molecular bases of the co-adaptations that facilitated this evolutionary process are virtually unknown and can only be elucidated using genomic data on ants and fungi representing all stages in the process that culminated in industrial-scale leaf-cutting agriculture. In the present study, we analyse five new (*Cyphomyrmex costatus*, *Trachymyrmex zeteki*, *Trachymyrmex cornetzi*, *Trachymyrmex septentrionalis* and *Atta colombica*) and two existing (*Acromyrmex echinatior* and *Atta cephalotes*) attine ant genomes, representing all genus-level branches in the neoattine crown group of the phylogenetic tree, and a more basal attine ant (*C. costatus*), along with corresponding cultivar genomes and/or transcriptomes. These data allowed us to reconstruct the structural and functional genetic changes that characterize the origin and later elaborations of fungus farming and to identify reciprocal modifications in both partners. We found that all major transitions in attine fungus farming occurred earlier than previously estimated, that attine ant evolution has involved unprecedented rates of genome rearrangements and that both ants and fungi underwent a series of coevolutionary changes in chitin-processing genes as the scale of farming increased. For the fungal cultivars, we document reductions in their capacity to decompose lignin and positive selection on chitin synthases.

## Results

### Sequencing data and phylogenetic analyses

At around 300 Mb, the five newly sequenced attine ant genomes were of fairly standard size and composition compared with published attine and other ant genomes^18^,^19^,^20^ (Supplementary Tables 1–19 and Supplementary Figs 1–7). The dikaryotic fungal symbiont of the lower attine *C. costatus* was assembled into a draft genome with a relatively large size of *ca*. 126 Mb (Supplementary Tables 5 and 7), whereas we obtained genome-wide transcriptomes for the cultivars of the higher attine ants as their increasing degrees of polykaryotic chimerism (functional polyploidy)^14^ precluded accurate genome assembly (Supplementary Table 7).

**Figure 1:**
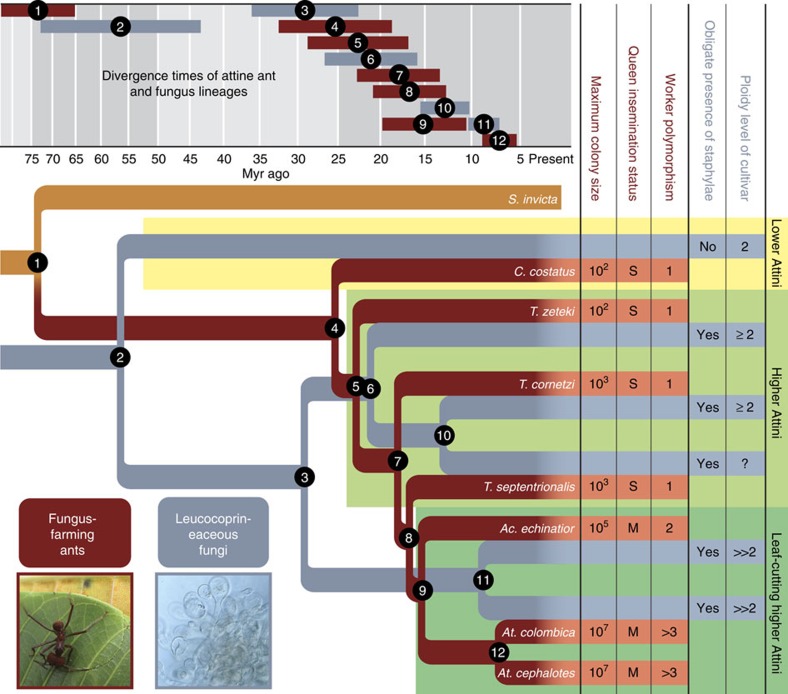
Time-calibrated phylogeny of attine ants and mutualistic fungal cultivars. The ant phylogeny (brown, attine ants in red brown) is genome-based, whereas the cultivar phylogeny (grey) is derived from transcriptomic data. *A. cephalotes* and *A. colombica* share the same fungal symbiont species *L. gongylophorus*^70^. Only the closest sequenced outgroup, the fire ant *S. invicta*, is depicted. Full phylogenies including additional ant and free-living fungal outgroups are given in Supplementary Figs 8–11. Error bars (top) indicate minimum and maximum time estimates. The character matrix (right) summarizes key morphological, behavioural and life-history traits across the three major agricultural transitions (yellow, light green and darker green background). Approximate maximum colony sizes are given as powers of 10; queen-insemination status as singly mated (S) or multiply mated (M)^13^; worker caste polymorphism as 1 (monomorphic), 2 (dimorphic: small and large workers) and>3 (polymorphic, including also a morphologically distinct soldier caste)^9^. Lower attine cultivars are simple dikaryotic mycelia (ploidy 2), whereas fully domesticated higher attine cultivars have polynucleate cells with marginal degrees of genetic chimerism (2<*n*<3) or substantial chimeric allopolyploidy (5<*n*<7) (ref. 14). See Supplementary Methods for details. Photographs: *Atta* leaf-cutting behaviour (D.R.N.) and a fungal staphyla with gongylidia (courtesy J.T. Høeg).

Phylogenies based on 2,795 and 1,075 one-to-one orthologues for the ants and their cultivars, respectively, provided four novel insights (Fig. 1 and Supplementary Figs 8–11). First, the fungal phylogeny (Fig. 1, grey branches, node 2) indicates that the leucocoprineaceous cultivar clade arose simultaneously with the farming ants *ca*. 55–60 million years ago (MYA, see also refs 5, 11), although this node has a considerable confidence range of 44–72 MYA. Second, domestication of the higher attine cultivars that produce staphylae with gongylidia occurred *ca*. 30 MYA (node 3 and 4), earlier than indicated by previous studies (*ca*. 20–25 MYA^5^,^21^). Third, the leaf-cutting ants arose *ca*. 15 MYA (node 9) rather than *ca*. 10 MYA^5^. Fourth, the single cultivar species, *Leucoagaricus gongylophorus* grown by *Atta* and *Acromyrmex* leaf-cutting ants, originated subsequent to the origin of its farmers (nodes 11 and 9, respectively) from a fungal lineage cultivated by *Trachymyrmex* ants. This finding confirms that leaf-cutting ants horizontally acquired a replacement cultivar after *Atta* and *Acromyrmex* had diverged (node 9)^21^.

### Attine ant genome evolution

**Figure 2:**
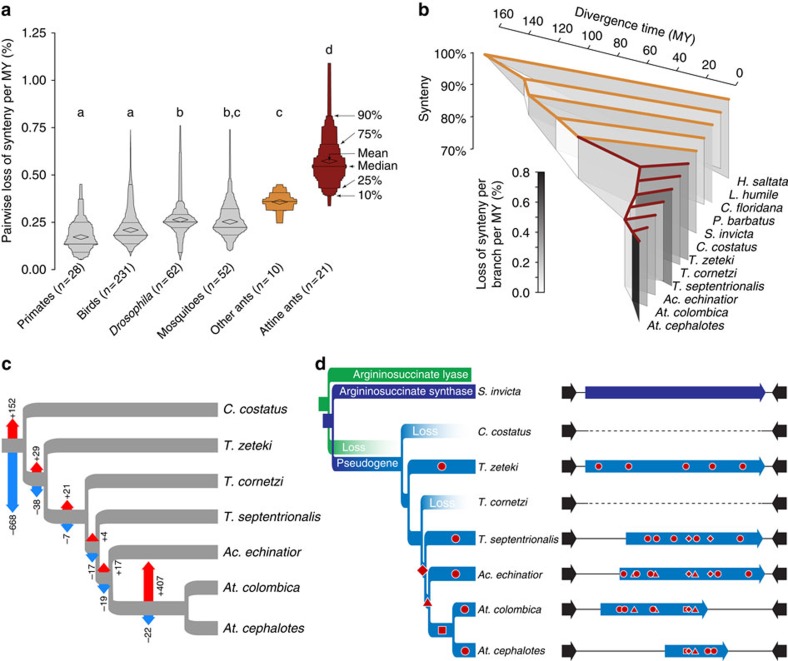
Evolutionary changes in genomic arrangement and gene family sizes in attine ants. (**a**) Attine synteny loss per MY divergence compared with other lineages with sequenced genomes. Box-percentile plots summarize the distribution of pairwise comparisons (number given after lineage name) within each clade, with means and percentiles marked. The overall difference between lineages was highly significant (*n*=6, Kruskal–Wallis *H*=101.247, *P*<0.0001). Shared letters indicate groups that did not differ significantly (Steel–Dwass test, *P*>0.05). (**b**) Synteny loss mapped onto the phylogeny of all ants with sequenced genomes, comparing each node with the earliest common ancestor (vertical axis) and with polygons shaded according to rate of loss per branch. (**c**) Numbers of expanded (red arrows) or contracted (blue arrows) gene families inferred from observed gene family sizes at terminal branches. (**d**) Functional erosion of the arginine synthesis pathway, with the argininosuccinate lyase gene (green) probably being lost in the attine ancestor and the argininosuccinate synthase gene (blue) remaining recognizable as pseudogenized residues in some genomes. Approximate lengths of pseudogenes (light blue bars) are shown relative to the *S. invicta* coding sequence of 1,239 bp. Red symbols in the branches and bars mark the origins and approximate locations of indels or in-frame stop codons: dots: lineage specific mutations; diamonds: ancestral mutations for *T. septentrionalis*, *Acromyrmex* and *Atta*; triangles: ancestral mutations for *Acromyrmex* and *Atta*; squares: ancestral mutations for *Atta*.

Attine ant genomes show very high rates of structural rearrangement. No animal lineage for which multiple genomes are available has experienced faster rates of synteny loss than the fungus-growing ants, including all non-attine ants that have been examined, which show similar levels of genome rearrangement to other insects^20^ (Fig. 2a,b; attines versus other ants, Mann–Whitney *U*_10,22_=7, *P*=0.0093; all differences with other lineages *P*<0.0001; Supplementary Fig. 12 and Supplementary Table 20). Many attine ant gene families contracted at the origin of fungus farming, suggesting that the new specialized lifestyle made some ancestral genes obsolete (Fig. 2c and Supplementary Figs 13 and 14). In contrast, the ancestral branch of the evolutionarily derived *Atta* leaf-cutting ants shows many gene family expansions, including 129 novel genes with no significant homology to known genes and no indication of horizontal gene transfer from microorganisms, consistent with previous findings^19^. These gains indicate that substantial new genetic material became available for recruitment during the recent evolution of these industrial-scale farming societies.

The arginine biosynthesis pathway is known to be absent in the evolutionarily derived leaf-cutting ants^18^,^19^, but our comparative analysis (Fig. 2d and Supplementary Figs 15 and 16) indicates that this deficiency probably originated in the earliest attine farmers with the loss of the argininosuccinate lyase gene that encodes the final enzymatic step in arginine biosynthesis. Demise of the penultimate argininosuccinate synthase gene appears to have been secondary, as pseudogenized sequence fragments can still be identified in several genomes (Fig. 2d). Arginine biosynthesis deficiency may have precluded independent life, consistent with the lack of known reversals to a hunter–gatherer lifestyle in the attine subtribe. As arginine is the most nitrogen-rich naturally occurring amino acid, it may be a suitable vector for transferring nitrogen from fungal symbionts to the farming ants. Two gene families with potential links to energy metabolism were found to be expanded in all attine ants: *Tom70* genes that encode mitochondrial import proteins and *Nardilysin*, which was previously identified as expanded in *A. echinatior*^18^ and has been linked to protein complex formation in the mitochondrial citrate cycle^22^ (Supplementary Fig. 13 and Supplementary Table 21). We also found increased *dN/dS* ratios among many energy metabolism-related genes in the higher attine ants (Supplementary Tables 22 and 23). These changes may reflect genomic responses to documented reductions in metabolic rate following the origin of fungus farming and persisting throughout the lower attine ants, only to be reversed again to normal ant levels with the irreversible domestication of staphylae-producing cultivars *ca*. 30 MY later^16^.

### Fewer carbohydrate degradation genes in domesticated crops

**Figure 3:**
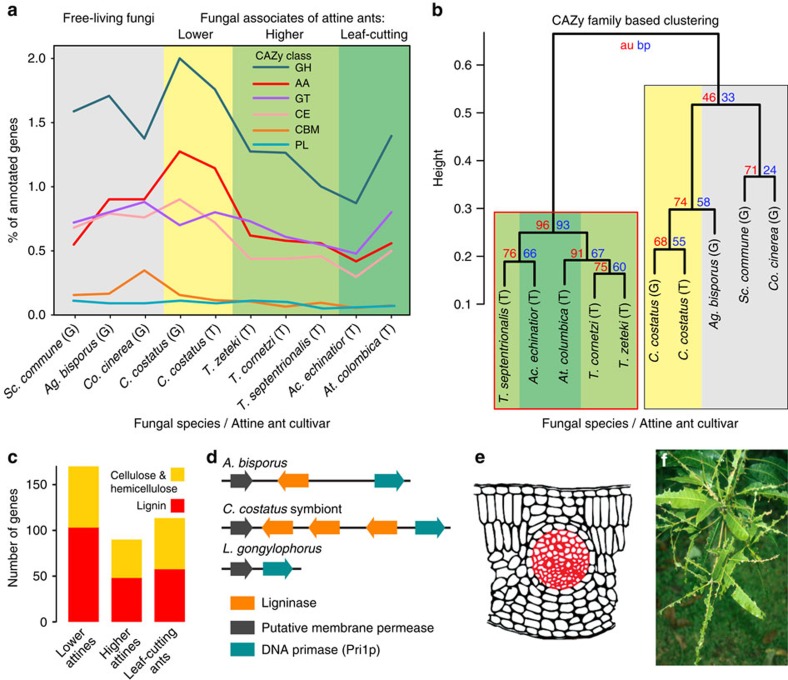
Evolutionary changes in carbohydrate-degrading potential of attine fungal cultivars. The carbohydrate-degrading potential of attine cultivars is compared with free-living outgroups. Comparisons are based on genomic (G) or transcriptomic (T) gene counts, both of which were obtained independently for *C. costat*us. (**a**) Percentage of all annotated genes in the main CAZy classes: AA, auxiliary activities; CBM, carbohydrate-binding modules; CE, carbohydrate esterases; GH, glycoside hydrolases; GT, glycosyltransferases; PL, polysaccharide lyases. Background colours as in Fig. 1; free-living fungi grey. (**b**) Hierarchical clustering based on genome-wide proportion of CAZy genes per family with approximately unbiased *P*-values (au, red), standard bootstrap percentages (bp, blue) and the significantly distinct cluster of higher attine cultivars framed in red. (**c**) Substrate-specific changes in the number of cultivar CAZyme genes for the two major plant cell wall-degrading enzyme classes (hemi) celluloses and lignins. The *C. costatus* cultivar gene numbers (‘Lower attines') are transcriptome-based to be comparable to those of the higher attine and leaf-cutting ant cultivars. (**d**) The loss of genes encoding a fungal ligninase domain in higher attine cultivars. Free-living *A. bisporus* has one copy of the ligninase gene (orange) surrounded by up- and downstream genes (grey and blue). The *C. costatus* cultivar maintains the gene order but has three tandemly arrayed copies, whereas ligninase genes have been lost in *L. gongylophoru*s. (**e**) Schematic cross-section of a leaf fragment, with the lignin-rich midrib shown in red. (**f**) Image illustrating how Panamanian *Atta* workers avoid the lignin-rich midribs when defoliating understory trees (Photo J.J.B.).

Lower attine cultivars are loosely domesticated symbionts that are likely to be capable of living apart from ants or to exchange genes with close free-living relatives^3^; thus, conditions for ant-fungus coevolution did not become unambiguously favourable until a cultivar lineage committed to genetically isolated long-term vertical transmission *ca*. 30 MYA (Fig. 1). This event coincided with the first use of fresh plant material as garden substrate^23^; thus, we compared genome-wide changes in carbohydrate-degrading potential of attine cultivars and the related free-living Agaricales fungi *Coprinopsis cinerea*, *Agaricus bisporus*, and *Schizophyllum commune*. Among these farmed and free-living fungi, the *C. costatus* cultivar has the most substantial carbohydrate-degrading repertoire (Fig. 3a and Supplementary Data 1), consistent with the recruitment of highly versatile decomposers by early farming ants once they became obligately dependent on their fungus gardens to convert dead plant material into food. However, the number of carbohydrate-degrading enzymes in truly domesticated, staphylae-producing cultivars is consistently reduced (binomial test, *P*<0.0014) across three of the six CAZy classes (encoding auxiliary activities, carbohydrate esterases and glycoside hydrolases; Fig. 3a). Clustering analysis confirmed that the *C. costatus* cultivar and the free-living fungi have similar CAZy profiles, and that fully domesticated cultivars share a distinctly different biodegradation potential (Fig. 3b and Supplementary Data 1).

The fully domesticated higher attine cultivars have significantly fewer lignin-degrading genes than the *C. costatus* cultivar (binomial test, *P*<1.6 × 10^−8^), indicating that secondary cell walls of vascular bundles, wood and bark became a marginal foraging priority after irreversible cultivar domestication (Fig. 3c). Key genes encoding proteins with the ligninase domain (IPR001621) are absent across higher attine cultivars (Supplementary Table 24), although synteny of the up- and downstream genes of the *C. costatus* cultivar and *A. bisporus* is maintained in domesticated cultivars (Fig. 3d). The *C. costatus* cultivar gene is a triple tandem repeat, possibly compensating for the absence of a second ligninase gene present in *A. bisporus* (Supplementary Table 25). The overall reduction in CAZymes and loss of lignin-degrading potential probably prevented independent saprotrophic life for the truly domesticated cultivar and is consistent with *Trachymyrmex* and *Acromyrmex* foragers primarily targeting soft leaves and petals, and *Atta* foragers avoiding the lignin-rich midribs of leaves that they otherwise harvest entirely^23^ (Fig. 3e,f). These findings match the maintenance by *Atta* colonies of large waste heaps^24^ consisting mostly of old fungus and recalcitrant cell wall material^12^,^25^,^26^.

### Crop and ant genomes coevolved to produce and digest chitin

**Figure 4:**
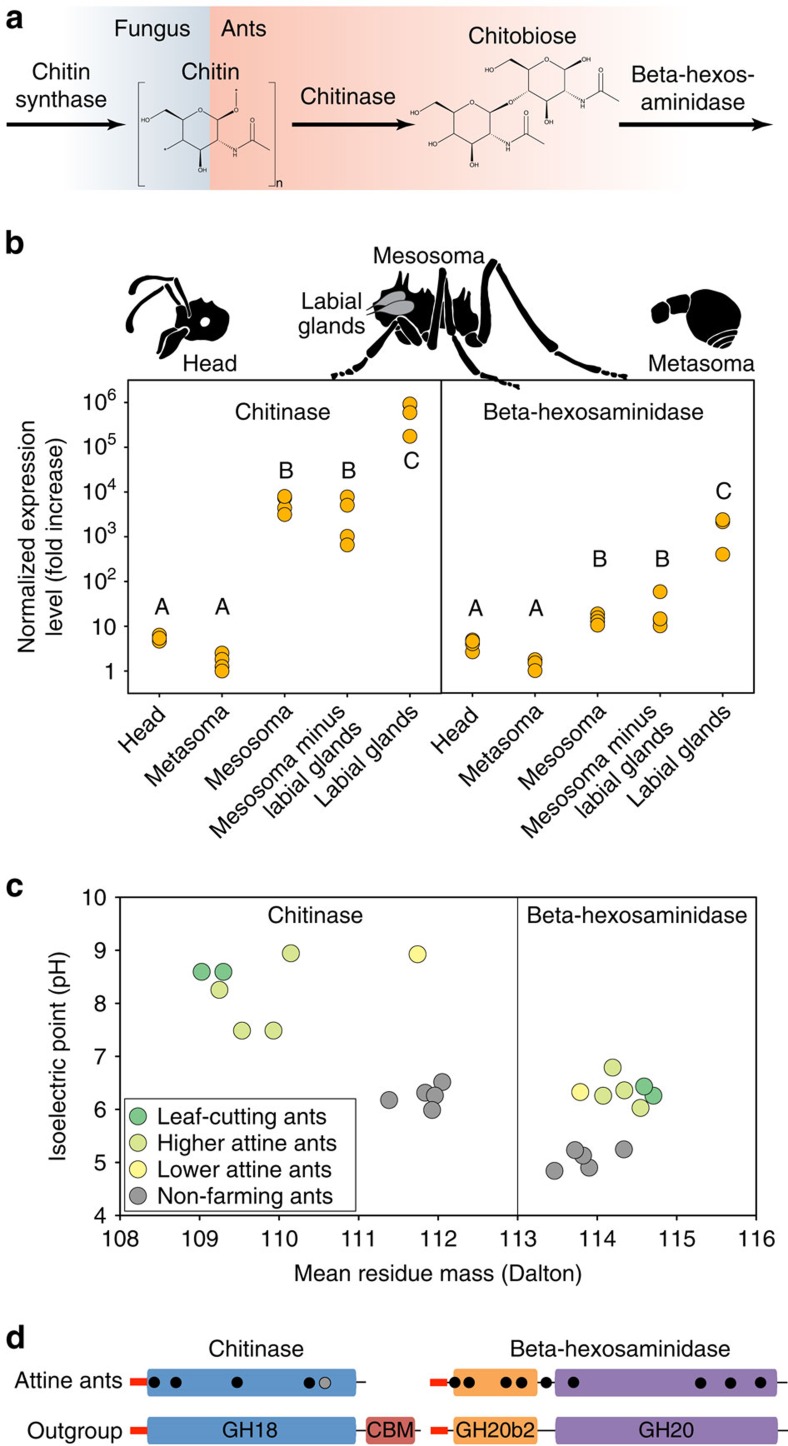
Coevolutionary changes in chitin-related functions in attine ants and their cultivars. (**a**) Two genes encoding chitinase and β-hexosaminidase enzymes show signatures of positive selection in the attine ancestor, whereas three chitin-synthase genes are positively selected in the ancestor of the higher attine cultivars. (**b**) Expression of the same genes in worker tissues showing very high expression in labial glands situated in the prothorax. Shared letters indicate that tissues do not differ significantly in gene expression level (*n*=4, Tukey's honest significant difference, *P*>0.05). High expression in the mesosoma minus labial glands might reflect expression in additional tissues with contributions from residual fragments of the labial glands. (**c**) The positively selected attine ant (*n*=7) chitinase and β-hexosaminidase proteins have significantly higher isoelectric points than the orthologous proteins in other myrmicine ants (*n*=5, phylogenetic ANOVA, both *P*<0.03). (**d**) The conserved catalytic chitinase domain (GH18) of attine ants with its lost C-terminal chitin-binding domain (CBM) and dots indicating the positions of four amino acid residues that experienced positive selection in the ancestor of all attine ants (black) or in the ancestor of the higher attines only (grey). In contrast, the full β-hexosaminidase domain architecture is intact with both GH20 and GH20b2 domains, and nine sites positively selected in the ancestor of all attine ants (black dots).

The cells of the *L. gongylophorus* cultivar of *Atta* and *Acromyrmex* leaf-cutting ants contain substantial amounts of chitin, which is degraded by chitinolytic enzymes that are produced in abundance by the ant labial glands^27^. The genomic basis for this adaptation appears to be parallel evolutionary changes in fungal pathways related to chitin synthesis and digestion of chitin by the ants. Genes encoding chitinase and β-hexosaminidase were positively selected in the ancestral attine ant (Fig. 4a; likelihood ratio test (LRT), *P*<0.05; Supplementary Tables 26–28 and Supplementary Data 2), consistent with early adaptation to fungivory. The positively selected sites (nine in the β-hexosaminidase and four in the chitinase) are mostly located on the protein surfaces (Supplementary Figs 17 and 18) and their messenger RNAs are highly expressed in the ant labial glands (Fig. 4b). The inferred isoelectric points of the proteins match earlier direct measurements^27^ and are significantly higher than those of orthologous proteins in non-farming myrmicine ants (Fig. 4c; phylogenetic analysis of variance (ANOVA), *P*<0.03; Supplementary Tables 28 and 29). These changes in charge properties probably optimize functionality in the ant foreguts, which are known to have increased pH levels^28^.

The attine ant chitinase has lost a carboxy-terminal domain often associated with binding to the peritrophic matrix of the insect gut (Fig. 4d), consistent with selection on this protein to become soluble in the labial gland fluid^27^. A single additional amino acid site was positively selected in the ancestor of the higher attines and the average chitinase residue weight in *Trachymyrmex*, *Acromyrmex* and *Atta* is reduced relative to lower attines and outgroup myrmicine ants (phylogenetic ANOVA, *P*<0.002), suggesting further co-adaptations. For the cultivars, three fungal chitin synthase genes show signs of positive selection (LRT, *P*<0.04; Supplementary Data 2), indicating modification of chitinaceous cell wall components, and the α-1,6-mannosidase domain (IPR005198) in fully domesticated attine cultivars is completely lost (Supplementary Table 24). Loss of function of this enzyme leads to enhanced chitin synthesis and cell walls of increased thickness in ascomycete fungi^29^, suggesting that the increased volumes and masses of higher attine ant gardens may be enabled by fortified cell walls^27^,^30^.

## Discussion

The results of our study shed considerable new light on the evolution of ant agriculture. First, based on the most probable divergence-date estimate, fungus farming may have originated shortly after the Yucatan impact that caused the major Cretaceous-Tertiary extinction 65 MYA and before the early Eocene climatic optimum 50–55 MYA (Fig. 1). Second, farming probably became irreversible when the ants lost the arginine biosynthesis pathway, relying instead on predictably present symbionts to supply this amino acid (Fig. 2c). Third, despite the evolution of modified chitin-processing genes that facilitated the digestion of fungal food (Fig. 4), early attine lineages remained constrained to rearing small, slow-growing gardens until a single cultivar became irreversibly domesticated *ca*. 30 MY after the origin of subsistence agriculture. Fourth, genetic isolation of this cultivar promoted coevolution with the farmers, producing not only specialized ant-feeding organs and six new genus-level lineages of ant farmers but also more massive fungus gardens and a gradual shift in decomposition profile towards active functional herbivory, ultimately resulting in the loss of an ancestral ligninase domain. As the genomes and transcriptomes that this study adds to the public domain span the main evolutionary transitions across ant fungus farming, we expect future research to clarify additional symbiotic adaptations associated with transitions from simpler to more elaborate levels of fungus farming.

Our confirmation of the secondary acquisition of the ancestor of extant *L. gongylophorus* <10 MYA ((ref. 21); this study) strongly suggests that crop innovation was critical to the establishment of industrial-scale agriculture in *Atta* and, to a lesser extent, in *Acromyrmex* (Fig. 1), even though we found relatively little evidence for substantial genome- or transcriptome-wide changes in the ancestral lineages that gave rise to the leaf-cutting ants and *L. gongylophorus*. We hypothesize that the main factor underlying the ecological dominance of the leaf-cutting ants may have been that this novel cultivar was a genetically chimeric polyploid^14^, a trait that commonly characterizes modern asexually propagated human-domesticated plants, but that is highly unusual for fungi. It thus emerges that the journeys of both ants and humans towards industrial-scale agriculture included long prior histories of subsistence farming that preceded specialization on genetically isolated crop varieties. However, although ant agriculture continued as a mutualistic symbiosis characterized by gradual reciprocal modifications and a single superior cultivar lineage with little genetic variation across the clones maintained by sympatric colonies, human agriculture proceeded by cultural evolution. Artificial selection by humans drove much faster domestication rates in a multitude of diverse cultivars^2^,^17^ accompanied by, at least so far, relatively modest reciprocal modifications of our genomes^31^.

## Methods

### Biological material and sequencing

Queenright colonies of *C. costatus*, *T. zeteki* and *T. cornetzi* were collected in Gamboa, Panama, and maintained in the lab on a diet of polenta, oatmeal and bramble leaves at 25 °C and 60–70% relative humidity (RH). For *A. colombica* and *T. septentrionalis*, ants from single colonies were collected from Gamboa, Panama and from Apalachicola National Forest, Tallahassee, FL, USA, respectively. Fungal cultures were obtained by incubation on potato dextrose yeast-extract agar plates containing streptomycin or, for *T. septentrionalis*, potato dextrose agar plates containing streptomycin+penicillin followed by propagation in liquid potato dextrose agar medium. Samples that were not immediately processed were stored in RNAlater at −80 °C. DNA and RNA was then extracted using QIAGEN kits or standard extraction protocols (see Supplementary Information for full details). Sequencing libraries with insert sizes ranging from 200 bp to 10 kbp were generated for the genomic DNA using standard procedures, whereas 200 bp fragments were used for complementary DNA sequencing libraries. All libraries were paired-end sequenced on an Illumina HiSeq 2,000 platform with read lengths of 100 bp for small insert sizes, 49 bp for large insert sizes and 90 bp for the cDNA libraries. Queen insemination data are based on earlier work^13^ supplemented for *A. cephalotes* by genotyping of *ca*. 50 workers from 6 colonies using 4 polymorphic microsatellite markers and for *T. septentrionalis* by using 4 microsatellite markers to genotype *ca*. 10 workers from 10 field colonies made available by Jon Seal, University of Texas at Tyler.

### Assembly and annotation

Genomic sequencing reads were filtered to remove low-quality reads and PCR duplicates, and then assembled using SOAPdenovo (v2.04)^32^. Contigs were first constructed based on short insert libraries and then scaffolded using paired-end information from all DNA libraries. Unresolved gap regions were locally reassembled by GapCloser (released with SOAPdenovo). Following assembly, we used BLAST^33^ against NCBI nt databases (*e*-value cutoff: 10^−5^) to remove contaminant (bacterial or bacterial+fungal) sequences.

Genomic repeats were annotated by combining several repeat detection methods as described in the Supplementary Information. Protein coding genes were annotated using GLEAN^34^, to integrate homology- and transcription-based evidence. Protein functions were inferred based on best BLASTP alignment to the SwissProt database^35^, whereas domains and Gene Ontology (GO)^36^ annotations were inferred from InterProScan 4.8 (ref. 37) against the InterPro database^38^. KEGG^39^ annotations were obtained using the KAAS server^40^.

In the absence of assembled genomes, fungal RNA-Seq reads were quality filtered and then assembled using Trinity^41^. Genes were predicted based on inferred open reading frames as described in the Supplementary Information, keeping only the longest isoform for alternatively spliced transcripts. Functional annotation was performed using the same method as described for genome-based annotations above.

### Gene family analyses

Genes from the seven attine ant and five other ant genomes (*Solenopsis invicta*, *Pogonomyrmex barbatus*, *Camponotus floridanus*, *Linepithema humile* and *Harpegnathos saltator*), as well as three outgroup insects (*Apis mellifera*, *Drosophila melanogaster* and *Nasonia vitripennis*) were clustered into gene families using OrthoMCL v2.0.9 (ref. 42). Homologous relationships among sequences were determined using BLASTp with an *e*-value cutoff of 10^−5^ and an alignment length cutoff of 50% of the gene length followed by clustering by MCL. Only gene families found in single copies in all species (2,795) were used for phylogenetic inference (see below).

One-to-one orthologous relationships among genes of attine ants and the two closest, sequenced outgroups (*S. invicta* and *P. barbatus*) were determined based on pairwise reciprocal best BLASTP (*e*-value<10^−5^) hits. Groups of orthologous genes were combined based on pairwise orthologous relationships, resulting in 7,443 one-to-one ant orthologue groups. Orthologue groups for the cultivars were similarly determined using *S. commune* and *A. bisporus* as outgroups. This resulted in 1,075 one-to-one orthologue groups, which were used to build the fungal phylogeny (see below).

Codon-based alignments of groups of one-to-one orthologous ant genes were generated with PRANK v.120716 (ref. 43) and low-scoring sites masked with Guidance v1.2 (ref. 44). Changes in *dN*/*dS* ratios were modelled with PAML^45^ version 4.7, using models with from two to four distinct *dN*/*dS* ratios. Model likelihoods were compared with log-ratio tests and false discovery rate (FDR) correction to assess significance. Alignments that showed significant increases in *dN*/*dS* ratio were then used for GO analysis using BinGO^46^ v.2.44, using the Hypergeometric test with an FDR-corrected *P*-value cutoff of 0.05 and the GO annotations of the *A. cephalotes* proteins.

Significantly expanded ant gene families were determined by using badirate^47^ to identify ‘outlier' gene families. Gene models and family assignments for these candidate outlier families were manually checked, resulting in the identification of two significantly expanded gene families: *Nardilysin and Tom70*, both of which were expanded in all attine ants. Subcellular localizations of potentially full-length *A. echinatior* and *A. colombica* Nardilysin proteins were inferred using WoLF PSORT^48^.

Overall trends in gene family expansions and contractions were assessed by counting the number of consistently expanded or contracted gene families at ancestral nodes based on gene family sizes at the terminal nodes, including novel or lost genes. Fifth and 95th sampling percentiles were calculated by permuting the data.

### Phylogenies

Protein sequences of 2,795 (ants) or 1,075 (fungi) single-copy gene families were aligned using MUSCLE^49^ with default parameters, converted into coding sequence (CDS) alignments and concatenated in Geneious v7.0 (ref. 50), resulting in a data matrix consisting of 1,886,151 amino acid sites and 13 taxa (ants) or 825,686 amino acid sites and 8 taxa (fungi). The concatenated matrix was analysed under the parsimony criterion in PAUP* v.4.0a140 (ref. 51) using a heuristic search and 100 random-taxon-addition replicates for the ants and an exhaustive search for the fungi, in each case resulting in a single optimal tree.

Using this maximum-parsimony tree as a reference tree and the 2,795 (1,075) loci as the maximum number of possible partitions, a partitioning analysis was conducted in PartitionFinder v.1.1.1 (ref. 52) in which all possible protein models were considered and compared (models=all protein) under the Bayesian Information Criterion using the hcluster search algorithm, resulting in a scheme consisting of 132 (19) partitions. These partitions and models were employed in a maximum-likelihood analysis in RAxML 7.7.7 (ref. 53), resulting in a best tree with topology identical to the maximum-parsimony topology. The partitions and models were also employed in maximum-likelihood bootstrap analyses in RAxML consisting of 1,152 pseudoreplicates under the ‘−b' (thorough search) bootstrap option, resulting once again in the same topology with bootstrap frequencies of 1.0 at all nodes.

We inferred divergence dates for the maximum-likelihood tree using the penalized likelihood approach implemented in r8s v.1.7 (ref. 54). For the ant dating analysis, the bee outgroup *A. mellifera* was excluded and two nodes in our tree were calibrated with fixed ages based on the results from a large-scale diversification analysis of the ant subfamily Myrmicinae that employed a total of 27 fossil calibrations across 251 species^11^. The two calibrated nodes in our tree correspond to (a) the most recent common ancestor (MRCA) of *C. costatus* and its sister group and (b) the MRCA of *P. barbatus* and its sister group. Three separate analyses were conducted, using the mean, 5% minimum credibility interval and 95% maximum credibility interval from Ward *et al*.^11^, respectively, to calibrate node a (26.6 (19.6, 33.8) MYA) and node b (95.4 (85.2,106.0) MYA). For the fungal dating analysis, the most distant outgroup taxon *S. commune* was used to root the tree, providing estimates for branch lengths descended from this root node and subsequently excluded from the analyses. We applied a fixed age calibration to the node corresponding to the MRCA of the outgroup *Agaricus* and its sister group using the results from a previous study^55^, a procedure similar to another diversification date analysis of lepiotaceous attine cultivars^21^. We conducted three separate analyses using different fixed ages for this node. These fixed ages were obtained from previous age estimates for this node from Geml *et al*.^55^ Thus, we conducted analyses using the mean age (73 MYA) and derived confidence ranges using the 5% minimum age (55 MYA) and the 95% maximum age (91 MYA) calibrations.

### Ant genome synteny and arginine biosynthesis pathway loss

Pairwise genome synteny was determined among attine ants, among 5 other sequenced ants, among 12 fruit flies, 8 primates, 22 birds and 16 mosquito genomes. Pairwise orthologous genes were identified based on reciprocal best BLASTp hits as described above. Syntenic blocks were then defined as containing at least five contiguous orthologous genes and were extended across gaps of no more than 4 genes. No more than 5 gene inversions in total were allowed in any pairwise syntenic block.

The loss of synteny between species pairs was assumed to follow an exponential decay process and rates of synteny loss were calculated accordingly as 1−*p*_s_^1/*T*^, where *T* is divergence time (in millions of years) and *p*_s_ the estimated proportion synteny between two species. Overall differences between taxonomic groups in their rates of pairwise synteny loss were tested using a Kruskal–Wallis non-parametric test and pairs of groups were compared using a Steel–Dwass pairwise *post*-*hoc* test. Calculations were performed in JMP version 11.2.0.

Loss of synteny along the branches of the ant phylogeny was estimated by using the FITCH package in the PHYLIP suite of programs v. 3.695 (ref. 56), which reconstructs phylogenies based on distance matrices that are assumed to be additive, but does not make assumptions about an evolutionary clock. The input file was the loss of synteny between pairs of ant species, which was treated as a distance matrix and mapped onto the ant phylogeny by using the ‘U' option to specify a user-defined tree with branch lengths derived from the dated phylogeny based on genome sequences.

Gene loss in the ant arginine biosynthesis pathway was assessed by mapping the intact argininosuccinate lyase and argininosuccinate synthase CDS sequences from *S. invicta* and *P. barbatus* to the attine genome assemblies using BLAT (v.35 × 1)^57^. Only matches to argininosuccinate synthase were found and Genewise (v2.2.0)^58^ was used to predict gene structures in the surrounding regions based on the peptide references of *S. invicta* and *P. barbatus*. Gene synteny of the flanking regions were found to be intact, whereas putative argininosuccinate synthase genes were pseudogenized by frame shifts and pre-stop codons in all cases.

### Fungal CAZy and Interpro analyses

Protein sequences of attine cultivars and three outgroup fungi (*C. cinerea* v1.0, *A. bisporus* v2.0 and *S. commune* v2.0) were matched against the CAZy database (v2013)^59^ using BLASTp, requiring full-length alignment of the query with an *e*-value<10^−6^ and identity >50%. These matches were then subjected to BLAST against a library of individual CAZy module sequences and HMMer searches^60^ using specific models for each CAZy module family, requiring both methods to yield the same family assignment. For the cultivar of *C. costatus*, CAZy counts were obtained separately for genome-wide and transcriptome-only-based annotation sets. Previously published classifications^61^,^62^ were used to categorize CAZy families according to substrate. Clustering of species based on euclidian distances between normalized counts for each CAZy family was done using the R-package pvclust version 1.3–2. Statistical significance of count differences were assessed using binomial probabilities assuming equal count distributions.

Protein Interpro (IPR)^38^ losses were initially assessed based on the annotations as described above and were verified using HMMER^60^ searches with the potentially lost IPR domain profiles against six-frame translations of all transcriptomes, as well as the genomic assemblies of the *C. costatus*, *A. echinatior* and *A. cephalotes*^25^ cultivars, with an *e*-value cutoff of 10^−2^ and requiring the length of the match to be >30% of the domain length. For the ligninase domain, we assessed the synteny of surrounding genes using manual BLAST searches against the *A. bisporus* (H97 v2.0) and *L. gongylophorus* (Ac12 v1.0) genome sequences.

### Positive selection

Positive selection was assessed using PAML (v4.6)^45^ branch-site models on the orthologue group alignments, using three different starting values for kappa and omega. We required an FDR-corrected *P*-value<0.05 from the LRT test and at least one site with a Bayes Empirical Bayes probability>0.95, and manually checked alignment quality around inferred positively selected sites. Signal peptides, protein domains and catalytic sites of positively selected proteins were analysed using PROSITE^63^ v. 20.114 and SMART^64^. Myrmicine ant orthologues of the attine chitinase and β-hexosaminidase sequences were identified using NCBI BLASTp. Protein average residue weights and isoelectric points were calculated using the pepstats programme from the EMBOSS package^65^, version 6.5.7. Significance tests were performed using phylogenetic ANOVA as implemented in the R-package phytools^66^ version 0.4–45. Protein structure modelling was done using SwissModel^67^ in both automated and alignment mode, and using several different templates for each. Although none of the models produced high scores, the overall folding remained consistent and poorly scoring regions were primarily confined to non-conserved loop regions that did not contain any of the positively selected sites.

### Expression validation

Large *A. echinatior* workers from four different colonies were submerged in liquid nitrogen and divided into head (prosoma), mesosoma (thorax and propodeum) and metasoma (gaster and petiole). Five animals were pooled per sample. Labial glands and remaining mesosoma were dissected from large workers and immediately cooled on dry ice, using pooled samples of 20 ants each. Total RNA was extracted using the QIAGEN RNeasy Mini Kit with slight modifications. RNA concentration, integrity and purity were determined using a Nanodrop spectrophotometer (Thermo Scientific) and an Experion automated electrophoresis system (Bio-Rad). Total RNA was reverse transcribed into cDNA using the iScript cDNA Synthesis Kit (Bio-Rad), after which the cDNA was diluted with water to a final concentration corresponding to 5 ng μl^−1^ of total RNA.

Gene expression levels were determined with a QX200 ddPCR system (Bio-Rad) using TaqMan probes. The two genes encoding Ribosomal Protein L18 (RPL18) and TATA-binding protein, with the Genbank accession numbers XM_011064584 and XM_011062766, respectively, were used as housekeeping genes, to normalize the expression levels across samples. Primers and probes were designed using the Primer3Plus^68^ and PCR efficiency Calculator^69^ web interfaces, and are shown in Supplementary Table 29. PCR reactions were run on a Bio-Rad S1000 Thermal Cycler using the ddPCR Supermix for Probes (Bio-Rad), ∼1 μl of template per reaction, and a final concentration of primers and probes of 0.9 and 0.25 μM, respectively. Each reaction contained primers and probes for one target gene and one housekeeping gene, so the different fluorophores of the probes allowed discrimination between the PCR products. Following PCR, the samples were transferred to the ddPCR droplet reader, to measure the number of positive and negative droplets.

Absolute transcript concentrations for each gene were obtained using the QuantaLife software and normalized through division by the geometric mean of the housekeeping gene transcript concentrations of the same samples. A pseudocount of 0.08 (corresponding to 1 positive droplet in a reaction) was added to all values before taking the base 10 logarithm to stabilize the variances. Differences in mean expression levels of each of the two target genes among the different tissues were investigated using a one-way ANOVA test followed by a *post*-*hoc* Tukey's honest significant difference test, using a significance level of 0.05 (*n*=4).

### Data availability

All sequencing data described in this study have been deposited in the relevant National Center for Biotechnology Information (NCBI) databases and accession codes are provided in Supplementary Table 2.

## Additional information

**How to cite this article:** Nygaard, S. *et al*. Reciprocal genomic evolution in the ant–fungus agricultural symbiosis. *Nat. Commun.* 7:12233 doi: 10.1038/ncomms12233 (2016).

## Supplementary Material

Supplementary Information Supplementary Figures 1 - 18, Supplementary Tables 1 - 29, Supplementary Methods and Supplementary References

Supplementary Data 1 Number of annotated fungal genes in each CAZy family

Supplementary Data 2 Gene/transcript accessions, ortholog assignments, and inferred positively selected gene families for the sequenced attine ants and fungal cultivars

## References

[b1] Diamond J. Guns, Germs and Steel: the Fates of Human Societies Norton (1997).

[b2] Meyer R. S., DuVal A. E. & Jensen H. R. Patterns and processes in crop domestication: an historical review and quantitative analysis of 203 global food crops. New Phytol. 196, 29–48 (2012). 22889076 10.1111/j.1469-8137.2012.04253.x

[b3] Mueller U. G., Gerardo N. M., Aanen D. K., Six D. L. & Schultz T. R. The evolution of agriculture in insects. Annu. Rev. Ecol. Evol. Syst. 36, 563–595 (2005).

[b4] Aanen D. K. . The evolution of fungus-growing termites and their mutualistic fungal symbionts. Proc. Natl Acad. Sci. USA 99, 14887–14892 (2002). 12386341 10.1073/pnas.222313099 PMC137514

[b5] Schultz T. R. & Brady S. G. Major evolutionary transitions in ant agriculture. Proc. Natl Acad. Sci. USA 105, 5435–5440 (2008). 18362345 10.1073/pnas.0711024105 PMC2291119

[b6] Mehdiabadi N. J. & Schultz T. R. Natural history and phylogeny of the fungus-farming ants (Hymenoptera: Formicidae: Myrmicinae: Attini). Myrmecol. News 13, 37–55 (2010).

[b7] Currie C. R., Poulsen M., Mendenhall J., Boomsma J. J. & Billen J. Coevolved crypts and exocrine glands support mutualistic bacteria in fungus-growing ants. Science 311, 81–83 (2006). 16400148 10.1126/science.1119744

[b8] Gerardo N. M., Jacobs S. R., Currie C. R. & Mueller U. G. Ancient host-pathogen associations maintained by specificity of chemotaxis and antibiosis. PLoS Biol. 4, 1358–1363 (2006). 10.1371/journal.pbio.0040235 PMC1489191 16805647

[b9] Hölldobler B. & Wilson E. O. The Leafcutter Ants: Civilization by Instinct Norton (2010).

[b10] Wirth R., Herz H., Ryel R. J., Beyschlag W. & Hölldobler B. Herbivory of Leaf-Cutting Ants. A Case Study on Atta colombica in the Tropical Rainforest of Panama Springer (2003).

[b11] Ward P. S., Brady S. G., Fisher B. L. & Schultz T. R. The evolution of myrmicine ants: phylogeny and biogeography of a hyperdiverse ant clade (Hymenoptera: Formicidae). Syst. Entomol. 40, 61–81 (2015).

[b12] Weber N. A. Gardening Ants: The Attines American Philosophical Society (1972).

[b13] Villesen P., Murakami T., Schultz T. R. & Boomsma J. J. Identifying the transition between single and multiple mating of queens in fungus-growing ants. Proc. Biol. Sci. 269, 1541–1548 (2002). 12184823 10.1098/rspb.2002.2044 PMC1691065

[b14] Kooij P. W., Aanen D. K., Schiøtt M. & Boomsma J. J. Evolutionarily advanced ant farmers rear polyploid fungal crops. J. Evol. Biol. 28, 1911–1924 (2015). 26265100 10.1111/jeb.12718 PMC5014177

[b15] Somera A. F., Lima A. M., dos Santos-Neto Á. J., Lanças F. M. & Bacci M. Leaf-cutter ant fungus gardens are biphasic mixed microbial bioreactors that convert plant biomass to polyols with biotechnological applications. Appl. Environ. Microbiol. 81, 4525–4535 (2015). 25911490 10.1128/AEM.00046-15 PMC4475879

[b16] Shik J. Z. . Metabolism and the rise of fungus cultivation by ants. Am. Nat. 184, 364–373 (2014). 25141145 10.1086/677296

[b17] Mummert A., Esche E., Robinson J. & Armelagos G. J. Stature and robusticity during the agricultural transition: evidence from the bioarchaeological record. Econ. Hum. Biol. 9, 284–301 (2011). 21507735 10.1016/j.ehb.2011.03.004

[b18] Nygaard S. . The genome of the leaf-cutting ant *Acromyrmex echinatior* suggests key adaptations to advanced social life and fungus farming. Genome Res. 21, 1339–1348 (2011). 21719571 10.1101/gr.121392.111 PMC3149500

[b19] Suen G. . The genome sequence of the leaf-cutter ant *Atta cephalotes* reveals insights into its obligate symbiotic lifestyle. PLoS Genet. 7, e1002007 (2011). 21347285 10.1371/journal.pgen.1002007 PMC3037820

[b20] Simola D. F. . Social insect genomes exhibit dramatic evolution in gene composition and regulation while preserving regulatory features linked to sociality. Genome Res. 23, 1235–1247 (2013). 23636946 10.1101/gr.155408.113 PMC3730098

[b21] Mikheyev A. S., Mueller U. G. & Abbot P. Comparative dating of attine ant and lepiotaceous cultivar phylogenies reveals coevolutionary synchrony and discord. Am. Nat. 175, E126–E133 (2010). 20415533 10.1086/652472

[b22] Chow K. M., Ma Z., Cai J., Pierce W. M. & Hersh L. B. Nardilysin facilitates complex formation between mitochondrial malate dehydrogenase and citrate synthase. Biochim. Biophys. Acta 1723, 292–301 (2005). 15809022 10.1016/j.bbagen.2005.02.010

[b23] De Fine Licht H. H. & Boomsma J. J. Forage collection, substrate preparation, and diet composition in fungus-growing ants. Ecol. Entomol. 35, 259–269 (2010).

[b24] Bot A. N. M., Currie C. R., Hart A. G. & Boomsma J. J. Waste management in leaf-cutting ants. Ethol. Ecol. Evol. 13, 225–237 (2001).

[b25] Aylward F. O. . *Leucoagaricus gongylophorus* produces diverse enzymes for the degradation of recalcitrant plant polymers in leaf-cutter ant fungus gardens. Appl. Environ. Microbiol. 79, 3770–3778 (2013). 23584789 10.1128/AEM.03833-12 PMC3675943

[b26] Grell M. N. . The fungal symbiont of *Acromyrmex* leaf-cutting ants expresses the full spectrum of genes to degrade cellulose and other plant cell wall polysaccharides. BMC Genomics 14, 928 (2013). 24373541 10.1186/1471-2164-14-928 PMC3880420

[b27] Febvay G., Decharme M. & Kermarrec A. Digestion of chitin by the labial glands of *Acromyrmex octospinosus* Reich (Hymenoptera: Formicidae). Can. J. Zool. 62, 229–234 (1984).

[b28] Erthal M., Peres Silva C. & Samuels R. I. Digestive enzymes of leaf-cutting ants, *Acromyrmex subterraneus* (Hymenoptera: Formicidae: Attini): distribution in the gut of adult workers and partial characterization. J. Insect Physiol. 50, 881–891 (2004). 15518656 10.1016/j.jinsphys.2004.06.009

[b29] Ao J., Chinnici J. L., Maddi A. & Free S. J. The N-linked outer chain mannans and the Dfg5p and Dcw1p Endo-α-1,6-Mannanases are needed for incorporation of *Candida albicans* glycoproteins into the cell wall. Eukaryot. Cell 14, 792–803 (2015). 26048011 10.1128/EC.00032-15 PMC4519743

[b30] Seal J. N. & Tschinkel W. R. Food limitation in the fungus-gardening ant *Trachymyrmex septentrionalis*. Ecol. Entomol. 33, 597–607 (2008). 10.1371/journal.pone.0158920 PMC4938500 27391485

[b31] Olalde I. . Derived immune and ancestral pigmentation alleles in a 7,000-year-old Mesolithic European. Nature 507, 225–228 (2014). 24463515 10.1038/nature12960 PMC4269527

[b32] Luo R. . SOAPdenovo2: an empirically improved memory-efficient short-read de novo assembler. Gigascience 1, 18 (2012). 23587118 10.1186/2047-217X-1-18 PMC3626529

[b33] Altschul S. F., Gish W., Miller W., Myers E. W. & Lipman D. J. Basic Local Alignment Search Tool. J. Mol. Biol. 215, 403–410 (1990). 2231712 10.1016/S0022-2836(05)80360-2

[b34] Elsik C. G. . Creating a honey bee consensus gene set. Genome Biol. 8, R13 (2007). 17241472 10.1186/gb-2007-8-1-r13 PMC1839126

[b35] Boeckmann B. The SWISS-PROT protein knowledgebase and its supplement TrEMBL in 2003. Nucleic Acids Res. 31, 365–370 (2003). 12520024 10.1093/nar/gkg095 PMC165542

[b36] Harris M. A. . The Gene Ontology (GO) database and informatics resource. Nucleic Acids Res. 32, D258–D261 (2004). 14681407 10.1093/nar/gkh036 PMC308770

[b37] Zdobnov E. M. & Apweiler R. InterProScan – an integration platform for the signature-recognition methods in InterPro. Bioinformatics 17, 847–848 (2001). 11590104 10.1093/bioinformatics/17.9.847

[b38] Mulder N. J. . InterPro: an integrated documentation resource for protein families, domains and functional sites. Brief. Bioinform. 3, 225–235 (2002). 12230031 10.1093/bib/3.3.225

[b39] Kanehisa M. & Goto S. KEGG: Kyoto Encyclopaedia of Genes and Genomes. Nucleic Acids Res. 28, 27–30 (2000). 10592173 10.1093/nar/28.1.27 PMC102409

[b40] Moriya Y., Itoh M., Okuda S., Yoshizawa A. C. & Kanehisa M. KAAS: an automatic genome annotation and pathway reconstruction server. Nucleic Acids Res. 35, W182–W185 (2007). 17526522 10.1093/nar/gkm321 PMC1933193

[b41] Grabherr M. G. . Full-length transcriptome assembly from RNA-Seq data without a reference genome. Nat. Biotechnol. 29, 644–652 (2011). 21572440 10.1038/nbt.1883 PMC3571712

[b42] Li L., Stoeckert C. J. & Roos D. S. OrthoMCL: identification of ortholog groups for eukaryotic genomes. Genome Res. 13, 2178–2189 (2003). 12952885 10.1101/gr.1224503 PMC403725

[b43] Loytynoja A. in Multiple Sequence Alignment Methods (ed. Russel D. J. 155–170Springer (2014).

[b44] Landan G. & Graur D. Local reliability measures from sets of co-optimal multiple sequence alignments. Pacific Symp. Biocomput. 13, 15–24 (2008). 18229673

[b45] Yang Z. PAML 4: phylogenetic analysis by maximum likelihood. Mol. Biol. Evol. 24, 1586–1591 (2007). 17483113 10.1093/molbev/msm088

[b46] Maere S., Heymans K. & Kuiper M. BiNGO: a Cytoscape plugin to assess overrepresentation of gene ontology categories in biological networks. Bioinformatics 21, 3448–3449 (2005). 15972284 10.1093/bioinformatics/bti551

[b47] Librado P., Vieira F. G. & Rozas J. BadiRate: estimating family turnover rates by likelihood-based methods. Bioinformatics 28, 279–281 (2012). 22080468 10.1093/bioinformatics/btr623

[b48] Horton P. . WoLF PSORT: protein localization predictor. Nucleic Acids Res. 35, W585–W587 (2007). 17517783 10.1093/nar/gkm259 PMC1933216

[b49] Edgar R. C. MUSCLE: multiple sequence alignment with high accuracy and high throughput. Nucleic Acids Res. 32, 1792–1797 (2004). 15034147 10.1093/nar/gkh340 PMC390337

[b50] Kearse M. . Geneious Basic: an integrated and extendable desktop software platform for the organization and analysis of sequence data. Bioinformatics 28, 1647–1649 (2012). 22543367 10.1093/bioinformatics/bts199 PMC3371832

[b51] Swofford D. L. PAUP*: Phylogenetic Analysis Using Parsimony (*and Other Methods) http://paup.csit.fsu.edu/ (2002).

[b52] Lanfear R., Calcott B., Ho S. Y. W. & Guindon S. PartitionFinder: combined selection of partitioning schemes and substitution models for phylogenetic analyses. Mol. Biol. Evol. 29, 1695–1701 (2012). 22319168 10.1093/molbev/mss020

[b53] Stamatakis A. RAxML version 8: a tool for phylogenetic analysis and post-analysis of large phylogenies. Bioinformatics 30, 1312–1313 (2014). 24451623 10.1093/bioinformatics/btu033 PMC3998144

[b54] Sanderson M. J. r8s: inferring absolute rates of molecular evolution and divergence times in the absence of a molecular clock. Bioinformatics 19, 301–302 (2003). 12538260 10.1093/bioinformatics/19.2.301

[b55] Geml J., Geiser D. M. & Royse D. J. Molecular evolution of *Agaricus* species based on ITS and LSU rDNA sequences. Mycol. Prog. 3, 157–176 (2004).

[b56] Felsenstein J. Phylip: phylogeny inference package (version 3.2). Cladistics 5, 164–166 (1989).

[b57] Kent W. J. BLAT – the BLAST-Like Alignment Tool. Genome Res. 12, 656–664 (2002). 11932250 10.1101/gr.229202 PMC187518

[b58] Birney E., Clamp M. & Durbin R. GeneWise and Genomewise. Genome Res. 14, 988–995 (2004). 15123596 10.1101/gr.1865504 PMC479130

[b59] Lombard V., Golaconda Ramulu H., Drula E., Coutinho P. M. & Henrissat B. The carbohydrate-active enzymes database (CAZy) in 2013. Nucleic Acids Res. 42, 490–495 (2014). 10.1093/nar/gkt1178 PMC3965031 24270786

[b60] Eddy S. R. Accelerated profile HMM searches. PLoS Comput. Biol. 7, e1002195 (2011). 22039361 10.1371/journal.pcbi.1002195 PMC3197634

[b61] Zhao Z., Liu H., Wang C. & Xu J. Correction: comparative analysis of fungal genomes reveals different plant cell wall degrading capacity in fungi. BMC Genomics 15, 6 (2014). 24422981 10.1186/1471-2164-15-6 PMC3893384

[b62] van den Brink J. & de Vries R. P. Fungal enzyme sets for plant polysaccharide degradation. Appl. Microbiol. Biotechnol. 91, 1477–1492 (2011). 21785931 10.1007/s00253-011-3473-2 PMC3160556

[b63] Sigrist C. J. A. . PROSITE, a protein domain database for functional characterization and annotation. Nucleic Acids Res. 38, D161–D166 (2010). 19858104 10.1093/nar/gkp885 PMC2808866

[b64] Letunic I., Doerks T. & Bork P. SMART: recent updates, new developments and status in 2015. Nucleic Acids Res. 43, D257–D260 (2014). 25300481 10.1093/nar/gku949 PMC4384020

[b65] Rice P., Longden I. & Bleasby A. EMBOSS: the European Molecular Biology Open Software Suite. Trends Genet. 16, 276–277 (2000). 10827456 10.1016/s0168-9525(00)02024-2

[b66] Revell L. J. phytools: an R package for phylogenetic comparative biology (and other things). Methods Ecol. Evol. 3, 217–223 (2012).

[b67] Biasini M. . SWISS-MODEL: modelling protein tertiary and quaternary structure using evolutionary information. Nucleic Acids Res. 42, W252–W258 (2014). 24782522 10.1093/nar/gku340 PMC4086089

[b68] Untergasser A. . Primer3 – new capabilities and interfaces. Nucleic Acids Res. 40, 1–12 (2012). 22730293 10.1093/nar/gks596 PMC3424584

[b69] Mallona I., Weiss J. & Marcos E.-C. pcrEfficiency: a Web tool for PCR amplification efficiency prediction. BMC Bioinformatics 12, 404 (2011). 22014212 10.1186/1471-2105-12-404 PMC3234296

[b70] De Fine Licht H. H., Boomsma J. J. & Tunlid A. Symbiotic adaptations in the fungal cultivar of leaf-cutting ants. Nat. Commun. 5, 5675 (2014). 25435021 10.1038/ncomms6675

