# Supplementary material for: Reciprocal genomic evolution in the ant–fungus agricultural symbiosis
Source: Nat Commun. 2016 Jul 20;7:12233. doi: 10.1038/ncomms12233 (PMC4961791; doi:10.1038/ncomms12233)
Supplement: Supplementary Information — Supplementary Figures 1 - 18, Supplementary Tables 1 - 29, Supplementary Methods and Supplementary References [file ncomms12233-s1.pdf]

Supplementary Figures

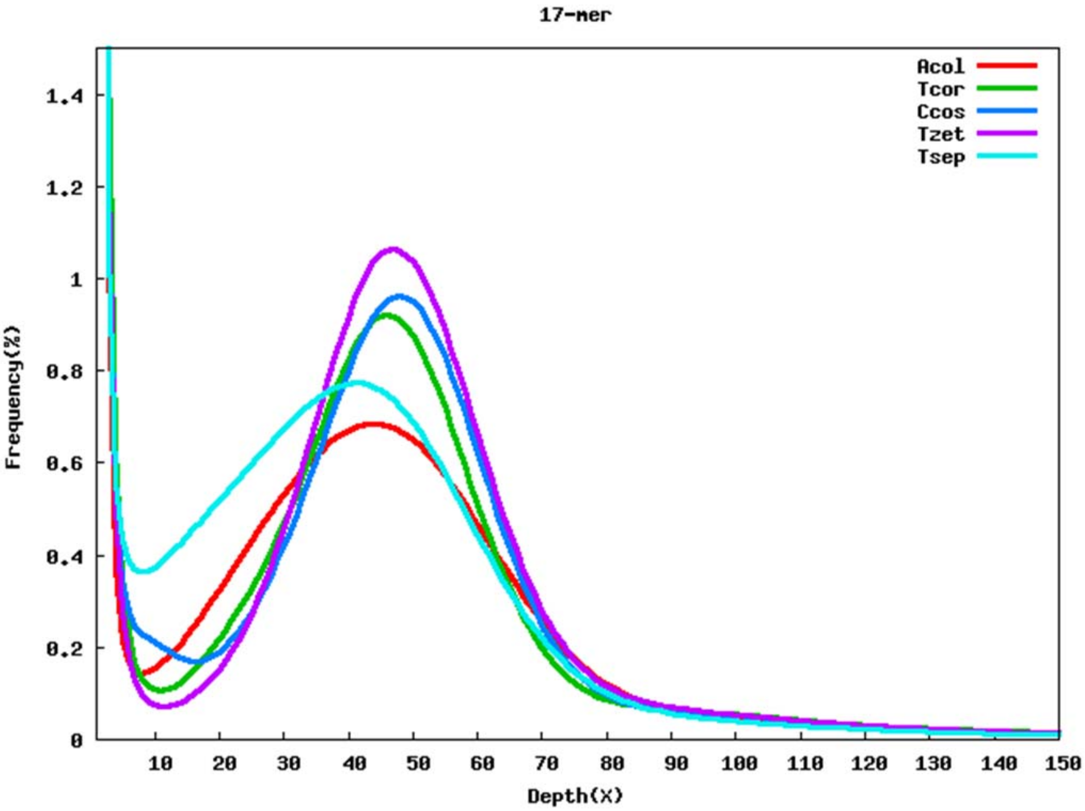

Supplementary Figure 1. K-mer distributions of ant assemblies
17-mer frequency distributions of attine ant genome assemblies, using approximately 50× coverage of reads for each assembly: *Atta colombica* (Acol), *Trachymyrmex septentrionalis* (Tsep), *T. cornetzi* (Tcor), *T. zeteki* (Tzet), and *Cyphomyrmex costatus* (Ccos).

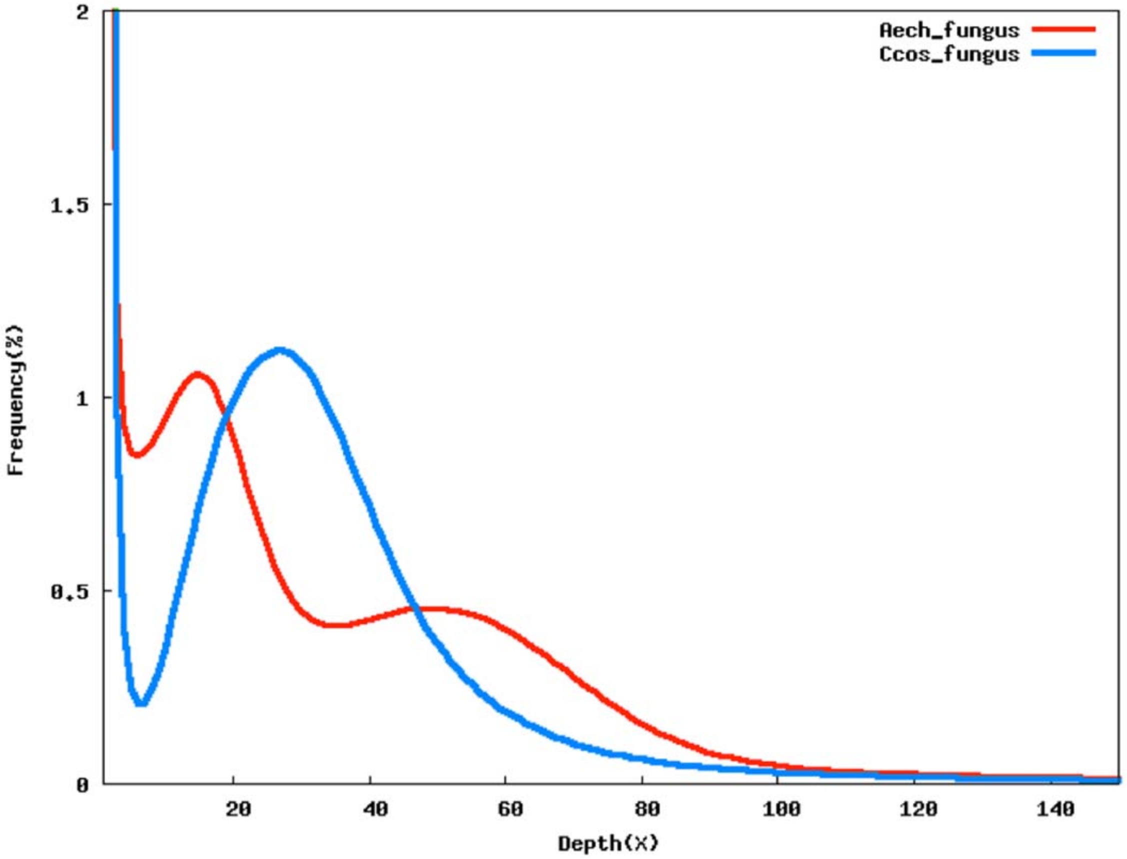

Supplementary Figure 2. K-mer distributions of cultivar assemblies

17-mer frequency distributions of fungal genome assemblies, showing a single peak for the cultivar of *C. costatus* (Ccos_fungus, blue), but two distinct peaks for the functionally allopolyploid cultivar of *Acromyrmex echinatior* (Aech_fungus, red).

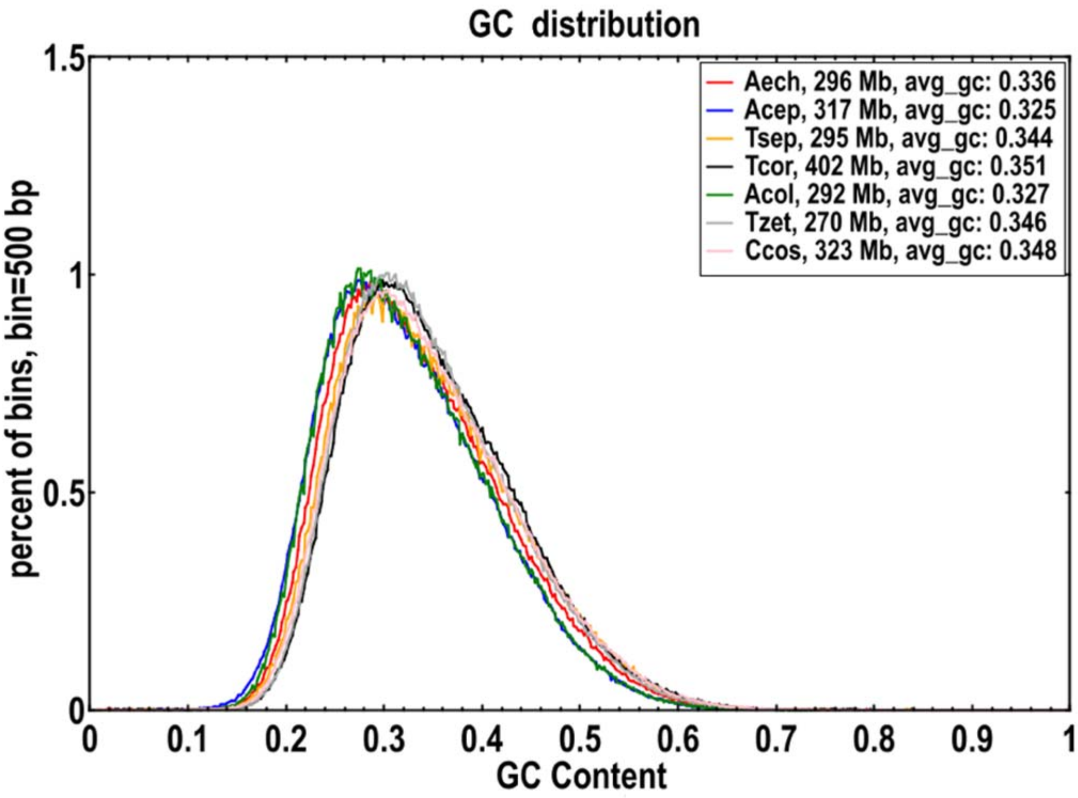

Supplementary Figure 3. GC content distributions across the attine ant genome assemblies The X-axis shows the GC content and the Y-axis the proportion of non-overlapping sliding windows of 500 bp. The legend shows species IDs (abbreviations as above, plus *Atta cephalotes* (Acep)), genome assembly sizes, and the average GC content. The ant genome assemblies have very similar GC content distributions with a peak of approximately 30% GC.

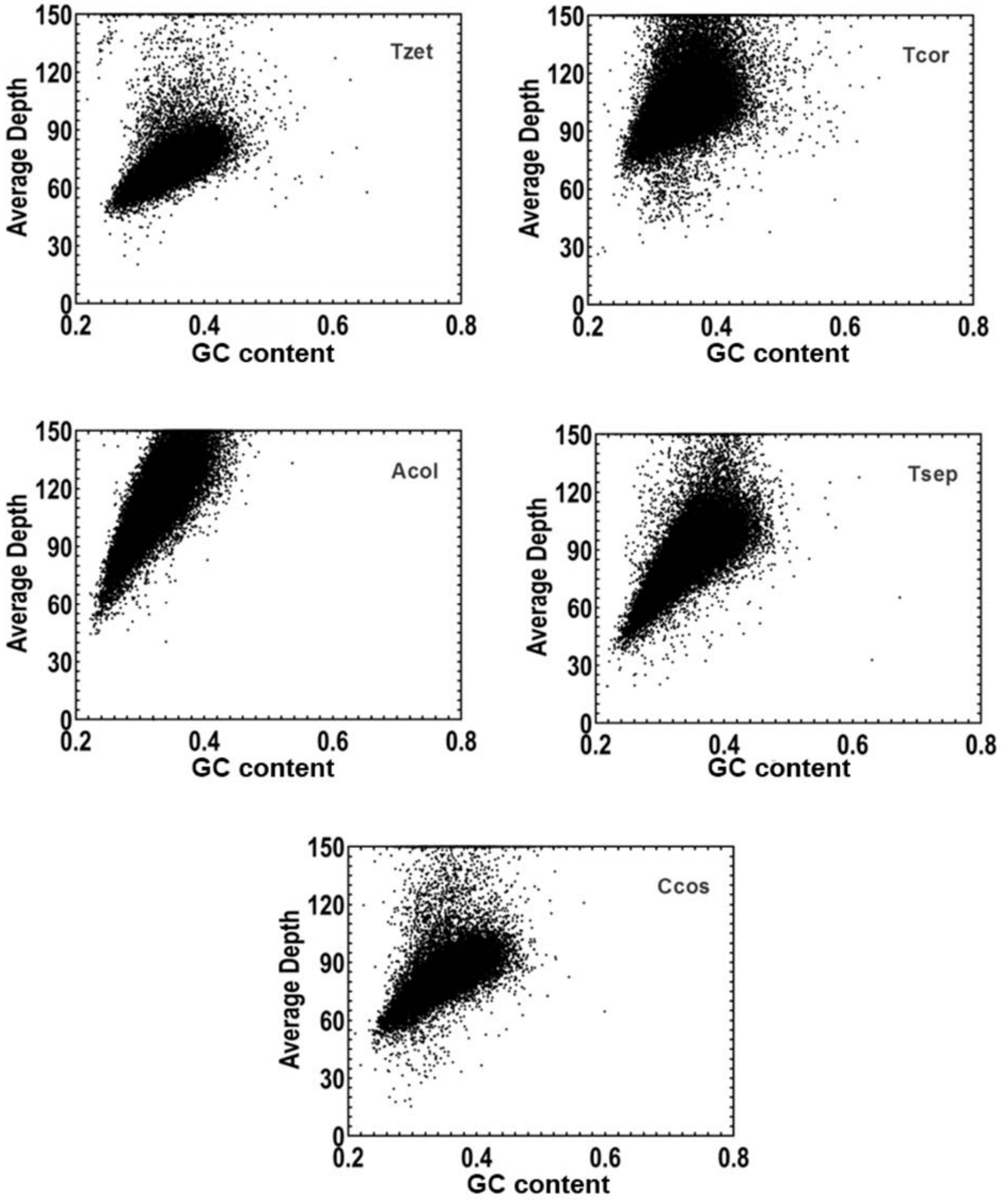

Supplementary Figure 4. Ant GC content versus sequencing depth
Correlation between GC (Guanine-Cytosine) content and sequencing depth in the five attine ant genome assemblies. The X-axis represents GC content; the Y-axis represents average sequencing depth. We used 10kb non-overlapping sliding windows and calculated GC content and average depth across these windows.

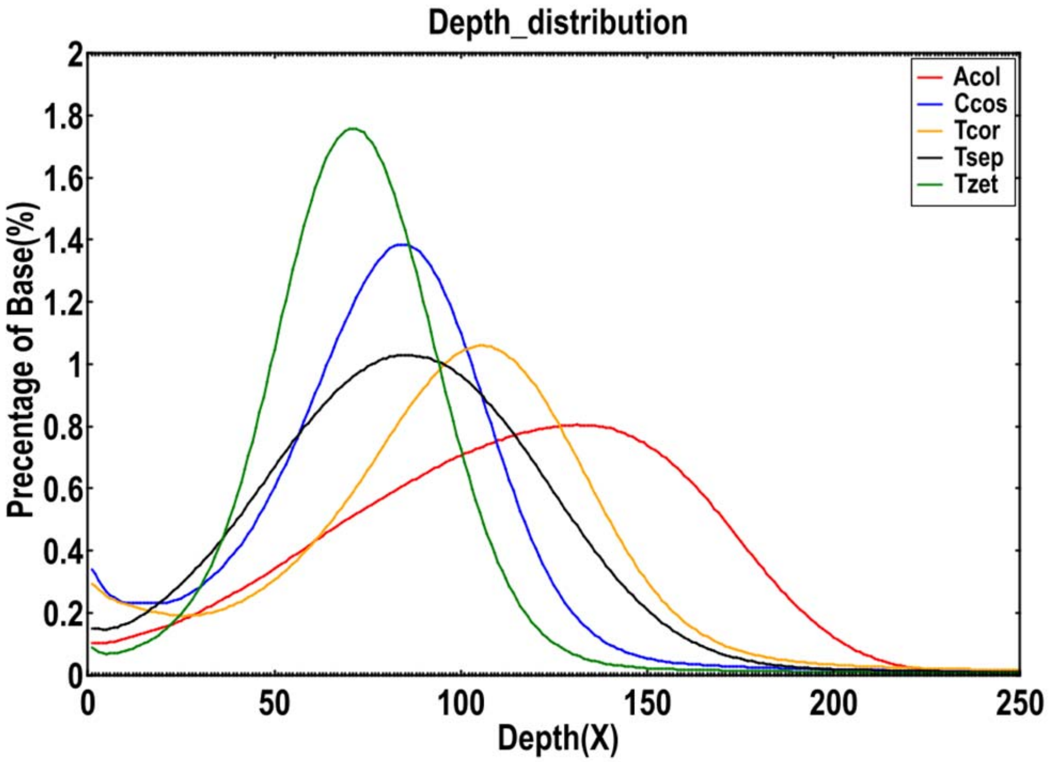

Supplementary Figure 5. Ant coverage distributions
Sequencing depth (coverage) distributions for the attine ant genome assemblies. The X-axis represents sequencing depth and the Y-axis represents the proportion of total bases at a given depth. To generate these distributions the clean reads were aligned onto the assembled genome sequence allowing for 2 mismatches for 44bp reads and 5 mismatches for the longer reads.

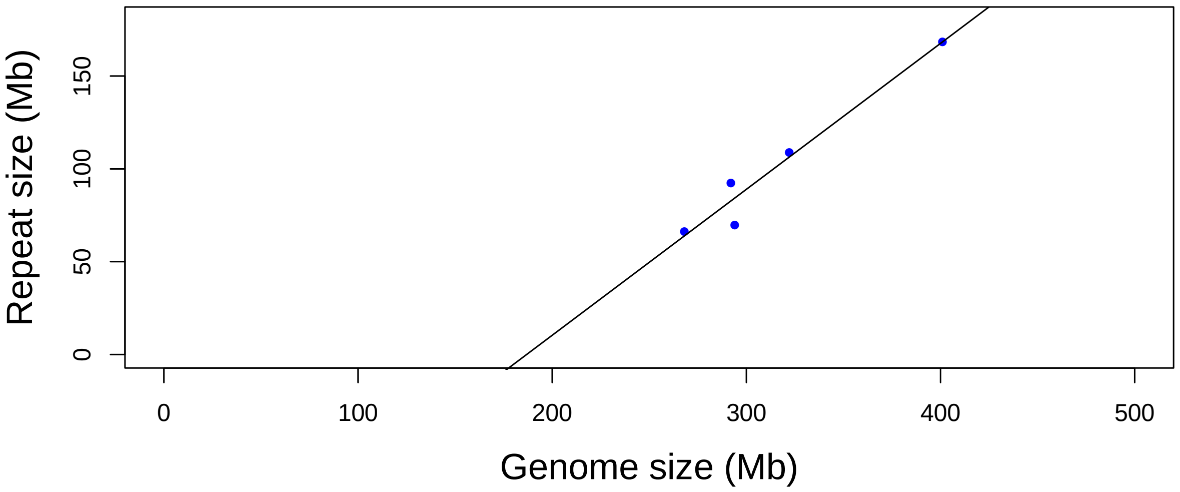

Supplementary Figure 6. Ant repeat versus assembly size

Total assembly size (X-axis) versus total repeat content (Y-axis) in the five ant genome assemblies. These numbers are linearly correlated (Pearson R = 0.976, P<0.005), with an intercept just below 200 Mb.

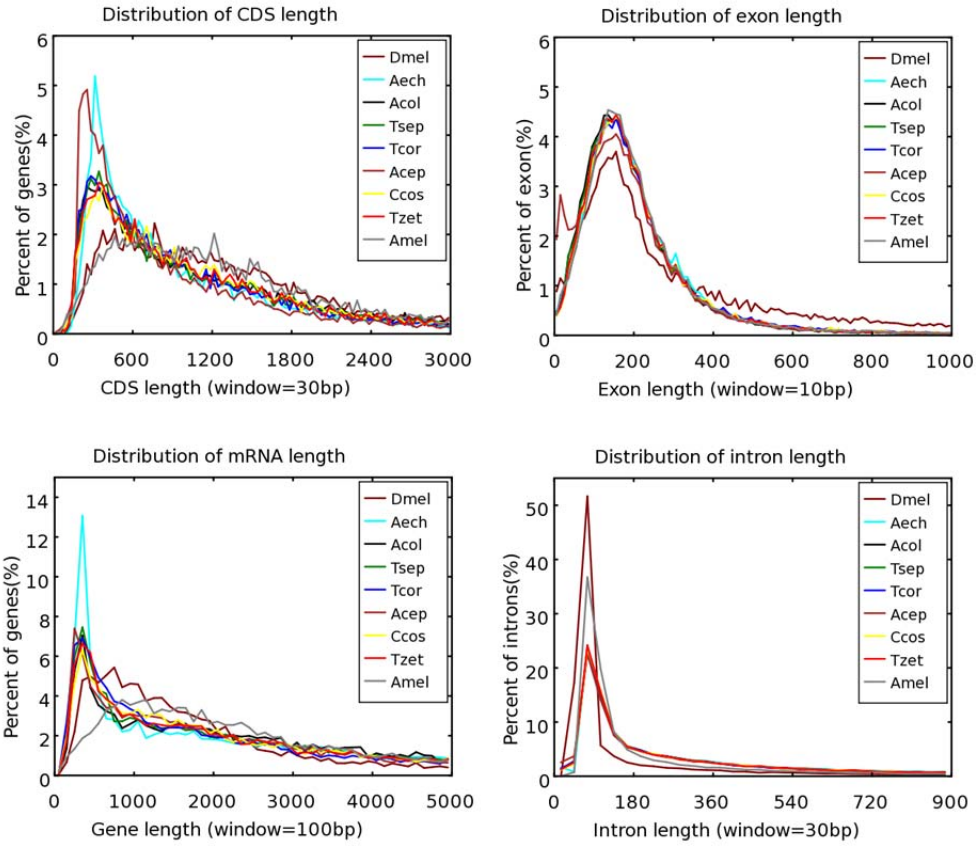

Supplementary Figure 7. Gene feature distributions
Length distributions for four general features of the final attine ant gene annotation sets: Coding sequences (CDS), exons, mRNAs, and introns. The corresponding distributions for *Apis mellifera* (Amel) and *Drosophila melanogaster* (Dmel) are included for comparison.

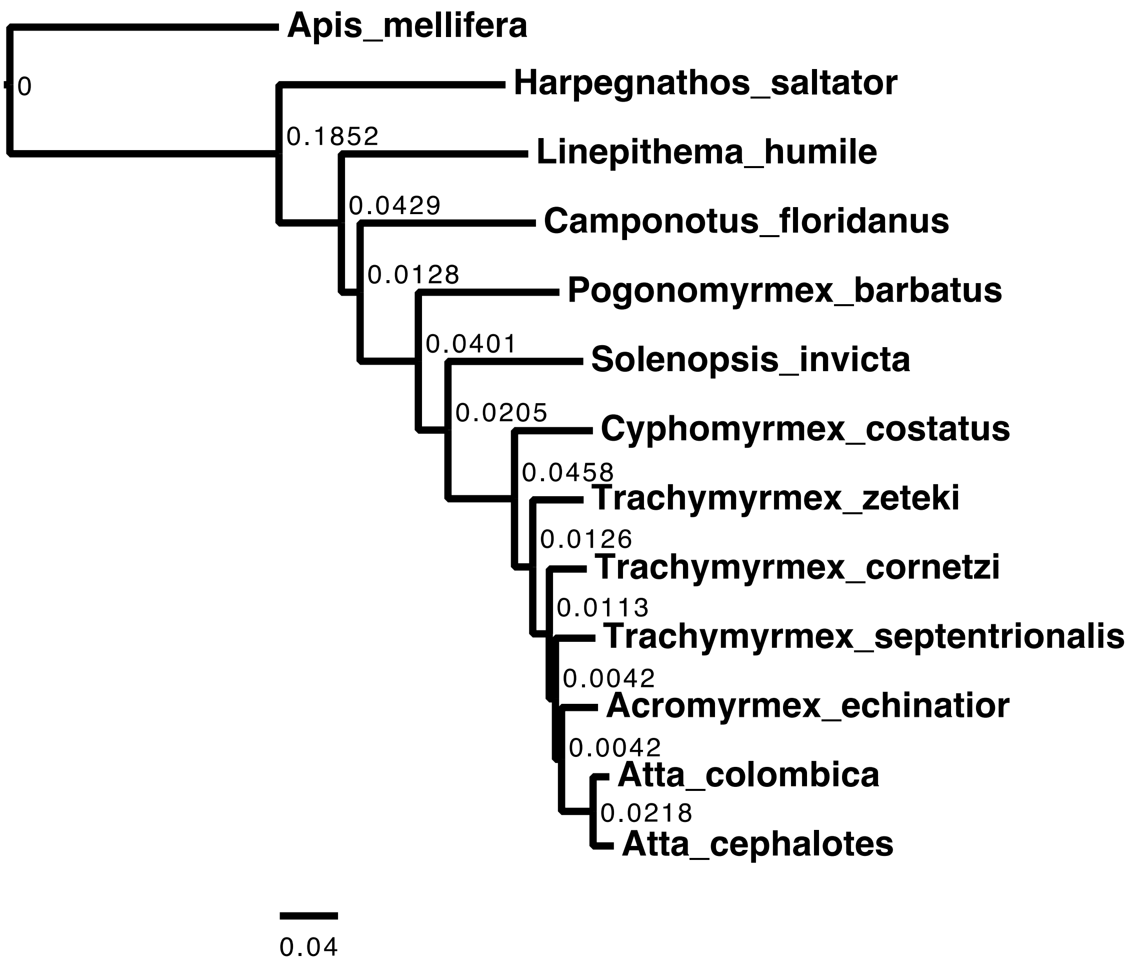

Supplementary Figure 8. Ant maximum-likelihood tree
The most likely tree resulting from a maximum-likelihood analysis of 1,886,151 amino acid sites (2795 loci parsed into 132 partitions) and 12 ant species and the honey bee *Ap. mellifera* as outgroup. All nodes were supported by bootstrap frequencies of 1.0. Scale bar and numbers on nodes indicate branch lengths (substitutions per site).

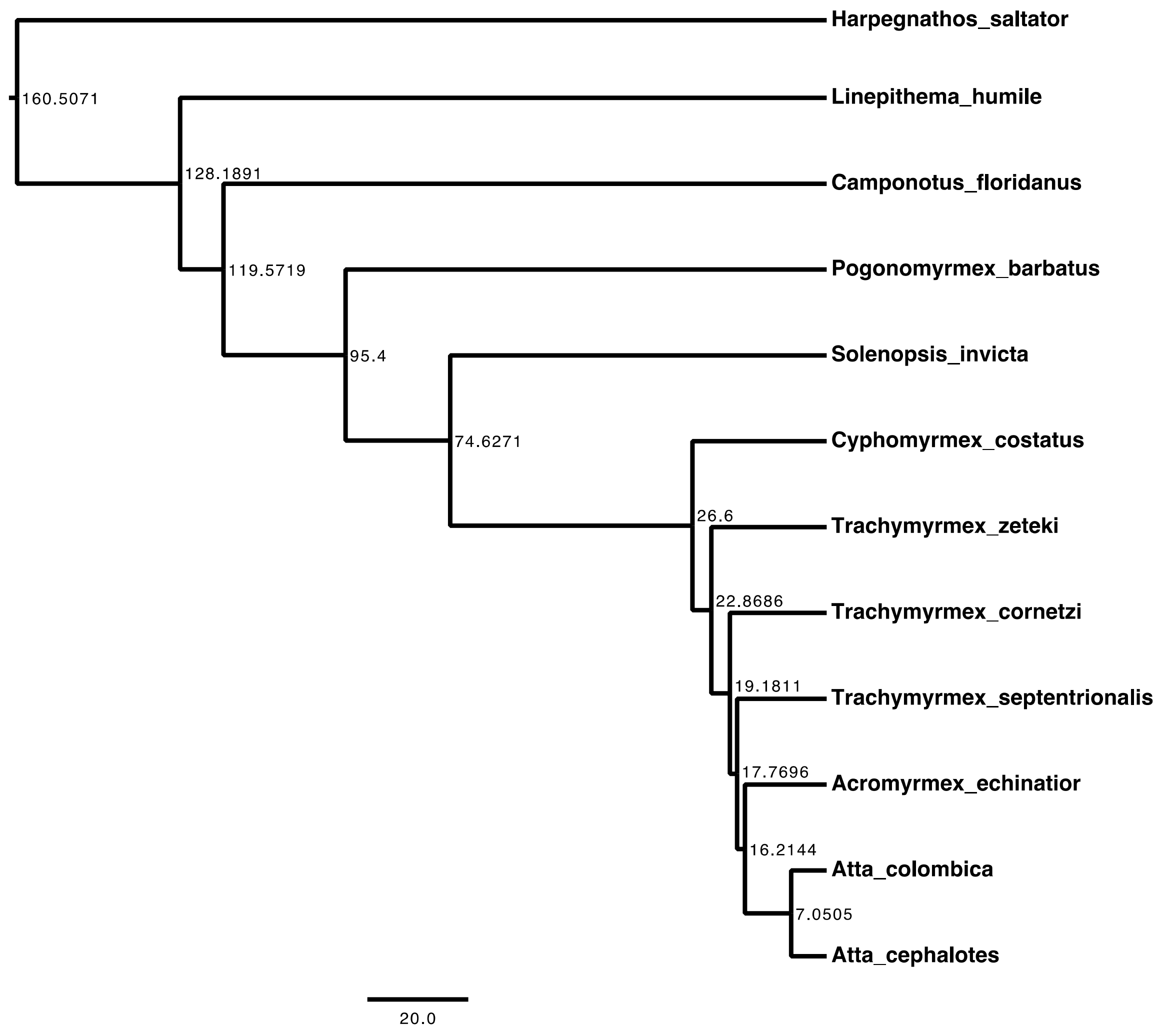

Supplementary Figure 9. Ant dated tree
A dated time tree for 12 ant species inferred after using a penalized likelihood approach. Scale bar and numbers on nodes indicate dates in millions of years before present.

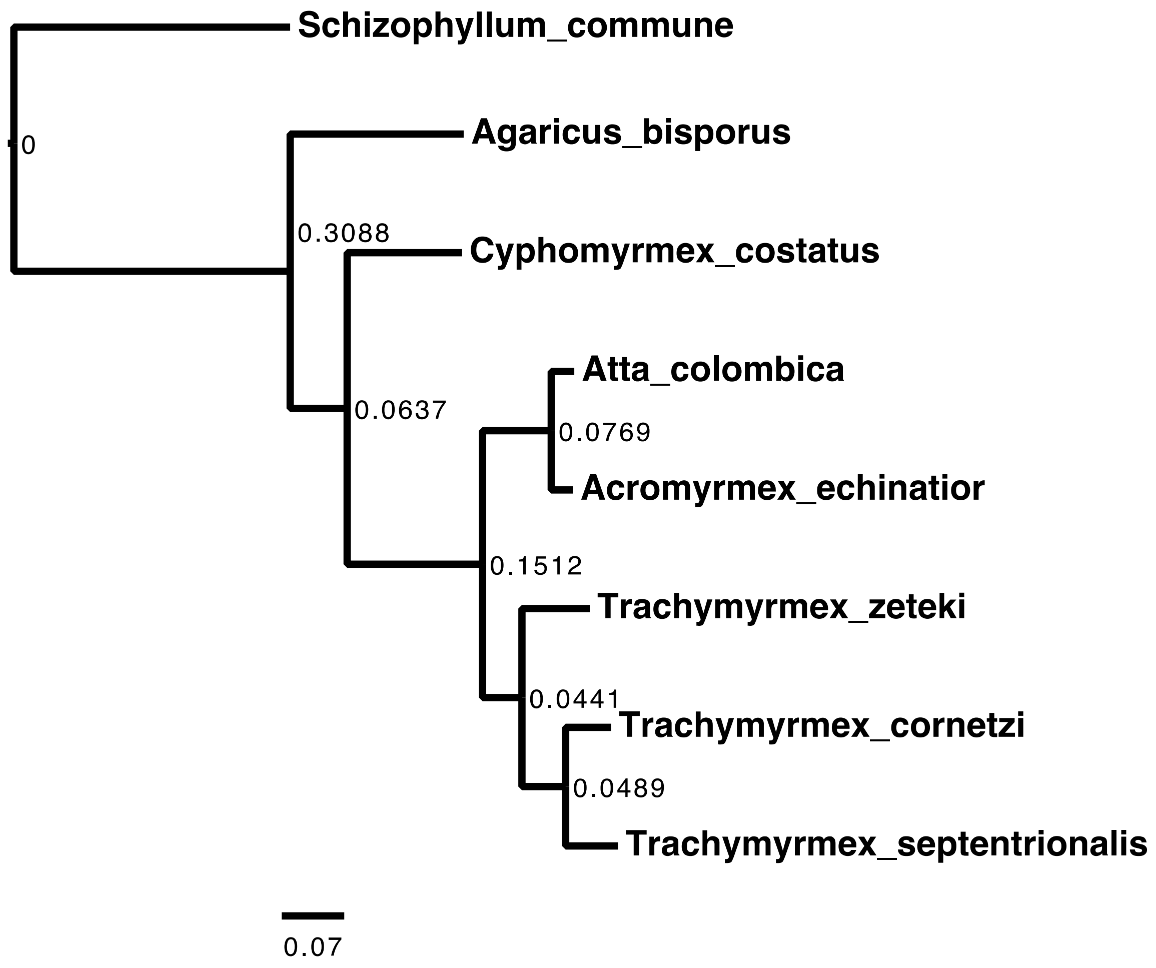

Supplementary Figure 10. Cultivar maximum-likelihood tree
The most likely tree resulting from a maximum-likelihood analysis of 825,686 amino acid sites (1075 loci parsed into 19 partitions) and 8 fungal taxa. All nodes were supported by bootstrap frequencies of 1.0. Scale bar and numbers on nodes indicate branch lengths (substitutions per site).

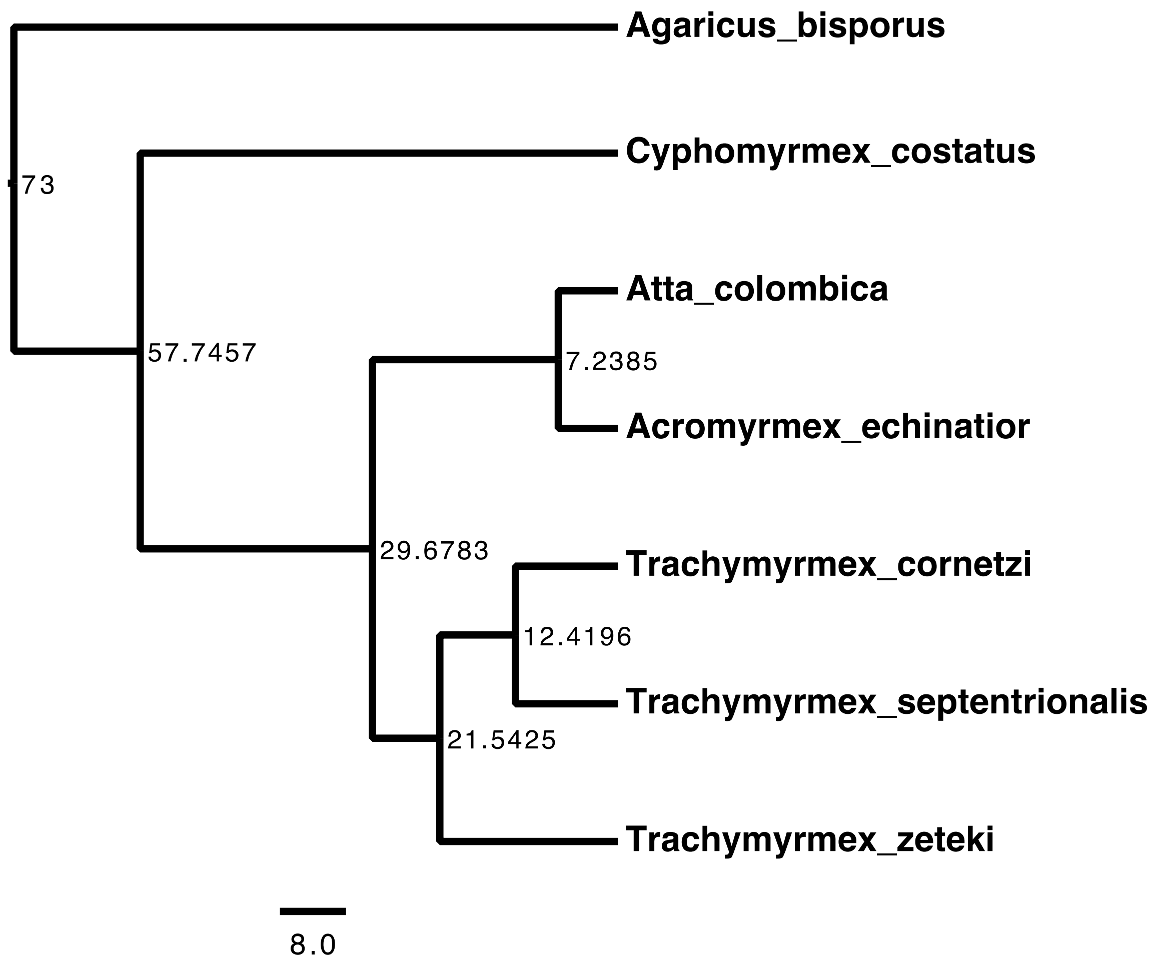

Supplementary Figure 11. Cultivar dated tree
A dated time tree for 7 fungal taxa inferred after using a penalized likelihood approach. Scale bar and numbers on nodes indicate dates in millions of years.

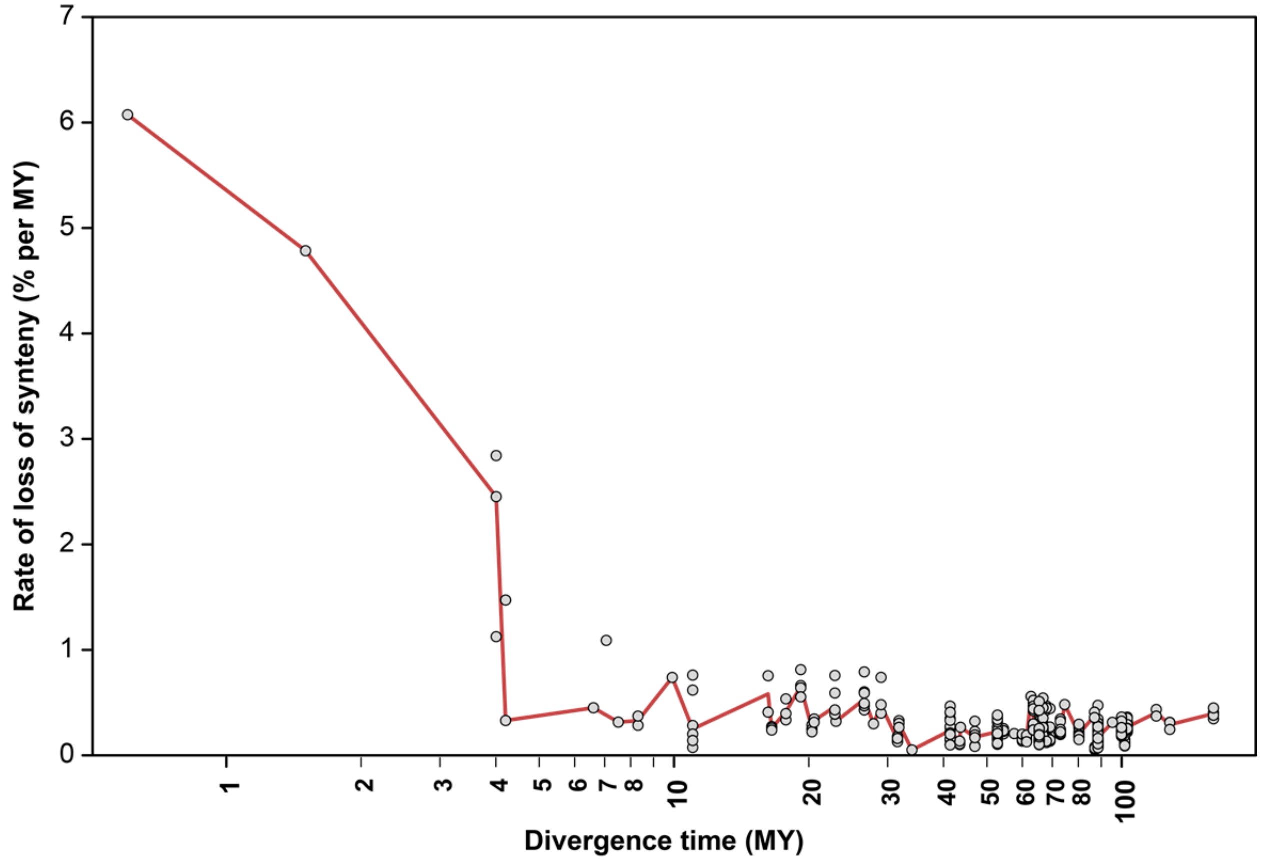

Supplementary Figure 12. Syntenty loss and divergence time
Relationship between divergence time and rate of loss of synteny for pairs of animal species. The red line is from a fitted LOWESS robust locally weighted regression (single iteration, local smoothing width (α) = 0.01).

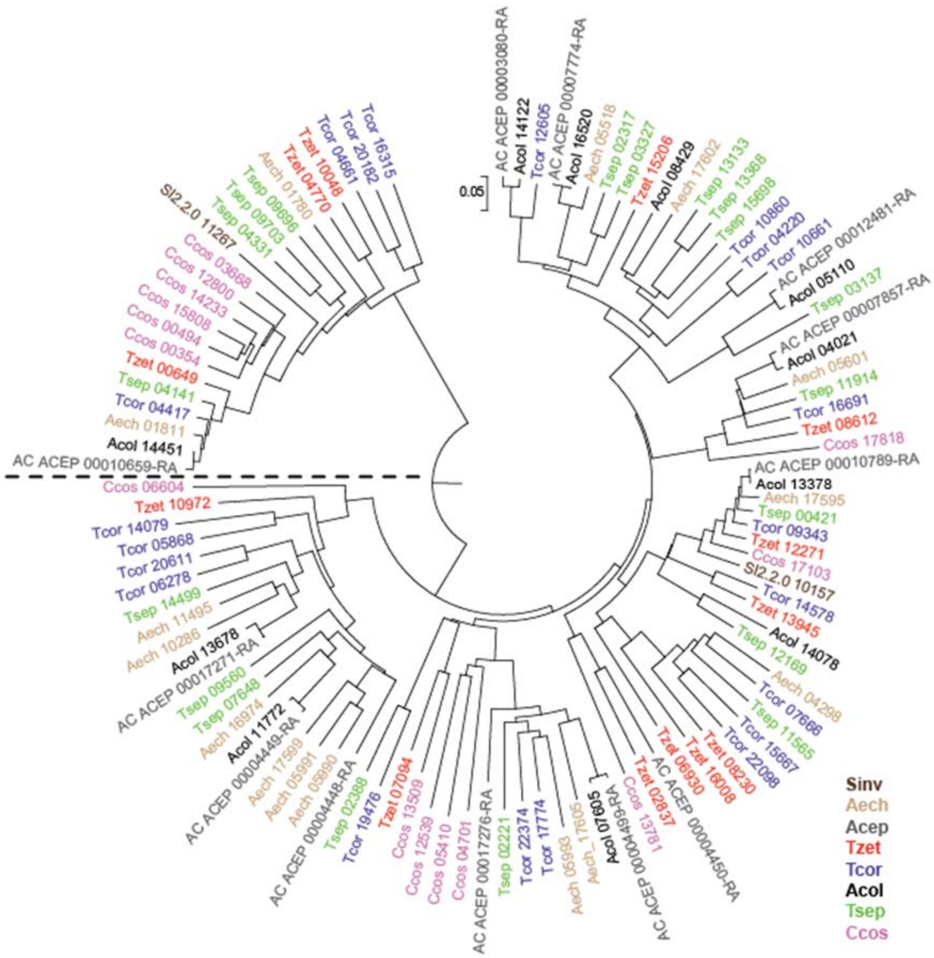

Supplementary Figure 13. M16 peptidase phylogenetic tree
M16 peptidase genes across attine ants with *Solenopsis invicta* (Sinv) as outgroup. Genes above the dashed line are insulin degrading enzymes. The rest belong to the *Nardilysin* gene family, which is significantly expanded in all attine ants.

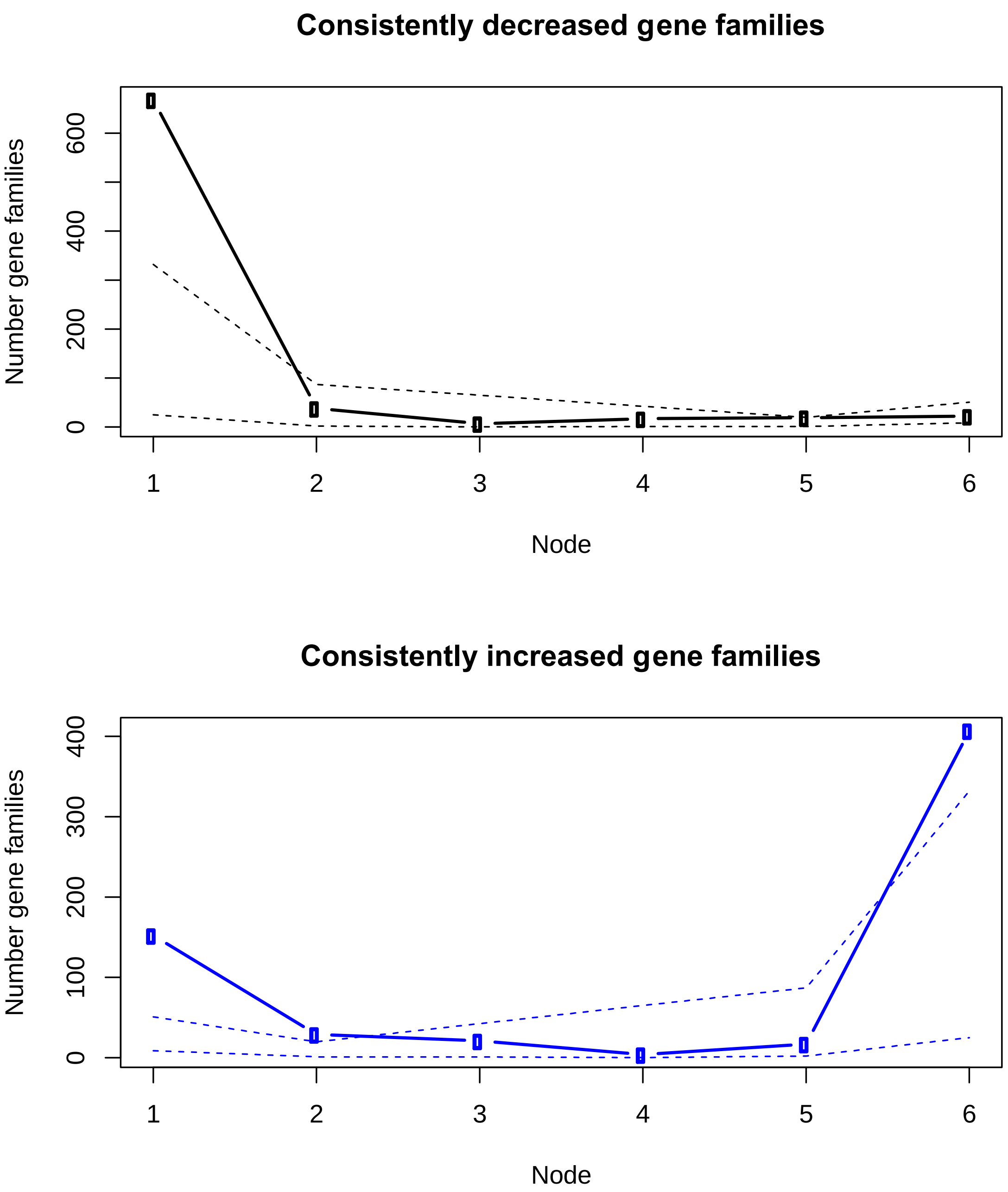

Supplementary Figure 14. Gene family decreases and increases
The number of consistently decreased (top, black) and increased (bottom, blue) gene families at different ancestral branches in the attine ant phylogeny. Dots connected with solid lines indicate the observed numbers. Dashed lines indicate 5th and 95th percentiles based on permutated data (see Supplementary methods). Node 1: The immediate ancestor of Ccos. 2: The ancestor of Tzet. 3: The ancestor of Tcor. 4: The ancestor of Tsep. 5: The ancestor of Aech. 6: The ancestor of the two *Atta* species.

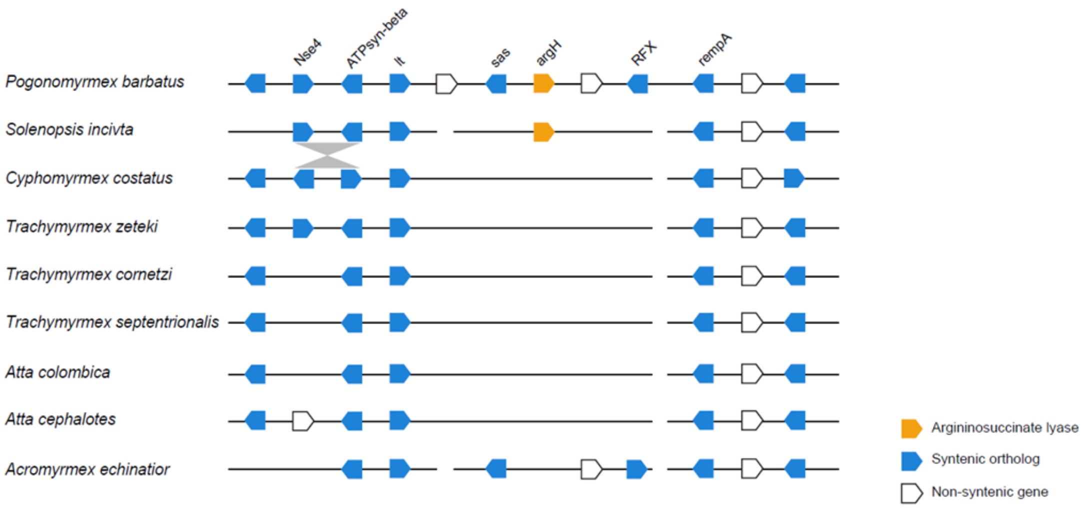

Supplementary Figure 15. Gene synteny around the argininosuccinate lyase gene
Gene synteny for seven attine and two outgroup ant species. Grey lines between syntenic genes of *S. invicta* and *C. costatus* denote gene inversions.

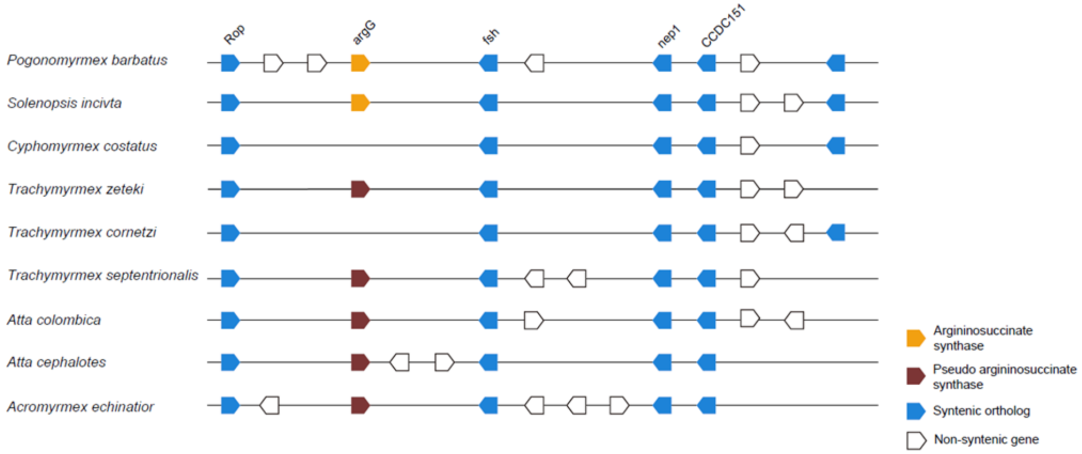

Supplementary Figure 16. Gene synteny around the argininosuccinate synthase gene
Gene synteny for seven attine and two outgroup ant species.

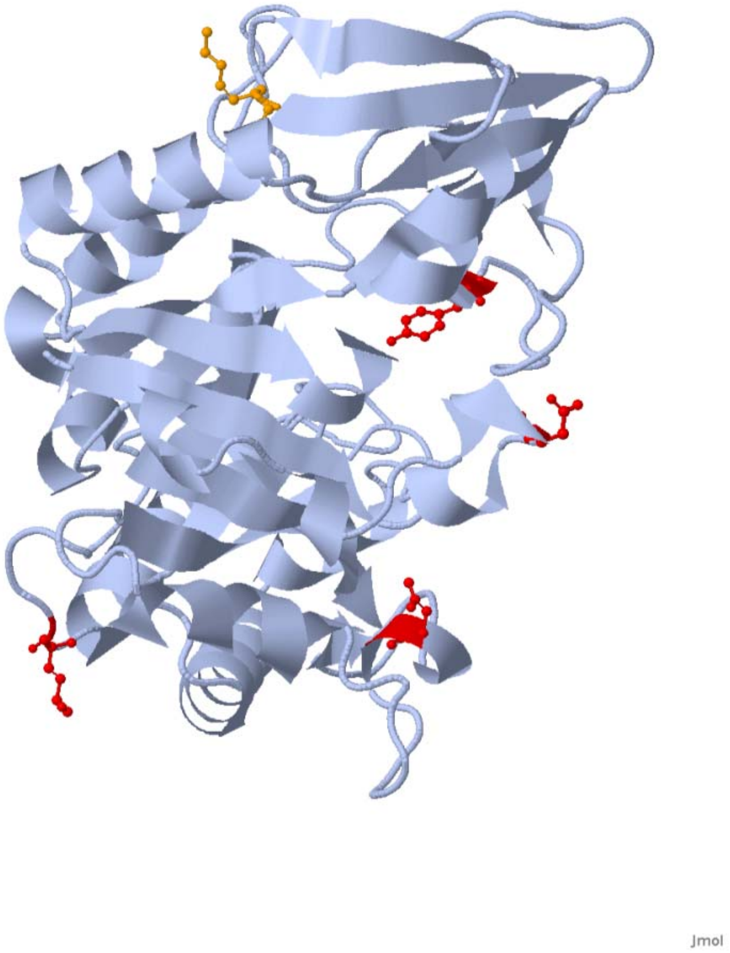

Supplementary Figure 17. Chitinase protein structure model
Homology-based structure model for the positively selected *At. cephalotes* chitinase. A cartoon view of the backbone is shown. Residues inferred to be positively selected in the ancestor of all attine ants are highlighted in red and their side chains shown as ball-and-stick models. A single additional residue has experienced positive selection in the ancestor of all higher attine ants and is plotted in orange. Positively selected sites occur primarily on the external surface of the protein.

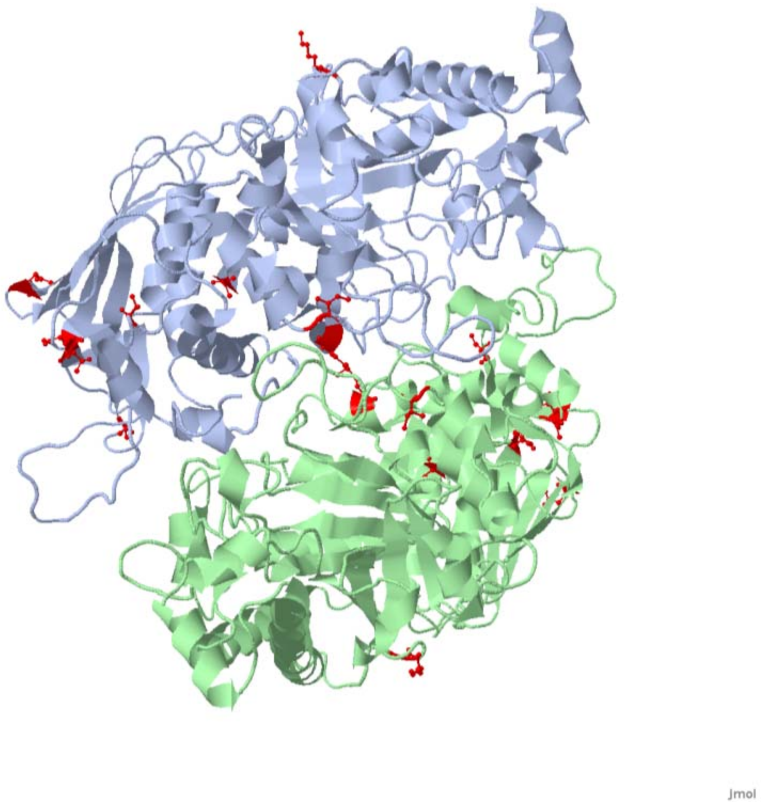

Supplementary Figure 18. Beta-hexosaminidase protein structure model
Homology-based structure model for the positively selected beta-hexosaminidase enzyme. A cartoon view of the backbone is shown. Residues inferred to be positively selected in the ancestor of all attine ants are highlighted in red and their side chains are shown as ball-and-stick models. Positively selected sites occur primarily on the external surfaces of the proteins.

Supplementary Tables

Supplementary Table 1. Size, scaffold N50, and depth of coverage for each genome assembly, and the total number of filtered bases (Gb) and assembled transcripts for each transcriptome.

**Ant-cultivar combination**		**Genomes**			**Transcriptomes**	
		Size (Mb)	N50 (Mb)	Depth (×)	Data (Gb)	#Transcripts
Atta colombica	ant	293	2.00	129	4.66	32,366
	fungus				4.99	117,446
Acromyrmex echinatior	fungus	107	NA	45	4.56	98,389
Trachymyrmex septentrionalis	ant	295	2.45	102	5.29	31,519
	fungus				3.18	126,024
Trachymyrmex cornetzi	ant	402	0.63	109	4.65	37,140
	fungus				4.49	147,902
Trachymyrmex zeteki	ant	269	1.33	82	4.06	27,124
	fungus				4.75	167,915
Cyphomyrmex costatus	ant	323	1.02	92	5.00	40,166
	fungus	138	0.1	27	4.44	81,243

Supplementary Table 2. NCBI BioProject, Genbank, and SRA accession numbers for the data generated in this study.

**Data set**	**Species**	**Bioproject**	**GenBank**	**SRA**
**Attine Genomes**	*A. colombica*	PRJNA292624	LKEW00000000	SRP070114
	T. septentrionalis	PRJNA292625	LKEZ00000000	SRP070115
	T. cornetzi	PRJNA292627	LKEY00000000	SRP070108
	T. zeteki	PRJNA292628	LKFA00000000	SRP070107
	C. costatus	PRJNA292630	LKEX00000000	SRP070116
**Attine Transcriptomes**	*A. colombica*	PRJNA315800	-	SRP072100
	T. septentrionalis	PRJNA315799	-	SRP072103
	T. cornetzi	PRJNA315801	-	SRP072099
	T. zeteki	PRJNA315802	-	SRP072098
	C. costatus	PRJNA315803	-	SRP072097
**Fungal Genome**	*C. costatus* cultivar	PRJNA295288	LSHD00000000	SRP070118
**Fungal Transcriptomes**	*A. colombica* cultivar	PRJNA298477	GEHH00000000	SRP065006
	A. echinatior cultivar	PRJNA298481	GEHD00000000	SRP065007
	T. septentrionalis cultivar	PRJNA298478	GEHI00000000	SRP065010
	T. cornetzi	PRJNA298479	GEHG00000000	SRP065009

	cultivar			
	T. zeteki cultivar	PRJNA298480	GEHE00000000	SRP065011
	C. costatus cultivar	PRJNA299485	GEHF00000000	SRP070117

Supplementary Table 3**.** Overview of the amount of sequencing data used for the genome assemblies.

Species	Estimated genome size (Mb)	Total raw data(Gb)	Total clean data(Gb)	Sequence coverage (×)	Physical coverage (×)
**Ants**					
Acol	280.2	42.30	36.23	129.29	1924.17
Tsep	294.4	36.19	30.07	102.14	1668.04
Tcor	396.1	54.32	43.15	108.94	1853.61
Tzet	294.8	28.87	24.13	81.88	1032.25
Ccos	318.5	34.40	29.41	92.36	1665.68
**Fungi**					
Aech-F	107.1	7.14	6.29	58.70	199.67
Ccos-F	137.8	10.37	6.85	49.74	510.16

Supplementary Table 4**.** Ant genome size estimates based on 17-mer analysis. The reads used for the k-mer analysis were all from small insert size libraries.

Species	k-mer	#k-mer	Peak depth	Genome size
**Acol**	17	12,609,581,687	45	280,212,926
**Tsep**	17	12,364,256,268	42	294,387,054
**Tcor**	17	18,222,575,628	46	396,142,948
**Tzet**	17	13,853,424,144	47	294,753,705
**Ccos**	17	15,286,050,715	48	318,459,389

Supplementary Table 5**.** Fungal cultivar genome size estimates based on 17-mer analysis. The reads used for k-mer analysis were all from small insert size libraries.

Species	K-mer	#K-mer	Peak depth	Genome size (bp)
**Aech-F**	17	4,821,739,644	45	107,149,769
**Ccos-F**	17	3,720,481,865	27	137,795,624

Supplementary Table 6**.** Bacterial sequence contamination in the ant genome assemblies as determined by sequence homology searches.

Species	Total length (bp)	Number of scaffolds
**Acol**	0	0
**Tsep**	1,659,586	20
**Tcor**	704,151	63
**Tzet**	886,119	3
**Ccos**	6,706,714	393

Supplementary Table 7**.** Overall descriptive statistics of the ant and fungal genome assemblies.

	Species	Total size (Mb)	Scaffold N50 (bp)	Contig N50 (bp)	Longest scaffold	Longest contig
**Ants**						
	Acol	292.91	2,037,154	14,996	8,263,949	117,333
	Tsep	295.39	2,447,259	14,505	10,371,161	129,122
	Tcor	402.02	632,812	12,443	7,295,537	208,836
	Tzet	269.37	1,333,945	18,987	6,036,021	147,902
	Ccos	323.12	1,016,465	27,202	6,852,325	271,308
**Fungi**						
	Aech-F	151.79	805	717	16,265	14,331
	Ccos-F	125.81	107,067	7,342	856,993	73,276

Supplementary Table 8. Genomic repeat content for the five ant- and single fungal genome assemblies. Numbers are percentages of total assembly size.

Type	Tzet	Tcor	Acol	Tsep	Ccos	Ccos-F
DNA	3.42	13.31	16.69	9.27	7.83	2.95
LINE	0.94	4.70	1.21	2.37	3.16	9.09
SINE	0.18	0.21	0.04	0.16	0.01	0.006
LTR	1.15	4.95	1.60	2.08	4.89	36.7
Satellite	1.84	2.46	2.55	1.94	2.36	0.3
Other	0.00	0.00	0.00	0.00	0.00	0.0001
Unknown	20.30	19.45	8.54	10.99	15.37	0.0006
**Total**	**24.95**	**42.12**	**31.76**	**23.88**	**33.97**	**46.4**

Supplementary Table 9. Total sizes of high-frequency repeats (bp) in attine ant genome assemblies.

Repeat family	Acol	Tsep	Tcor	Tzet	Ccos
DNA/TcMar-Mariner	41,177,317	12,412,602	16,248,750	1,380,383	4,650,506
LTR/Gypsy	2,043,949	2,778,074	11,618,114	1,472,591	8,304,674
LINE/Penelope	2,067,910	4,348,089	9,762,463	1,131,558	5,448,355
DNA/Maverick	3,239,024	4,502,365	9,705,341	1,272,556	4,736,121
DNA/En-Spm	2,424,258	4,396,665	5,314,060	912,350	2,926,260
LTR/Pao	922,850	1,242,836	4,804,113	708,988	3,735,329
DNA/Sola	629,296	1,122,453	4,166,108	529,853	2,090,237
LINE/L2	513,104	660,510	3,713,546	319,283	1,415,312
DNA/TcMar-Tc1	1,937,533	1,732,123	3,640,197	1,097,494	1,188,501
DNA/TcMar-Marin	5,621,351	3,824,012	3,486,909	813,516	970,619
LINE/R1	282,661	489,800	2,185,247	410,414	510,822
DNA/MuDR	290,821	325,537	2,047,337	237,155	505,544
DNA/P	170,577	244,993	2,035,301	433,816	834,691
DNA/Harbinger	77,359	98,444	1,954,003	309,890	671,882
DNA/Helitron	251,500	314,461	1,942,396	371,751	774,787
LTR/Copia	755,428	1,593,831	1,831,215	482,621	2,744,775

Supplementary Table 10**.** Homology-based gene predictions in attine ant genomes. The numbers of predictions/hits generated for each assembly (columns) by each query-genome (rows) are given. The number of merged predictions for the two combined ant query-genomes are also provided.

		**Tzet**	**Tcor**	**Acol**	**Tsep**	**Ccos**
H.sapiens		3,220	4,258	3,964	4,212	4,168
D.melanogaster		4,951	5,015	4,813	4,987	4,986
C.elegans		2,647	2,774	2,618	2,662	2,726
A.mellifera		9,032	10,265	9,585	8,787	9,218
**ANT1**	*H.saltator*	14,190	23,777	10,379	11,832	18,130
	C.floridanus	14,950	27,179	11,216	12,840	20,161
	Ac.echinatior	16,191	22,969	13,828	15,247	17,893
	Merged	21,446	39,335	16,606	19,101	27,902
**ANT2**	*At.cephalotes*	16,783	21,180	15,290	16,471	18,028
	S.invicta	15,635	23,325	13,473	15,685	18,399
	L.humile	13,662	16,546	12,568	13,474	14,981
	P.barbatus	15,211	17,974	13,911	15,324	16,422
	Merged	22,188	34,485	20,256	22,933	26,616

Supplementary Table 11**.** Number of *de novo* gene predictions in attine genomes as obtained by two different methods, as well as the number of combined predictions.

**Method**	**Acol**	**Tsep**	**Tcor**	**Tzet**	**Ccos**
AUGUSTUS	17,095	17,593	18,046	13,866	17,736
SNAP	46,818	44,545	55,952	45,419	44,461
**Merged set**	13,525	13,953	14,752	11,304	13,914

Supplementary Table 12. Overview of the amounts of RNA-seq data used for ant genome annotation.

**Species**	**Total reads (Mb)**	**Reads mapped to genome (Mb)**	**Total bases (Gb)**	**Bases mapped to genome (Gb)**	**Number of assembled transcripts**
Acol	58.68	51.77	5.28	4.66	32,366
Tsep	64.71	58.77	5.82	5.29	31,519
Tcor	57.39	51.68	5.17	4.65	37,140
Tzet	60.03	45.14	5.40	4.06	27,124
Ccos	60.94	55.52	5.49	5.00	40,166

Supplementary Table 13**.** Integration of ant gene predictions to generate final gene sets. For each species the number of predictions by GLEAN, improved by RNA-seq data, and combined are given.

	Acol	Tsep	Tcor	Tzet	Ccos
GLEAN	16,530	20,061	31,313	19,287	23,523
Improved by RNA-seq	17,515	20,764	32,498	19,839	24,933
**Final gene set**	14,345	15,575	19,827	15,530	16,468

Supplementary Table 14**.** Functional annotation of ant protein coding genes. For each species the number of genes annotated by each of four different methods are given, as well as the total number of genes with inferred functional annotation.

Method	Acol	Tsep	Tcor	Tzet	Ccos
KEGG	2,892	2,983	3,133	2,983	3,094
IPR	8,302	8,846	10,443	9,043	10,130
GO	6,855	7,230	8,414	7,401	8,166
SwissProt	8,226	8,744	9,296	8,613	9,161
**Total**	8,824	9,597	11,280	9,684	10,841

Supplementary Table 15**.** Annotation of four major classes of non-protein-coding genes in attine ant genomes. For each species (columns) the number of miRNAs, tRNAs, rRNAs and snRNAs (rows) are given.

RNA class	Acol	Tsep	Tcor	Tzet	Ccos
miRNA	85	97	400	109	298
tRNA	213	466	1005	518	333
rRNA	27	19	125	15	229
snRNA	26	44	36	30	37

Supplementary Table 16**.** The amount of fungal transcriptome sequencing (RNA-Seq) data generated for this study.

Fungal cultivar	Raw data		Filtered data	
	**Read length**	**Base Number (Mbp)**	**Read length**	**Base Number (Mbp)**
Acol-F	90	4995	80	3362
Aech-F	90	4560	80	3063
Tcor-F	90	4490	80	3121
Tsep-F	90	3183	80	1930
Tzet-F	90	4752	80	3254
Ccos-F	90	4443	80	2281

Supplementary Table 17**.** The number non-redundant genes identified in fungal cultivars.

Transcriptome	Non-redundant genes#
Acol-F	8360
Aech-F	16180
Tcor-F	9003
Tsep-F	8032
Tzet-F	7534
Ccos-F	9023
**Genome**	**Genes#**
Ccos-F	13348

Supplementary Table 18**.** The number, total size, and percentage of genomes that are classified as segmental duplications (SDs) in the five attine ant genome assemblies. The total numbers of genes contained within these SDs are also indicated.

Segmental duplicates	Acol	Tsep	Tcor	Tzet	Ccos
#SDs	4,059	11,185	20,442	5,928	10,266
Total length (Mb)	6.01	10.21	17.77	6.54	13.13
% of genome	2.05	3.46	4.42	2.43	4.06
#Genes involved	178	380	1107	454	865

Supplementary Table 19**.** Gene family clustering of ant and insect outgroup gene sets. For each species (rows) the total number of genes (#Genes), the number of genes assigned to clusters (#Clustered genes), the number of gene clusters (#Clusters), and the number of unclustered genes (#Unclustered genes) are given.

**Species**	**#Genes**	**#Clustered genes**	**#Clusters**	**#Unclustered genes**
**15 insects**				
Apis mellifera	10,660	9,701	8,610	959
Drosophila melanogaster	13,689	9,973	7,429	3,716
Nasonia vitripennis	17,084	14,689	8,797	2,395
Camponotus floridanus	16,356	13,046	10,751	3,310
Harpegnathos saltator	17,191	13,680	10,439	3,511
Linepithema humile	15,992	13,242	11,636	2,750
Pogonomyrmex barbatus	17,015	13,593	12,118	3,422
Solenopsis invicta	16,522	13,411	11,486	3,111
Cyphomyrmex costatus	16,468	15,266	12,994	1,202
Trachymyrmex zeteki	15,530	14,552	13,206	978
Trachymyrmex cornetzi	19,827	17,608	14,429	2,219
Trachymyrmex septentrionalis	15,575	14,454	13,157	1,121
Acromyrmex echinatior	17,280	15,302	13,201	1,978
Atta colombica	14,345	13,511	12,809	834
Atta cephalotes	18,021	14,810	13,211	3,211

Supplementary Table 20**.** Genomes used for pairwise synteny calculations.

Species name	**Data source**
**Attine ants**	
Atta cephalotes	Hymenoptera Genome Database
Atta colombica	This study
Acromyrmex echinatior	Hymenoptera Genome Database
Cyphomyrmex costatus	This study
Trachymyrmex cornetzi	This study
Trachymyrmex septentrionalis	This study
Trachymyrmex zeteki	This study
**Published ants**	
Camponotus floridanus	Hymenoptera Genome Database
Harpegnathos saltator	Hymenoptera Genome Database
Linepithema humile	Hymenoptera Genome Database
Pogonomyrmex barbatus	Hymenoptera Genome Database
Solenopsis invicta	Hymenoptera Genome Database

Drosophila	
Drosophila ananassae	FlyBase
Drosophila erecta	FlyBase
Drosophila grimshawi	FlyBase
Drosophila melanogaster	FlyBase
Drosophila mojavensis	FlyBase
Drosophila persimilis	FlyBase
Drosophila pseudoobscura	FlyBase
Drosophila sechellia	FlyBase
Drosophila simulans	FlyBase
Drosophila virilis	FlyBase
Drosophila willistoni	FlyBase
Drosophila yakuba	FlyBase
**Primates**	
Callithrix jacchus	Ensembl
Gorilla gorilla	Ensembl
Homo sapiens	Ensembl
Macaca mulatta	Ensembl
Nomascus leucogenys	Ensembl
Otolemur garnettii	Ensembl
Pan troglodytes	Ensembl
Pongo abelii	Ensembl
**Birds**	
Anas platyrhynchos	Zhang et al. 2014
Aptenodytes forsteri	Zhang et al. 2014
Calypte anna	Zhang et al. 2014
Chaetura pelagica	Zhang et al. 2014
Charadrius vociferus	Zhang et al. 2014
Columba livia	Zhang et al. 2014
Corvus brachyrhynchos	Zhang et al. 2014
Cuculus canorus	Zhang et al. 2014
Egretta garzetta	Zhang et al. 2014
Falco peregrinus	Zhang et al. 2014
Gallus gallus	Zhang et al. 2014
Geospiza fortis	Zhang et al. 2014
Haliaeetus leucocephalus	Zhang et al. 2014
Manacus vitellinus	Zhang et al. 2014
Meleagris gallopavo	Zhang et al. 2014
Melopsittacus undulatus	Zhang et al. 2014
Nipponia nippon	Zhang et al. 2014
Ophisthocomus hoazin	Zhang et al. 2014
Picoides pubescens	Zhang et al. 2014

Pygoscelis adeliae	Zhang et al. 2014
Struthio camelus	Zhang et al. 2014
Taeniopygia guttata	Zhang et al. 2014
**Mosquitoes**	
Anopheles albimanus	Vectorbase
Anopheles arabiensis	Vectorbase
Anopheles atroparvus	Vectorbase
Anopheles dirus	Vectorbase
Anopheles epiroticus	Vectorbase
Anopheles farauti	Vectorbase
Anopheles funestus	Vectorbase
Anopheles gambiae	Vectorbase
Anopheles merus	Vectorbase
Anopheles minimus	Vectorbase
Anopheles quadriannulatus	Vectorbase
Anopheles stephensi	Vectorbase

Supplementary Table 21**.** Significantly expanded gene families in all attine ants relative to outgroups. The number of genes belonging to each family in each species is given.

Fam_name	Acol	Acep	Aech	Tsep	Tcor	Tzet	Ccos	Sinv	Pbar
**TOM70**	5	3	3	2	6	5	7	1	1
**Nardilysin**	10	11	13	15	17	10	8	1	1

Supplementary Table 22**.** Enriched GO categories among genes with significantly increased dN/dS ratios in higher attine ants. P-values are corrected for false discovery rate.

GO	P-value	Description
30286	1.1946E-5	dynein complex
15630	1.4738E-5	microtubule cytoskeleton
5875	1.2387E-4	microtubule associated complex
3824	1.4861E-4	catalytic activity
7017	1.5655E-4	microtubule-based process
9987	2.7747E-4	cellular process
44430	2.7747E-4	cytoskeletal part
8152	2.9687E-4	metabolic process
1882	5.3706E-4	nucleoside binding
30554	7.3636E-4	adenyl nucleotide binding
1883	7.3636E-4	purine nucleoside binding
7018	1.0849E-3	microtubule-based movement
3777	1.0849E-3	microtubule motor activity

44281	1.3675E-3	small molecule metabolic process
48037	1.7441E-3	cofactor binding
166	1.7441E-3	nucleotide binding
17076	4.3759E-3	purine nucleotide binding
50662	6.3333E-3	coenzyme binding
3774	6.6455E-3	motor activity
9058	8.2712E-3	biosynthetic process
6084	9.0515E-3	acetyl-CoA metabolic process
5524	9.0515E-3	ATP binding
32559	9.3275E-3	adenyl ribonucleotide binding
5856	9.3275E-3	cytoskeleton
16887	1.1673E-2	ATPase activity
43228	1.4319E-2	non-membrane-bounded organelle
43232	1.4319E-2	intracellular non-membrane-bounded organelle
9165	1.5731E-2	nucleotide biosynthetic process
44237	1.5731E-2	cellular metabolic process
55114	1.7678E-2	oxidation reduction
44283	1.7678E-2	small molecule biosynthetic process
16616	1.9354E-2	oxidoreductase activity, acting on the CH-OH group of donors, NAD or NADP as acceptor
9152	2.0174E-2	purine ribonucleotide biosynthetic process
16491	2.2378E-2	oxidoreductase activity
34654	2.2757E-2	nucleobase, nucleoside, nucleotide and nucleic acid biosynthetic process
34404	2.2757E-2	nucleobase, nucleoside and nucleotide biosynthetic process
4594	2.4554E-2	pantothenate kinase activity
6091	2.4554E-2	generation of precursor metabolites and energy
9201	2.4554E-2	ribonucleoside triphosphate biosynthetic process
9206	2.4554E-2	purine ribonucleoside triphosphate biosynthetic process
9145	2.4554E-2	purine nucleoside triphosphate biosynthetic process
9142	2.4554E-2	nucleoside triphosphate biosynthetic process
44249	2.6560E-2	cellular biosynthetic process
32555	2.6811E-2	purine ribonucleotide binding
32553	2.6811E-2	ribonucleotide binding
9260	2.6811E-2	ribonucleotide biosynthetic process
9109	2.7395E-2	coenzyme catabolic process
6099	2.7395E-2	tricarboxylic acid cycle
46356	2.7395E-2	acetyl-CoA catabolic process

6732	2.7395E-2	coenzyme metabolic process
16646	3.0484E-2	oxidoreductase activity, acting on the CH-NH group of donors, NAD or NADP as acceptor
55085	3.1248E-2	transmembrane transport
51187	3.2885E-2	cofactor catabolic process
16817	3.6329E-2	hydrolase activity, acting on acid anhydrides
6164	3.8278E-2	purine nucleotide biosynthetic process
5874	3.9023E-2	microtubule
50660	3.9907E-2	FAD binding
16614	4.4839E-2	oxidoreductase activity, acting on CH-OH group of donors
8762	4.6860E-2	UDP-N-acetylmuramate dehydrogenase activity
16668	4.6860E-2	oxidoreductase activity, acting on sulfur group of donors, NAD or NADP as acceptor
42180	4.8928E-2	cellular ketone metabolic process

Supplementary Table 23**.** Enriched GO categories among genes with significantly increased dN/dS ratios in leaf-cutting ants. P-values are corrected for false discovery rate.

GO	P-value	Description
30286	4.97E-04	dynein complex
5875	1.18E-03	microtubule associated complex
3824	5.33E-03	catalytic activity
3777	9.35E-03	microtubule motor activity
15630	9.35E-03	microtubule cytoskeleton
6402	1.07E-02	mRNA catabolic process
7018	1.31E-02	microtubule-based movement
6401	1.33E-02	RNA catabolic process
3774	2.62E-02	motor activity
7017	2.62E-02	microtubule-based process
44430	3.19E-02	cytoskeletal part

Supplementary Table 24. IPR domains absent in all domesticated higher attine ant cultivars. G behind abbreviated species names denotes genomic counts, whereas T denotes transcriptomic counts.

IPR	Acep-F G	Acol-F T	Aech-F G	Aech-F T	Tcor-F T	Tsep-F T	Tzet-F T	Ccos-F G	Ccos-FT	IPR
IPR001466	0	0	0	0	0	0	0	6	5	Beta-lactamase-related
IPR002196	0	0	0	0	0	0	0	4	2	Glycoside hydrolase, family 24
IPR002642	0	0	0	0	0	0	0	1	1	Lysophospholipase, catalytic domain
IPR004875	0	0	0	0	0	0	0	2	0	DDE superfamily endonuclease, CENP-B-like
IPR005198	0	0	0	0	0	0	0	2	2	Glycoside hydrolase, family 76
IPR005269	0	0	0	0	0	0	0	1	1	Cytokinin riboside 5'-monophosphate phosphoribohydrolase LOG
IPR005337	0	0	0	0	0	0	0	1	1	ATPase, P-loop-containing
IPR007541	0	0	0	0	0	0	0	1	1	Uncharacterised protein family, basic secretory protein
IPR007822	0	0	0	0	0	0	0	1	1	Lanthionine synthetase C-like
IPR008564	0	0	0	0	0	0	0	1	1	Protein of unknown function DUF846, eukaryotic
IPR008906	0	0	0	0	0	0	0	7	2	HAT dimerisation
IPR009297	0	0	0	0	0	0	0	1	1	Protein of unknown function DUF952
IPR010435	0	0	0	0	0	0	0	2	2	Peptidase S8A, DUF1034 C-terminal
IPR010686	0	0	0	0	0	0	0	1	1	Protein of unknown function DUF1264
IPR014870	0	0	0	0	0	0	0	4	4	Domain of unknown function DUF1793
IPR018502	0	0	0	0	0	0	0	1	1	Annexin repeat
IPR021851	0	0	0	0	0	0	0	1	1	Protein of unknown function DUF3455
IPR023128	0	0	0	0	0	0	0	1	1	Protein N-terminal glutamine amidohydrolase, alpha beta roll
IPR024589	0	0	0	0	0	0	0	3	3	Fungal ligninase, C-terminal
IPR025340	0	0	0	0	0	0	0	6	6	Protein of unknown function DUF4246

Supplementary Table 25**.** Names and accession numbers for the ligninases and surrounding genes in the investigated free living and farmed fungal species.

**Fungal species**	**Protein**	**Accession number**
Agaricus bisporus	Putative membrane permease	XP_006460926
	Ligninase	XP_006460927
	DNA Primase	XP_006460928
Cyphomyrmex costatus symbiont	Putative membrane permease	CCG006738.1
	Ligninase	CCG006739.1
	Ligninase	CCG006740.1
	Ligninase	CCG006741.1
	DNA Primase	CCG006742.1
Leucoagaricus gongylophorus	Putative membrane permease	jgiLeugo1875
	DNA Primase	jgiLeugo1873

Supplementary Table 26**.** Positively selected sites in the attine ant chitinase enzyme. Probability refers to posterior probability (BEB) from the Branch Site test of attine sequences versus the non-attine outgroups, except for *, which refers to a test between higher attine ants versus the lower attine *C. costatus* and the non-attine outgroups combined. Position refers to the site in the *At. cephalotes* sequence. Attines refers to the amino acid in the attines/higher attines. Outgroups refers to the corresponding ancestral amino acids.

**Probability**	**Position**	**Attines**	**Outgroups**
0.987	36	asparagine	proline
0.981	74	asparagine	glutamine
0.951	175	lysine	aspartic acid
0.993	297	tyrosine	methionine
0.988*	321	lysine	arginine

Supplementary Table 27**.** Positively selected sites in the attine ant beta-hexosaminidase. See the legend for Supplementary Table 26 for details.

**Probability**	**Position**	**Attines**	**Outgroups**
0.983	26	threonine	glutamine
0.959	43	threonine	valine
0.993	92	asparagine	glycine
0.980	123	asparagine	valine
0.984	157	serine	asparagine
0.968	201	alanine	methionine
0.983	407	lysine	alanine
0.983	461	isoleucine	lysine
0.985	506	methionine	serine

Supplementary Table 28**.** Sequence IDs for the positively selected chitinase and beta-hexosaminidase orthologous groups. Numbers in parenthesis indicate the sequences used for calculation of average residue weights and isoelectric points.

**Species**	**Chitinase**	**Beta-hexosaminidase**
**Attines**		
Atta cephalotes	XP_012061787.1 (22-363)	XP_012054348.1 (92-585)
Atta colombica	Acol_13972 (22-363)	Acol_09532 (92-585)
Acromyrmex echinatior	XP_011049876.1 (22-363)	XP_011050146.1 (61-554)
Trachymyrmex septentrionalis	Tsep_04708 (23-365)	Tsep_11452 (92-585)
Trachymyrmex cornetzi	Tcor_13983 (22-365)	Tcor_12393 (91-584)
Trachymyrmex zeteki	Tzet_16480 (1-341)	Tzet_10233 (61-555)
Cyphomyrmex costatus	Ccos_09629 (2-343)	Ccos_12360 (35-528)
**Outgroups:**		
Solenopsis invicta	XP_011172553.1 (22-367)	XP_011171734.1 (62-555)
Pogonomyrmex barbatus	XP_011644658.1 (22-367)	XP_011635262.1 (60-553)
Monomorium pharaonis	XP_012530946.1 (22-367)	XP_012533480.1 (92-585)
Vollenhovia emeryi	XP_011861828.1 (23-368)	XP_011862497.1 (92-585)
Wasmannia auropunctata	XP_011688020.1 (22-367)	XP_011685543.1 (95-588)

Supplementary Table 29**.** List of primer names and sequences used for ddPCR.

**Oligo name**	**Sequence**
Ae635-F1	5'-TGCGGTCAGTTGAATCCTAC-3'
Ae635-R1	5'-GTTGAGATCGCCAGCAGTTA-3'
Ae635-P1	5'-AGCCAGACATCTTCCACATGGGCGGTG-3' (FAM/BHQ)
Ae4959-F1	5'-GGTCATCACGCTGGACTTTA-3'
Ae4959-R1	5'-GCCCTACCATAGAATGGCAC
Ae4959-P1	5'-TCGCCCACGAGCGGAGACAAGCT-3' (FAM/BHQ)
AeRPL18-F1	5'-CGTCATCGTCGGTACAATCA-3'
AeRPL18-R1	5'-GTGATGATCTCGCCTCCATT-3'
AeRPL18-P1	5'-TGCGTGCCCGAGCCTTCTCAGTGA-3' (HEX/BHQ)
AeTBP-F1	5'-AGGTTTGCGGCTGTTATCAT-3'
AeTBP-R1	5'-TTCTTGCGTACTTTCTGGCA-3'
AeTBP-P1	5'-ACGTGAATCTCCCTCACTCTTGGCGCC-3' (HEX/BHQ)

Supplementary Methods

Overview

Our sequencing efforts focused on five species of ants and six fungal cultivars: *Atta colombica* (Acol), *Acromyrmex echinatior* (Aech), *Trachymyrmex septentrionalis* (Tsep), *T. cornetzi* (Tcor), *T. zeteki* (Tzet), and *Cyphomyrmex costatus* (Ccos) (Supplementary Table **1**). The genome of *Ac. echinatior* has previously been published[1] and the same is true for the genomes of *Atta cephalotes* (Acep)[2] and its cultivar[3]. Accession numbers for the data generated in this study are given in Supplementary Table **2**. The ant species name abbreviations given in parentheses will be used in figures and tables throughout this document, with "-F" appended for their associated fungal cultivars.

Biological material

Queenright colonies of *C. costatus*, *T. zeteki* and *T. cornetzi* were collected in Gamboa, Panama and maintained in the lab on a diet of polenta, oatmeal and bramble leaves at 25 °C and 60 % – 70 % RH. For *C. costatus* ca. 1200 gynes, males and workers from 15 colonies (Ccos006, Ccos011, 100624-19, 100604-13, 100617-03, 100603-04, 100629-20, 100610-02, 100625-12, 100611-01, 100610-01, 100618-02, 100624-20, 100611-05, 100611-02) were used for genome sequencing, and males, gynes and brood from colony 100610-02 were used for RNA extraction. For *T. zeteki* ca. 400 gynes, males and workers from a single colony (Tzet028) were used for DNA extraction, and workers, males and brood from the same colony were used for RNA

extraction. For *T. cornetzi* ca. 400 gynes, workers and males from a single colony (Tcor002) were used for DNA extraction, and gynes, males, workers and brood form the same colony were used for RNA extraction. For *T. septentrionalis* ca. 450 males were collected from a single colony (CR110607-02) from Apalachicola National Forest, Tallahassee, Florida, USA, and stored in RNAlater at -80 °C for DNA extraction, and gynes, males, workers and brood form the same colony were used for RNA extraction. For *At. colombica*, a leaf-cutting ant with much larger reproductives, two males from a single colony (Treedump-2) from Gamboa, Panama, were collected and used for DNA extractions and gynes, males, workers and brood from the same colony were used for RNA extractions.

Fungal cultures of the cultivar of *C. costatus* (100610-02), *T. zeteki* (Tzet028), *T. cornetzi* (Tcor002), *Ac. echinatior* (Ae372) and *At. colombica* (Treedump-2) were obtained by plating small tufts of the cultivar on PDYA (Potato Dextrose Yeast-extract Agar) plates containing streptomycin. After having obtained pure cultures, pieces of mycelium were put on PDYA plates covered with sterile cellophane disks and incubated for a few months at 25 °C. Mycelium was collected and used for DNA extraction (only *C. costatus* and *Ac. echinatior*) and RNA extraction. A fungal culture of the cultivar of *T. septentrionalis* was obtained from a colony (SAR040627-01) collected at Appomattox-Buckingham State Forest, Virginia, USA, by plating small tufts of the cultivar on PDA (Potato Dextrose Agar) plates containing streptomycin and penicillin. After having obtained pure cultures, mycelium was propagated in liquid PDA medium, harvested and stored in RNAlater at -80 °C until RNA extraction.

DNA and RNA extraction

DNA was extracted from ants using the QIAGEN Blood and Cell Culture DNA Mini Kit using the protocol enclosed in the kit with modifications. Ant tissues were manually disrupted in G2 lysis buffer using a Teflon pestle. After addition of 1 % proteinase K (20 mg/ml) and 0.2 % RNAse A (QIAGEN, 100 mg/ml), the samples were incubated for 2 – 3 hours at 50 °C on a rotating wheel. One sample volume of chloroform was added after which samples were incubated for 30 – 60 min at 50 °C and centrifuged for 10 min at 5,000 g, transferred to the Genomic-tip, and processed according to the protocol.

DNA was extracted from ca. 2 g fungal mycelium by grinding the mycelium in liquid nitrogen with a mortar and pestle. 400 mg ground mycelium was mixed with 5 ml extraction buffer (2 % CTAB, 1.4 M NaCl, 0.1 M Tris, 20 mM EDTA, 1 % Polyvinylpyrrolidone, pH 8), 50 µl Proteinase K (20 mg/ml), 50 µl RNAse A (100 mg/ml) and 50 µl β-mercaptoethanol and incubated at 65 °C for 3 hours. Samples were centrifuged for 10 minutes at 3000 g, and the supernatant transferred to a clean tube. One sample volume of phenol:chloroform:isoamylalcohol (25:24:1), pH 8 was added, after which the tubes were mixed thoroughly by inversion, incubated at room temperature for 5 minutes, and centrifuged for 30 minutes at 3,000 g. The upper phase was transferred to a clean tube and mixed with one sample volume of chloroform:isoamylalcohol (24:1), mixed by inversion and centrifuged for 10 minutes at 3,000 g. The upper phase was again transferred to a clean tube and mixed with 1/3 sample volume of 5 M NaCl and 2/3 sample volume of isopropanol. After careful mixing by inversion, the tubes were centrifuged for 20 minutes at 3,000 g. The supernatant was discarded and the pellet washed with 70 % ethanol. After a brief centrifugation the supernatant was discarded and the DNA pellet dried at room

temperature. The DNA was dissolved in TE buffer over night at 5 °C. The samples were finally centrifuged for 10 minutes at 15,000 g to pellet any undissolved material, and the supernatant was transferred to a clean tube.

Total RNA was extracted from ant tissue using the QIAGEN RNeasy Mini Kit with modifications. Ant tissue was disrupted in 500 µl RLC buffer (with 1 % β-mercaptoethanol) in a Fastprep machine at level 6 for 30 seconds, with 5 mm ceramic beads. One sample volume of phenol:chloroform:isoamylalcohol (25:24:1), pH 8, was added, and the samples were vortexed and then centrifuged for 30 minutes at 20,000 g. The upper phase was transferred to a clean tube and one sample volume of chloroform:isoamylalcohol (24:1) was added, after which the samples were vortexed and centrifuged for 15 minutes at 20.000 g. The upper phase was transferred to a clean tube and processed according to the protocol enclosed in the kit.

Total RNA was extracted from mycelium cultures using the QIAGEN RNeasy Plant Mini Kit with the supplied RLC lysis buffer. Disruption of the tissue was accomplished by addition of ca. 100 µl 0.5 mm glass beads before subjecting them to bead beating using a Fastprep machine set at level 6 for 30 seconds.

DNA and RNA sequencing

For genome sequencing of the ants, five to six DNA sequencing libraries of different insert sizes were made for each species. Five libraries (200bp, 500bp, 800bp, 2kb, 5kb) were constructed for *T. zeteki*, while an additional 10Kb library was constructed for the four other ants. DNA libraries were also constructed for two fungal symbionts: 200bp and 500bp insert libraries for the cultivar of *Ac. echinatior*, and 200bp, 500bp, 800bp, 2kb, and 5kb insert libraries for the cultivar of *C. costatus*.

For the small insert paired-end libraries of 200, 500 and 800 bp, 5 µg of DNA was shattered into fragments and then end-repaired, A-tailed, and ligated to Illumina paired-end adapters. The ligated fragments were size selected at 200, 500 or 800 bp on agarose gels and amplified by LM-PCR to generate the corresponding short insert libraries. For long insert size mate-pair library construction, 20 – 40 µg of genomic DNA was shattered to the desired insert size using nebulization for 2 kb or HydroShear (Digilab) for 5 and 10 kb. Then, the DNA fragments were end-repaired using biotinylated nucleotide analogs (Illumina), and circularized by intramolecular ligation. Circular DNA molecules were sheared using Adaptive Focused Acoustics (Covaris) to an average size of 500 bp. Biotinylated fragments were purified on magnetic beads (Invitrogen), end-repaired, A-tailed, and ligated to Illumina paired-end adapters, size-selected again, and purified by LM-PCR. The constructed DNA libraries were paired-end sequenced on an Illumina HiSeq 2000 platform with read lengths of 100 bp for small insert sizes and 49 bp for large insert sizes.

We also constructed a RNA sequencing library for each attine ant and their symbiotic cultivars. First-strand cDNA was synthesized with random hexamers and Superscript II reverse transcriptase (Invitrogen). Second strand cDNA was synthesized with *E. coli* DNA PolI (Invitrogen). Double-stranded cDNA was purified with a Qiaquick PCR purification kit (QIAGEN) and sheared with a nebulizer (Invitrogen) to 100 to 500 bp fragments. cDNA

fragments were end repaired, ligated to 39 dA overhang and Illumina PE adapter oligo mix, then size selected to 200 bp fragments by agarose gel. After PCR amplification, the libraries were paired-end sequenced using Illumina HiSeq 2000 with a read length of 90 bp.

<u>Genome assemblies</u>

Before assembly, we performed filtering to exclude low quality raw reads that met any of the following criteria: 1) ≥ 5% Ns or polyA; 2) ≥ 50 low-quality bases (Phred score ≤ 7); 3) adapter contamination present; 4) paired reads overlapping each other with ≥ 10 bp (allowing 10 % mismatch); 5) PCR duplicates (reads are considered duplicates if read1 and read2 of the same paired end reads are identical). Low-quality ends were trimmed directly. For small insert size libraries (200 bp, 500 bp and 800 bp), we also performed an error correction step using the correction tool released with SOAPdenovo [4]. The statistics for raw and cleaned data are given in Supplementary Table **3**. We also used the cleaned data to estimate the genome sizes of the five ants and the fungal cultivars of *C. costatus* and *Ac. echinatior* using k-mer depth distribution analysis[4] (Supplementary Figure **1** and Supplementary Figure **2**). Based on 17-mer analyses, the genome sizes of the five attine ants were estimated to be 280 Mb to 396 Mb (Supplementary Table **4**), and the estimated genome sizes of the fungi were 138 Mb and 107 Mb, respectively (Supplementary Table **5**). The k-mer depth distribution of the *Ac. echinatior* cultivar showed two peaks instead of the single peak of a normal near-Poisson distribution, indicating a relatively high heterozygosity rate in the data, consistent with this fungal cultivar being an allopolyploid[5].

After data preprocessing, we used SOAPdenovo (v2.04)[6] to assemble the genomes. We first constructed contigs based on the short insert libraries (parameters –M 2 –d 1), then joined the contigs to scaffolds using paired-end information from all DNA libraries. Unresolved gap regions were then locally reassembled by GapCloser (released with SOAPdenovo). After assembly, we used BLAST[7] (E-value cutoff: 1e-5) to check for contaminant sequences by blasting the assemblies against the bacterial (for the fungal assembly) or bacterial and fungal (for ant assemblies) NCBI nt databases. If a scaffold aligned with identity greater than 80% and total alignment length longer than 50 % of the scaffold length, we considered it a contaminant sequence and removed it from the assembly after manual confirmation. In the ant assemblies, some bacterial contamination was found (Supplementary Table **6**) but no significant fungal contamination. In the *C. costatus* cultivar assembly, we found no contaminant sequences.

Overall statistics of the obtained assemblies after excluding contaminant sequence are given in Supplementary Table **7**. For the *Ac. echinatior* cultivar genome both contigs and scaffolds were very short, presumably due to the heterozygosity problem specified above. As the obtained assembly was too fragmented for overall analyses, we did not perform repeat or gene annotation for the *Ac. echinatior* cultivar genome.

Compared to the other ant assemblies, *T. cornetzi* has a relatively large genome and its assembly has relatively short scaffolds, due to a higher repeat content, as detailed below. GC content distributions of the assemblies are very similar across the attine ants (Supplementary Figure **3**). Coverage was calculated by mapping the clean short reads back to the assemblies using SOAPaligner[8], which produced the sequencing depth distributions given in Supplementary Figure **4** and Supplementary Figure **5**.

Repeat annotation

Following assembly, repeat content of the ant and fungal cultivar assemblies was annotated using a combination of several programs. First, tandem repeats were identified using Tandem Repeats Finder (v4.04, Parameter: Match=2 Mismatch=7 Delta=7 PM=80 PI=10 Minscore=50 MaxPeriod=2000)[9]. Second, transposable elements were identified by combining both homology-based and *de novo* methods, after identifying known transposable elements in the genome using RepeatMasker (version 3.2.6)[10] by searching against Repbase (v17.06)[11]. Third, for *de novo* predictions, we used the programs LTR_FINDER (v1.0.5, default parameter)[12], PILER (v1.0, default parameter)[13], and RepeatScout (v1.0.5, default parameter)[14] to identify repeats in the assemblies. The results of these *de novo* predictors were combined into a *de novo* repeat library, which was then used by RepeatMasker to identify additional high and medium copy repeats (>10 copies) in the genome assemblies. An overview of the different types of repeats identified by these methods is given in Supplementary Table **8**.

The largest attine ant genome of *T. cornetzi* has ca. 170 Mb of repetitive content, covering 42 % of the genome, while the genomes of the four other newly sequenced ants have 67 Mb to 110 Mb (24-34%) repetitive content. As shown in Supplementary Figure **6** the size of the repetitive content of all five ants is approximately linearly correlated with the genome sizes. Thus the large genome size of *T. cornetzi* can be attributed mainly to a high repeat content. The highest-frequency repeat families of the ant assemblies are listed in Supplementary Table **9**, showing that the expansion of the *T. cornetzi* genome is not merely due to a few dominant repeat families, as many repeat families appear to be expanded. TcMar-Mariner is the overall largest repeat family in the attine ants, and *At. colombica* has considerably more TcMar-Mariner sequence (41.2 Mb) than the other four ant genomes (1.4 - 16.2 Mb).

Annotation of protein-coding genes in ant genomes

To annotate protein-coding genes in the ant assemblies, we performed both *de novo* and homology-based predictions, assembled transcripts based on RNA-seq data, and finally combined the different lines of evidence into a single integrated gene set for each species.

For homology-based gene predictions, protein sequences from 11 animal species (*Harpegnathos saltator, Camponotus floridanus, Ac. echinatior, At. cephalotes, Solenopsis invicta, Linepithema humile, Pogonomyrmex barbatus, Apis mellifera, Drosophila melanogaster, Caenorhabditis elegans, Homo sapiens*) were mapped to the genome using TBLASTN (v.2.2.26, parameter: –e=1e-5)[7]. The alignments were then passed to GeneWise (v2.2.0, default parameters)[15] to generate gene models, which were then filtered as follows: 1) very short genes (CDS length < 90 bp) were removed; 2) Translated protein sequences of the predicted genes were realigned against the homologous proteins, and genes with low alignment quality (aligned rate < 0.50, percent identity < 0.25) were removed; 3) according to the GeneWise output, the processed pseudogenes were removed.

We merged the homology-based gene models predicted by the gene sets of *H. saltator, Ca. floridanus and Ac. echinatior* (all of which have been made using the BGI annotation pipeline)

into a union gene set (named ANT1), choosing the longest gene model for each locus. The gene models predicted by the gene sets of *At. cephalotes, S. invicta, L. humile and P. barbatus* (mainly done using MAKER annotation pipelines) were similarly merged into a union gene set, named ANT2. Overall statistics of homology-based predictions are given in Supplementary Table **10**.

De novo gene prediction was performed on repeat-masked genomes using the programs AUGUSTUS (v2.5.5, default parameters)[16] and SNAP (v 2006-07-28, default parameters)[17]. The appropriate parameters of the obtained gene models were trained with 500 high-quality homology-based predictions based on the gene set of *Ap. mellifera*. After running the predictions, additional filtering steps were performed to remove false positives: 1) very short genes (CDS length <150 bp) were filtered; 2) Genes were removed when predicted to overlap with two or more homology-based genes predicted with the gene sets of *D. melanogaster* or *Ap. mellifera*.

Predictions from AUGUSTUS and SNAP were combined into a single *de novo* set, keeping only gene models supported by both programs and using the gene delimitations predicted by AUGUSTUS. The statistics of *de novo* predictions are shown in Supplementary Table **11**.

RNA-seq data were used to improve annotation. For each ant, we first mapped the RNA-seq reads onto the assembly with TopHat (v1.3.3, parameter: -I 100000 –r 20 --mate-std-dev 10)[18] and then assembled transcripts with Cufflinks (v1.2.0, parameter: -I 100000)[19]. Overall statistics of RNA-seq data are given in Supplementary Table **12**.

For each ant, the evidence derived from homology-based predictions (*H. sapiens, D. melanogaster, C. elegans, Ap. mellifera,* ANT1 and ANT2) and *de novo* predictions (one *de novo* gene set) were integrated to generate a consensus gene set by GLEAN[20]. The GLEAN gene set and the assembled transcripts were then integrated to generate an improved gene set: First, a Markov model was estimated from the 500 training gene sets used in the *de novo* annotation by two awk scripts which are included with Geneid gene annotation tools (v1.3)[21]. Second, for the exon sequences we estimated the transition probability distribution of each nucleotide given the pentanucleotide preceding it for each of the three possible frames, and an initial probability matrix from the pentamers observed at each codon position using the awk script MarkovMatrices.awk. Third, for the intron sequences a single transition matrix was computed as well as a single initial probabilty matrix using the awk script MarkovMatrices-noframe.awk. Fourth, the coding potential of each reading frame in the inferred transcript was computed based on the Markov model. Transcripts with complete ORFs were picked out and the redundant isoforms were removed by keeping the longest ORF for each locus. Then these ORFs were integrated with the GLEAN annotation to replace the incomplete GLEAN gene models.

We also performed several filtering steps to refine the gene sets: 1) Removing transposon-related genes based on functional annotation. 2) Filtering out single-exon genes with length < 400 bp and no functional annotation (see below) and support from either *D. melanogaster* or *Ac. echinatior* homology predictions. 3) Replacing fragmentary gene models with the original ORF based on Cufflinks transcripts when they overlapped with two or more genes in the integrated gene set. 4) Replacing fragmentary gene models with homology-based gene models when they overlapped with two or more genes in the integrated gene set. 5) Removing species-specific

genes (based on gene family clustering, see below) that overlapped for > 80 % of repeats or had no functional annotation or homology support. 6) Adjusting the gene set of *T. cornetzi* that remained much larger than the other gene sets, because it contained many high-copy-number (≥ 20) genes without functional annotation and RNA-seq read support, by discarding these elements as being transposon-related.

The statistics of the final gene sets of the five ants are given in Supplementary Table **13**. Distributions of some general features (CDS length, intron length, etc.) of the final gene sets are shown in Supplementary Figure **7**.

Functional annotation of ant protein-coding genes

Gene functions were predicted based on the best match of the alignments to the SwissProt database[22] using BLASTP (E-value cutoff 1e-5). The motifs and domains of genes were determined by InterProScan 4.8[23] against proteins of all databases in InterPro[24]. Gene Ontology[25] IDs were obtained for each gene from the corresponding InterPro entry. For KEGG annotation[26], all genes were aligned against KEGG proteins using the KAAS server[27], and the pathways in which each gene might be involved were derived from the best matched protein in KEGG. Overall statistics of functional annotation are listed in Supplementary Table **14**.

Annotation of ant non-coding RNAs

Four types of ncRNAs were annotated in our analysis: microRNAs (miRNA), transfer RNAs (tRNA), ribosomal RNAs (rRNA), and small nuclear RNAs (snRNA). tRNAscan-SE[28] and INFERNAL[29] were used to predict the ncRNAs in the genome and the tRNA genes were predicted by tRNAscan-SE with eukaryote parameters. The rRNA fragments were identified by aligning the rRNA template sequences from invertebrates using BLASTN with E-value cut-off 1e-5. The miRNA and snRNA genes were predicted by searching the Rfam database[30] with INFERNAL. To accelerate the speed, a rough filtering (discarding the BLAST hits against the Rfam with E-value > 1) was performed before running INFERNAL. The numbers of predicted genes are given in Supplementary Table **15**.

Fungal assemblies and annotation

The raw Ilumina reads were filtered to exclude low quality reads with the following criteria for raw reads: 1) remove reads with ≥ 10% of Ns; 2) remove reads with ≥ 40 low-quality bases (Phred score <=7); 3) remove reads with adapter contamination. Also, to avoid GC bias during read sequencing, we trimmed the first 10 bp of each read. Overall statistics of raw and cleaned data are given in Supplementary Table **16**.

The *C. costatus* cultivar genome was annotated using similar methods to those used for the ant genomes. Several fungal genomes were used as references for homology-based annotation: *Aspergillus fumigatus*, *Saccharomyces cerevisiae*, *Coprinopsis cinerea*, *Laccaria bicolor*, *Pleurotus ostreatus*, *Schizophyllum commune* and *Agaricus bisporus*. TBLASTN (v.2.2.26, E-value cut-off: 1e-5) and GeneWise (v2.2.0, default parameter) were then used for homolog prediction. *De novo* predictions were done by AUGUSTUS (v2.5.5, default parameter) and

SNAP (v 2006-07-28, default parameters) and transcripts were identified by TopHat (v1.3.3, parameters: -I 100000 –r 20 --mate-std-dev 10) and Cufflinks (v1.2.0, parameters: -I 100000). Then all evidence was combined by GLEAN as described above for the ant genomes.

All clean transcriptome reads were assembled into transcript sequences (Supplementary Table **16** using Trinity[31], followed by ESTscan[32] and ORF-finder[33] to predict open reading frames within the assembled transcripts. Shared gene predictions were identified by aligning the predicted peptide sequences from both methods using BLASTp (E-value cut-off: 1e-5). An open reading frame identified by ORF-finder was considered reliable if it aligned to the ESTscan prediction with 100% identity and an alignment length greater than 30% of the gene length for both predictions. Redundant genes were removed from the gene set. Since some transcripts could be derived from alternative splicing of the same gene, we performed BLASTp all against all alignments and kept the longest transcript if two sequences had 100 % identity and an alignment length > 80 % for both genes, or if one gene was completely contained within another with 100 % identity. The numbers of genes obtained are given in Supplementary Table **17**. Functional annotation of these fungal genes based on either the genome or transcriptome was carried out using the same method as for the ant gene sets.

Attine ant genome segmental duplications

As *T. cornetzi* had a large genome and gene set, we checked whether this was due to segmental duplications (SDs) of the genome. To identify SDs, self-alignment for each ant genome was generated by LASTZ[34] after which alignment blocks with length >1 kb and identity > 80 % were considered to be SDs. Although *T. cornetzi* has more SDs (17.7 Mb, see Supplementary Table 16) than other ants (6-13 Mb), SDs only make up a small portion of the whole genome (4.42 %), suggesting that the large genome of *T. cornetzi* is mainly due to an abundance of relatively short repeat sequences, as described above (see Supplementary Table **18**).

Ant gene family clustering

To gain insight into the evolution of gene families of attine ants, we clustered the genes of the seven attine ants and five other sequenced ant species (*S. invicta*, *P. barbatus*, *Ca. floridanus*, *L. humile* and *H. saltator*) as well as three outgroup insects (*Ap. mellifera, D. melanogaster, Nasonia vitripennis*) into gene families using OrthoMCL v2.0.9[35]. To identify homologous relationships among sequences, they were first aligned using BLASTp with an e-value cutoff of 1e-5 and an alignment length cutoff of 50 % of the gene length. The genes were then clustered using MCL[36] with the inflation parameter (-I) set at 1.5 based on the BLAST results (Supplementary Table **19**). 2795 families were single-copy in all species and were used for phylogenetic inference (see below).

One-to-one ortholog assignment

We used reciprocal best BLAST hits to identify one-to-one orthologs between different ants. First, protein sequences of the seven attine ant species and two outgroup ant species (*S. invicta* and *P. barbatus*) were used to perform all against all BLASTP, with an E-value cut-off of 1e-5. Pairwise bi-directional best hits were considered orthologous pairs. Next we iteratively chose a

reference species and, for each reference gene, we put paired orthologous genes from other species together to create an ortholog group. If an ortholog was absent in a given species, we considered the gene locus missing for that species. Finally, we merged the ortholog groups from each reference and removed redundant groups, while keeping the orthologous groups that are present in all species to generate one-to-one orthologous groups. This resulted in 7443 one-to-one ant ortholog groups (Supplementary Data 2).

We used the same method to identify orthologs groups of the symbiotic fungi. Genes of the cultivars of *At. colombica*, *Ac. echinatior*, *T. septentrionalis*, *T. zeteki*, *T. cornetzi* and *C. costatus*, along with genes from *Sc. commune* and *Ag. bisporus* as outgroups, were used to perform ortholog assignment. Due to the relatively low completeness of transcriptome-based gene set, we used a looser criterion for creating the transcriptome ortholog groups: at most one ortholog is absent in 6 transcriptomes, and at least one ortholog is present in the outgroup. This resulted in 3499 fungal ortholog groups (Supplementary Data 2). Of these, 1075 ortholog groups were present in a single-copy in all species and were used to build the fungal phylogeny.

Ant phylogenies

We first aligned the protein sequences of 2795 single-copy gene families using MUSCLE[37] with default parameters and then converted the protein alignments into CDS alignments. The 2795 loci were concatenated in Geneious v7.0[38], resulting in a data matrix consisting of 1,886,151 amino acid sites and 13 taxa (see Supplementary Figure **8**). The concatenated matrix was analyzed under the parsimony criterion using a heuristic search and 100 random-taxon-addition replicates in PAUP*[39], resulting in a single optimal tree. Using this maximum-parsimony tree as a reference tree (user_tree_topology), and the 2795 loci as the maximum number of possible partitions, a partitioning analysis was conducted in PartitionFinder[40] in which all possible protein models were considered and compared (models = all_protein) under the Bayesian Information Criterion (BIC) using the hcluster search algorithm, resulting in a scheme consisting of 132 partitions. These partitions and models were employed in a maximum-likelihood analysis in RAxML 7.7.7[41] with hybrid MPI/Pthreads parallelization[42,43], resulting in a best tree with the topology in Supplementary Figure **8**, which is identical to the maximum-parsimony topology. The partitions and models were also employed in maximum-likelihood bootstrap analyses in RAxML consisting of 1152 pseudoreplicates under the "-b" (thorough search) bootstrap option, resulting once again in the same topology (Supplementary Figure **8**) with bootstrap frequencies of 1.0 at all nodes.

We inferred divergence dates for the maximum-likelihood tree using the penalized likelihood approach implemented in r8s v.1.7[44]. The bee outgroup *Ap. mellifera* was excluded from the dating analyses. We calibrated two nodes in our tree with fixed ages based on the results from a large-scale diversification analysis of the ant subfamily Myrmicinae that employed a total of 27 fossil calibrations across 251 species[45]. The two calibrated nodes in our tree correspond to (1) the most recent common ancestor (MRCA) of *C. costatus* and its sister group and (2) the MRCA of *P. barbatus* and its sister group. Three separate analyses were conducted, using the mean, 5% minimum credibility interval, and 95% maximum credibility interval from Ward et al. 2015[45], respectively, to calibrate node 1 (26.6 [19.6, 33.8] MYA) and node 2 (95.4 [85.2,106.0] MYA). The resulting mean dated tree is given in Supplementary Figure **9**.

The resulting trees in newick format are:

Ant ML best tree:

(((Camponotus_floridanus:0.121082,(Pogonomyrmex_barbatus:0.097112,(((Trachymyrmex_zeteki:0.034976,(Trachymyrmex_cornetzi:0.025797,((Acromyrmex_echinatior:0.025137,(Atta_colombica:0.01126,Atta_cephalotes:0.014308):0.021758):0.004158,Trachymyrmex_septentrionalis:0.027518):0.004198):0.011349):0.01258,Cyphomyrmex_costatus:0.053898):0.045761,Solenopsis_invicta:0.093097):0.020498):0.040078):0.012843,Linepithema_humile:0.128703):0.042901,Harpegnathos_saltator:0.155939,Apis_mellifera:0.370453);

Ant dating, Mean:

((Linepithema_humile:128.189089,(((((Trachymyrmex_zeteki:22.868618,((((Atta_colombica:7.050499,Atta_cephalotes:7.050499)Atta:9.163937,Acromyrmex_echinatior:16.214436)Acro:1.555210,Trachymyrmex_septentrionalis:17.769646)sept:1.411430,Trachymyrmex_cornetzi:19.181076)corn:3.687543)Trachy:3.731382,Cyphomyrmex_costatus:26.600000)Cypho:48.027071,Solenopsis_invicta:74.627071)Solen:20.772929,Pogonomyrmex_barbatus:95.400000)Pogo:24.171940,Camponotus_floridanus:119.571940)Camp:8.617149)Lin:32.318036,Harpegnathos_saltator:160.507124)root;

Ant dating, Fixed 5%:

((Linepithema_humile:112.555294,(((((Trachymyrmex_zeteki:17.170612,((((Atta_colombica:5.598853,Atta_cephalotes:5.598853)Atta:6.969109,Acromyrmex_echinatior:12.567962)Acro:1.130312,Trachymyrmex_septentrionalis:13.698274)sept:0.988507,Trachymyrmex_cornetzi:14.686781)corn:2.483831)Trachy:2.429388,Cyphomyrmex_costatus:19.600000)Cypho:45.642977,Solenopsis_invicta:65.242977)Solen:19.957023,Pogonomyrmex_barbatus:85.200000)Pogo:20.115777,Camponotus_floridanus:105.315777)Camp:7.239517)Lin:27.455839,Harpegnathos_saltator:140.011133)root;

Ant dating, Fixed 95%:

((Linepithema_humile:144.438225,(((((Trachymyrmex_zeteki:28.573372,((((Atta_colombica:8.441471,Atta_cephalotes:8.441471)Atta:11.296607,Acromyrmex_echinatior:19.738078)Acro:1.981874,Trachymyrmex_septentrionalis:21.719952)sept:1.852024,Trachymyrmex_cornetzi:23.571976)corn:5.001396)Trachy:5.226628,Cyphomyrmex_costatus:33.800000)Cypho:50.484003,Solenopsis_invicta:84.284003)Solen:21.715997,Pogonomyrmex_barbatus:106.000000)Pogo:28.389627,Camponotus_floridanus:134.389627)Camp:10.048598)Lin:37.366406,Harpegnathos_saltator:181.804631)root;

Fungal phylogenies

The 1075 loci were concatenated in Geneious v7.0[38], resulting in a data matrix consisting of 825,686 amino acid sites and 8 taxa. The concatenated matrix was analyzed under the parsimony criterion using an exhaustive search in the program PAUP*[39], resulting in a single optimal tree. Using this maximum-parsimony tree as a reference tree (user_tree_topology), and using the 1075 loci as the maximum number of possible partitions, a partitioning analysis was conducted in PartitionFinder[40] in which all possible protein models were considered and compared (models = all_protein) under the Bayesian Information Criterion (BIC) using the hcluster search algorithm, resulting in a scheme consisting of 19 partitions. These partitions and models were employed in a maximum-likelihood analysis in RAxML7.7.7[41] with hybrid MPI/Pthreads parallelization[42,43], resulting in a best tree with the topology given in Supplementary Figure **10**, which is identical to the maximum-parsimony optimal topology.
The partitions and models were also employed in maximum-likelihood bootstrap analyses in RAxML consisting of 1152 pseudoreplicates under the "-b" (thorough search) bootstrap option, resulting once again in the same topology (Supplementary Figure **10**) with bootstrap frequencies of 1.0 at all nodes. We inferred divergence dates for the maximum-likelihood tree using the penalized likelihood approach implemented in r8s v.1.7[44]. The most distant outgroup taxon *Schizophyllum commune* was used to root the tree, providing estimates for branch lengths descended from this root node, and was excluded from the dating analyses. We applied a fixed age calibration to the node corresponding to the MRCA of the outgroup *Agaricus* and its sister group using the results from a previous study[46], a procedure similar to another diversification date analysis of lepiotaceous attine cultivars[47]. We conducted three separate analyses using different fixed ages for this node. These fixed ages were obtained from previous age estimates for this node from Geml et al. 2004[46]. Thus, we conducted analyses using the mean age (73 MYA), the 5% minimum age (55 MYA), and the 95% maximum age (91 MYA) calibrations. The resulting mean dated tree is given in Supplementary Figure **11**.

The resulting trees in newick format are:

Fungal ML best tree:

(Agaricus_bisporus:0.19387749213730451348,(Cyphomyrmex_costatus:0.12830919179005234
598,((Atta_colombica:0.02563147866076995879,Acromyrmex_echinatior:0.0240979591504183
1535):0.07689102429678842943,((Trachymyrmex_cornetzi:0.05102454243654579863,Trachy
myrmex_septentrionalis:0.05857574740147841741):0.04889348684938878142,Trachymyrmex_
zeteki:0.07576027487389550008):0.04405496847347186579):0.15122026564727880649):0.063
73493349384266871,Schizophyllum_commune:0.61754621855156377475):0.0;

Fungal dating, Mean:

(Agaricus_bisporus:73.000000,(Cyphomyrmex_costatus:57.745716,((Atta_colombica:7.238475,
Acromyrmex_echinatior:7.238475)LeafCutter:22.439805,((Trachymyrmex_cornetzi:12.419644,
Trachymyrmex_septentrionalis:12.419644)Trachy1:9.122808,Trachymyrmex_zeteki:21.542452)
Trachy2:8.135828)higher:28.067437)attine:15.254284)root;

Fungal dating, Fixed 5%:

(Agaricus_bisporus:55.000000,(Cyphomyrmex_costatus:43.526084,((Atta_colombica:5.464517, Acromyrmex_echinatior:5.464517)LeafCutter:16.935270,((Trachymyrmex_cornetzi:9.384427,Trachymyrmex_septentrionalis:9.384427)Trachy1:6.884209,Trachymyrmex_zeteki:16.268636)Trachy2:6.131151)higher:21.126297)attine:11.473916)root;

Fungal dating, Fixed 95%:

(Agaricus_bisporus:91.000000,(Cyphomyrmex_costatus:71.945059,((Atta_colombica:9.000823, Acromyrmex_echinatior:9.000823)LeafCutter:27.913869,((Trachymyrmex_cornetzi:15.426005, Trachymyrmex_septentrionalis:15.426005)Trachy1:11.349773,Trachymyrmex_zeteki:26.775778)Trachy2:10.138914)higher:35.030367)attine:19.054941)root;

Further details on phylogenetic computations

PAUP and r8s analyses were carried out on Apple computers with Intel processors; RAxML and PartitionFinder analyses were carried out on the Smithsonian Hydra supercomputer (Linux-based with AMD processors).

Obtaining natural history data of attine ants and their fungal cultivars

The natural history data included in Figure 1 were obtained as follows:

Maximum colony size: Colony sizes in the field vary depending on habitat and colony age, but can be satisfactorily captured in orders of magnitude of maximal attainable size, as many previous studies have done as well (see Kooij et al., 2015[5] and contained references).

Queen insemination status: In a previous study[48] it was shown that three of the attine ant species included in our study (*C. costatus*, *T. zeteki*, *T. cornetzi*) have exclusively singly mated queens, whereas two of the leaf-cutting ants (*Ac. echinatior*, *At. colombica*) always have multiply inseminated queens. Data for the two remaining species were obtained for the present study. We genotyped ca. 50 workers from six *At. cephalotes* colonies collected in Gamboa, Panama, using four polymorphic microsatellite markers Atco 13, Atco 15, Atco 37, Atco 47[49] and confirmed multiple insemination of queens in five of them. For *T. septentrionalis*, we genotyped ca. 10 workers from 10 field colonies made available by Jon Seal, University of Texas at Tyler, using the polymorphic microsatellite markers Atco 15, Atco 12, Atco 13, Cypho 9-10[49,50]. The results indicated full-sibling relatedness in eight colonies and established that a few deviating genotypes in the two remaining colonies were too different to be half-siblings and must therefore have been drifters from other colonies or indicative of colonies being sometimes headed by more than a single queen. We therefore listed *At. cephalotes* as having multiply inseminated queens and *T. septentrionalis* as having singly inseminated queens.

Worker polymorphism: The ancestral state is that ants have morphologically differentiated queens and a single worker caste[51], a state that has been maintained in all basal branches of the

attine ant phylogeny. For *Ac. echinatior* leaf-cutting ants it has been documented that there are two worker castes: large workers with an approximately normal size distribution, and a small worker caste with a skewed size distribution including a prolonged right tail that has sometimes been referred to as "media"[52]. In *Atta* leaf-cutting ants there is an additional soldier caste and further caste differentiation among the nurses and foragers[51].

Obligate presence of cultivar staphylae: The staphylae (clusters of gongylidia on which higher attine ants feed) were identified as a specific symbiotic organs of *Atta* and *Trachymyrmex* cultivars by[53,54] and their consistent production was later confirmed to be a synapomorphy shared by all cultivars of higher attines and leaf-cutting ants[55]. This inference remains correct today as no higher attine ants cultivating fungi without staphylae have been found. A recent study did, however, show that the lower attine ant *Apterostigma megacephala* secondarily acquired a higher attine symbiont with staphylae[56] and another study showed that the fungal cultivar of one lower attine ant species (i.e., *Mycocepurus smithii*) occasionally produces staphylae, but with significantly smaller gongylidia[57].

Ploidy level of cultivar: Details for how these data have been obtained are provided in Kooij et al. (2015)[5]. Assessment of the degree of polyploidy in the cultivar of *T. septentrionalis* is ongoing.

Determining pairwise synteny

Pairwise genome synteny was determined among attine ants, among five other sequenced ant species (*S. invicta*, *P. barbatus*, *Ca. floridanus*, *L. humile* and *H. saltator*), among 12 fruit flies, eight primates, 22 birds and 16 mosquitoes downloaded from Ensembl database[58] (Supplementary Table **20**).

To identify syntenic blocks, orthologous relationships were first identified using BLASTp searches (e-value cutoff 1e-5) between all species pairs within each phylogenetic group. Reciprocal best hits (RBH) were considered orthologs. Pairwise syntenic blocks were then identified based on coordinates of these orthologs as follows: We required each syntenic block to contain at least five contiguous orthologous genes, and for a block to be extended the gap had to be be smaller than five genes. No more than five gene inversions were allowed in syntenic blocks between two species.

Rates of loss of synteny

The loss of synteny between species pairs was assumed to follow an exponential decay process, and rates of synteny loss were calculated accordingly as $1\text{-}p_s^{1/T}$, where T is divergence time (in millions of years) and p_s the estimated proportion synteny between two species. There was some evidence that rates of synteny loss were higher for species pairs that had diverged very recently (<5 million years ago; see Supplementary Figure **12**). This might be expected for recently-diverged species, where chromosomal rearrangements may evolve rapidly to reinforce genetic isolation of species pairs[59], but it may also result from the way we have defined syntenic blocks (see above), as the choice of number of orthologous genes and gap sizes is expected to have a

greater effect on initial divergence rates. However, there is no reason to suppose that either of these effects would vary between larger taxonomic groups, and inclusion or exclusion of pairs with <5 MY divergence gave similar results (see below). Overall differences between taxonomic groups in their rates of pairwise synteny loss were tested using a Kruskal-Wallis non-parametric test, and pairs of groups were compared using a Steel-Dwass pairwise post-hoc test.

Overall difference in rates of synteny loss between groups were highly significant (Kruskal-Wallis test, $H = 104.8$, d.f. = 5, $P < 0.0001$), and all *post-hoc* pairwise comparisons were also significant ($|Z| > 3$, $P < 0.0001$ to 0.0229) with the exception of those between primates and birds ($Z = -2.08$, $P = 0.295$), mosquitoes and *Drosophila* ($Z = -1.26$, $P = 0.808$) and mosquitoes and non-attine ants ($Z = 2.68$, $P = 0.079$). Excluding species pairs with divergence times <5 MY gave similar results (overall difference: $H = 101.2$, d.f. = 5, $P < 0.0001$), but now the difference between mosquitoes and non-attine ants was also significant ($Z = 3.11$, $P = 0.023$). Calculations were performed in JMP version 11.2.0.

These results confirm earlier findings that amniotes (primates and birds) have reduced rates of chromosomal rearrangement[60], but show more variation between insect groups than previously found[61].

Mapping loss of synteny onto the ant phylogeny

Loss of synteny along the branches of the ant phylogeny was estimated by using the FITCH package in the PHYLIP suite of programs v. 3.695[62], which reconstructs phylogenies based on distance matrices, which are assumed to be additive, but does not make assumptions about an evolutionary clock. The input file was the pairwise loss of synteny between pairs of ant species, which was treated as a distance matrix and mapped onto the ant phylogeny by using the “U” option to specify a user-defined tree with branch lengths, derived from the dated phylogeny based on genome sequences.

Mapping rates of synteny divergence onto the ant phylogeny showed that differences in the rates of synteny loss between attine and non-attine ants were primarily due to high rates of loss of synteny along the terminal branches in the attine clade.

Consistently expanded or contracted gene families

We initially used badirate[65] (with -bmodel FR, -rmodel BDI, -ep ML -out, and using the "mean tree" phylogenetic time estimates as described in 'Phylogenies' above) to estimate the gene birth, death, and innovation rates in the attine ant gene families (see 'Assemblies and annotation' for gene family assignment methods). We used gene family counts from the seven attine genomes and the two outgroups *S. invicta* and *P. barbatus*. However, the resulting rates were inflated and highly correlated with branch lengths (Pearson's R up to 0.97, $P < 0.002$), likely due to short ancestral branches and incomplete lineage sorting. We therefore disregarded the rate estimates themselves and used only the "outlier" gene families that were inferred to evolve at significantly increased rates. Gene models and family assignments for these candidate outlier families were manually checked, resulting in the identification of two significantly expanded gene families: Nardilysin and Tom70, both of which were expanded in all attine ants, see Supplementary Table

21 and Supplementary Figure **13**. Subcellular localization of potentially full length *Ac. echinatior* and *At. colombica* Nardilysin proteins was inferred using the WoLF PSORT web interface (www.genscript.com/psort/wolf_psort.html)[66].

To assess overall trends in gene family expansions and contractions, we counted the number of consistently expanded or contracted gene families at ancestral nodes based on gene family sizes at the terminal nodes. At each ancestral node, we compared gene family sizes of the ingroup (speciation after this node) versus outgroups (speciation before this node). For consistent expansions, we required that the minimum family size of the ingroup be greater than the maximum family size of the outgroups. The estimates therefore include novel gene families with 0-counts in all outgroups. For consistent gene family contractions we conversely required that the maximum family size of the ingroup be smaller than the minimum family size of the outgroups.

Since sampling alone could account for some of the observed differences, we sampled all possible permutations for subsets of two or greater and calculated consistently expanded and contracted families as described above. Based on these observed distributions the 5th and 95th percentiles were calculated and compared to the observed data (Supplementary Figure **14**). Calculations were done in R version 3.0.3[67].

To check whether novel genes (gene families with 0-counts at all ancestral nodes) were derived *de novo* or could originate from other protein-coding sequences, we used BLASTp of the novel genes against the NCBI nr database with a relaxed cutoff of 0.1. Genes with no matches to any metazoan sequence (based on the NCBI Taxonomy classification) were considered likely *de novo* derived. To rule out horizontal gene transfer, these genes were additionally checked with BLASTp (e-value cutoff 0.01) against NCBI nr sequences of plant, fungal, or bacterial origin. No matches were found.

<u>Arginine biosynthesis pathway loss</u>

Two genes encoding the argininosuccinate synthase and argininosuccinate lyase enzymes that are involved in arginine biosynthesis were earlier found lost in the genomes of the evolutionarily derived leaf-cutting ants *Ac. echinatior* and *At. cephalotes*[1,2]. To find out when these two genes were lost during the evolution of the attine ants, we used the intact CDS sequences from *S. invicta* and *P. barbatus* as references to map to the attine assembly by BLAT (v .35x1, default parameters)[63]. This showed that the argininosuccinate synthase gene is completely lost in all attine ants, while the three *Atta* and *Acromyrmex* leaf-cutting ants, *T. septentrionalis* and *T. zeteki* have retained regions similar to the argininosuccinate lyase gene. To clarify whether these regions were pseudogenized, we used Genewise (v2.2.0, default parameter)[15] to predict gene structures from the peptide references of *S. invicta* and *P. barbatus*. This procedure identified several frame shifts and pre-stop codons in these regions, indicating that all these argininosuccinate lyase gene regions were pseudogenized.

To confirm that these gene loss events were not caused by assembly errors, we checked the gene synteny of the flanking regions and found that these were intact (Supplementary Figure **15** and Supplementary Figure **16**). We also aligned the pseudogenized argininosuccinate lyase gene

sequences to the *S. invicta* and *P. barbatus* references to establish which mutations were responsible for the loss of function.

dN/dS ratio estimations

Sequences of one-to-one orthologous groups of seven attine ants and outgroup ants were used to generate multiple codon-based alignments by PRANK v.120716[69] using default parameters. Guidance v1.2[70] was then used for assessing alignment qualities (set "--bootstraps 10" and other default parameters). We considered aligned codons with Guidance site-wise scores of < 0.5 as being low-quality sites and marked them as Ns in the alignments for subsequent PAML [71] analyses.

To investigate changes in dN/dS ratios associated with evolutionary transitions in the attine phylogeny, we used three different models: Model 1 had one dN/dS ratio for the outgroup ants, and another for all the attine ants (for a total of two dN/dS ratios). Model 2 added an extra dN/dS ratio for all higher attines (including leaf-cutting ants, for a total of three ratios), while model 3 additionally had a specific dN/dS ratio for leaf-cutting ants only (for a total of 4 dN/dS ratios). PAML[71] version 4.7 was run twice for each alignment with different start values (Kappa 2.5 or 1, Omega 0.2 or 2) and non-converging alignments, and those yielding dN/dS estimates >3 were removed. This resulted in 6057 ortholog alignments. Likelihoods of model 2 versus model 1 (distinct dN/dS ratio for higher attine ants) and of model 3 versus model 2 (distinct dN/dS ratio for leaf-cutting ants) were then compared with log-ratio tests (LRT). Ortholog alignments where this test generated significant P-values (FDR-corrected P-value < 0.05), and where dN/dS ratios were found to increase, were then used for GO analysis in the Cytoscape[72] v.3.1.0 plugin BinGO[73] v.2.44, using the Hypergeometric test and an FDR-corrected P-value cut-off of 0.05 and the GO annotations of the *At. cephalotes* proteins (Supplementary Table **22** and Supplementary Table **23**).

CAZy annotations

To identify carbohydrate active enzymes in the fungal cultivars and outgroups downloaded from the JGI fungal genome database (*Co. cinerea* v1.0, *Ag. bisporus* v2.0, and *Sc. commune* v2.0), we used the annotated protein sequences to do CAZyme identifications. Putative encoded protein sequences were first compared to the full length sequences of the CAZy database (v2013)[74] using BLASTp. Query sequences that produced an e-value $<10^{-6}$ and aligned over their entire length with a protein in the database with >50% identity were retained and assigned to the same family as the subject sequence. To make sure these pre-identified protein sequencs contained a functional CAZyme domain, they were then subjected in parallel to (i) a BLAST search against a library built with partial sequences corresponding to individual Glycoside Hydrolase (GH), Polysaccharide Lyase (PL), Carbohydrate Esterases (CE), Carbohydrate-Binding Modules (CBM) and Auxillary Activities (AA) modules with e-value < 0.01, and (ii) a HMMer search[75] using hidden Markov models custom built for each CAZy module family. A sequence was considered reliably assigned when it was placed in the same family with the two methods. To ensure comparability of the data, two sets of CAZy counts were obtained for the *C. costatus* cultivar: One based on the full annotated genome (similar to the outgroup fungi), and one based

on the transcriptome data (similar to the other higher attine cultivars). To categorize CAZy families according to substrate, we used previously published classifications[76,77].

Statistical CAZy analyses

Clustering of species was done using the R-package pvclust[78] version 1.3-2, using complete clustering and euclidian distances of normalized CAZy counts (Supplementary Data 1). Statistical significance of *C. costatus* cultivar CAZy transcriptomic counts versus the domesticated cultivar transcriptomic counts of the higher attine ants and leaf-cutting ants were assessed using the binomial probability of observing counts equal to or greater than the *C. costatus* count, assuming the sum of all species' counts to be distributed among species with equal probability and treating the domesticated cultivars as a single group (sum) for the purpose of the test. All tests were performed in R version 3.0.3[67].

Fungal Interpro domain losses

Protein Interpro (IPR)[24] annotations of fungal genes were carried out as described in 'Assemblies and annotation' above. Based on these annotations, domains that were observed in the *C. costatus* cultivar and the *Ag. bisporus* outgroup, but were absent in all domesticated cultivars were inferred to be lost in the higher attine ant cultivars. To ensure that the absence of an IPR domains was not due to annotation artefacts, we used HMMER[75] searches with the potentially lost IPR domain profiles against all transcriptomes as well as the genomic assemblies of the *C. costatus*, *Ac. echinatior*, and *At. cephalotes*[3] cultivars. The genomic and transcriptomic sequences were first converted to six frame peptide sequences before searching with HMMER using an e-value of 1e-2 and requiring the length of the match to be greater than 30% of the domain length. This resulted in 20 reliably lost domains in the higher attine cultivars (Supplementary Table **24**).

For the ligninase domain, we assessed the synteny of surrounding genes using manual BLAST searches against the *Ag. bisporus* (H97 v2.0) and *Leucoagaricus gongylophorus* (Ac12 v1.0) genome sequences available at the JGI MycoCosm portal (genome.jgi.doe.gov/programs/fungi/index.jsf). The complete DNA primase gene and part of the putative membrane permease gene were found on the genomic contig gi|482786973|gb|ANIS01002019.1|. The remaining part of the putative membrane permease gene was found on the contig gi|482786958|gb|ANIS01002032.1|. The two contigs are non-overlapping, but overlapping sequence reads were identified in an independent genome sequence of *L. gongylophorus*[79]. Accession numbers for the relevant genes (ligninase domain containing and surrounding syntenic genes) are provided in Supplementary Table **25**.

Positive selection scans

In order to detect positively selected genes in the ancestor of the leaf-cutting ants, the higher non-leaf-cutting attine ants, and all attine ants, we performed PAML (v4.6)[71] analyses.

A total of 7443 multiple alignments of one-to-one ortholog groups of seven attine ants and two outgroup ants (*S. invicta*, *P. barbatus*) as described for the dN/dS analysis in section 'dN/dS ratio

estimations' were used for the analysis. We applied the branch-site mode of codeml (model = 2, NSsites = 2) for the detection of positively selected genes at the ancestral node of each group. Similar methods were used for 3499 single-copy orthologs of the symbiotic fungi and the two outgroup fungi *Ag. bisporus* and *Sc. commune*.

To detect the positively selected sites in ortholog genes, we set the null model (fix_omega = 1, omega = 1) to represent all sites as neutral and the alternative model (fix_omega = 0, omega = 1.5) to detect whether there are positively selected sites. These contrasting models were then compared using likelihood-ratio tests.

The P-values of the LRT test were then adjusted by the FDR method. The orthologs were considered positively selected if adjusted p-values were smaller than 0.05, and if there was at least one site with a Bayes Empirical Bayes (BEB) probability > 0.95. We detected 35, 84, and 223 genes that were positively selected in the ancestral branch of the leaf-cutting ants, the higher attine ants, and all attine ants, respectively. Similar analyses in the fungi identified 97, 290, 622 and 84 genes that were positively selected in the ancestral lineages of the leaf-cutting ant symbionts, the higher attine ant symbionts, all attine ant symbionts, and the *Trachymyrmex* symbionts, respectively. Initial analyses of the fungi were run using only *Ag. bisporus* as an outgroup for the branch-site tests, but interesting candidate genes were reassessed using both outgroups.

To prevent local optimization of the ML estimates, we ran the PAML estimations with different initial initial kappa values of 1.5, 2 and 3, and initial omega values of 1.2, 1.7 and 2. These results confirmed the earlier analyses. We also manually checked the alignment quality around the positively selected sites to exclude that the significance of the LRT tests were caused by false alignments.

Based on the set of positively selected genes found, we examined those involved in chitin metabolism in more detail. For attine ants, these were chitinases and beta-hexosaminidase. For fungal cultivars, these were the chitin synthases, which were rechecked after adding the other *S. commune* outgroup to arrive at our final assessments. The final lists of positively selected genes in fungus growing ants and symbiotic cultivars are given in Supplementary Data 2, Supplementary Table **26**, and Supplementary Table **27**.

Identifying protein features

To identify signal peptides, protein domains, and check the intactness of catalytic sites of ant proteins, sequences were analyzed using PROSITE[80] v. 20.114 (prosite.expasy.org/prosite.html), SMART[81,82] (http://smart.embl-heidelberg.de), and NCBI CDD[83] (ncbi.nlm.nih.gov/Structure/cdd/cddsrv.cgi). Searches were done May-June 2015. The predicted sequence features were found to be in agreement for all attine ant sequences, except for some cases where signal peptides were missing or misclassified as transmembrane regions due to ambiguous N-terminal start sites. The loss of the chitin-binding Peritrophin-A domain (CBM_14, PF01607) in the attine chitinases was confirmed by NCBI tblastn of the *S. invicta* protein against *At. cephalotes* nucleotide sequences. This confirmed that the GH18 (PF00704) portion of the *S. invicta* protein aligned well to an *At. cephalotes* mRNA (XM_012206397.1, 61% identity).

However, the CBM_14 portion showed only a partial match further downstream in the mRNA, and this alignment contained stop-codons and was located in a different reading frame, consistent with pseudogenization of this part of the protein.

Myrmicine ant orthologs of the attine chitinase and beta-hexosaminidase were identified using NCBI blastp with attine ant or *S.invicta* sequences as queries. This yielded consistent sequence clusters indicating orthologous relationships. Where more than one gene, or more than one isoform, existed for a given species, the one with the most similar length to the attine ants was chosen. The resulting groups of sequence ids for the two protein clusters are given in Supplementary Table **28**.

Protein Average Residue Weights and Isoelectric Points were calculculated using the pepstats program from the EMBOSS package[84], version 6.5.7. To ensure that truncated annotations or domain loss of attine ant chitinases did not bias the comparisons, sequences were aligned using the T-Coffee[85,86] server (tcoffee.vital-it.ch/apps/tcoffee/index.html, Version_11.00.8cbe486) and unaligned N- and C-terminal regions outside the domains were removed before calculations were made. Significance tests were performed using phylogenetic ANOVA as implemented in the R-package phytools[87] version 0.4-45 with 10000 simulations, and using the "mean tree" phylogenetic time estimates as described in 'Phylogenies' above (including seven attine ants and two myrmicine ant outgroups). Data normality and equality of variances were assessed using the shapiro.test and var.test functions of R[67] 3.0.3 (2014-03-06).

Proteins are generally least soluble at a pH that equals their isoelectric point[88] so the observed increase in pI can be interpreted as possible adaptations to maintain charge and solubility in an environment of increased pH, as found in the foreguts of leaf-cutting ants[89].

For the fungal proteins, chitin synthase domain annotations and active sites were checked as above. Alignment quality and completeness varied, and positively selected sites were mostly outside known domains. Suitable templates for structural modelling were not available, precluding further functional inferences from the amino acid changes.

Protein structure modeling

Protein modeling was done using SwissModel[90–92] (swissmodel.expasy.org) in both automated and alignment mode. Several modeling templates were tried, and the best ones retained: 3w4r.1.A for the chitinase (QMEAN4 -2.93), 3ozo.1.A for the beta-hexosaminidase (QMEAN4 -4.09). The latter template is a homodimer. Though none of the models produced high-scores, the overall folding remained consistent and poorly scoring regions were primarily confined to non-conserved loop regions that did not contain any of the positively selected sites. Structures were visualized using jalview 2.8.2[93] (Supplementary Figure **17** and Supplementary Figure **18**).

Expression validation - Biological material

Queenright colonies of *Ac. echinatior* were collected in Gamboa, Panama and maintained in the lab on a diet of rice and bramble leaves at 25 °C and 60 % – 70 % RH. The following colonies were used in the experiment: Ae150, Ae322, Ae356 and Ae372. Large workers were submerged

in liquid nitrogen and divided into head (prosoma), mesosoma (thorax and propodeum) and metasoma (gaster and petiole). Five animals were pooled per sample. Labial glands were collected by submerging live large workers into ice cold, sterile, phosphate buffered saline (PBS). After removing the heads, the mesosoma was opened by pulling the front legs and laterocervical plates with forceps, whereafter the two paired labial glands could be collected with forceps and immediately placed in an Eppendorf tube on dry ice. The remaining mesosoma minus labial glands (fragments of the delicate gland tissue might have remained after dissection) was also collected on dry ice. Validations were based on pooled samples of 20 ants each.

Expression validation - RNA extraction and reverse transcription

Total RNA was extracted from ant tissues using the QIAGEN RNeasy Mini Kit with slight modifications. Ant tissue was disrupted in 500 µl RLT buffer (with 1 % β-mercaptoethanol) in a Fastprep machine at level 4 for 45 seconds, with a ¼ inch ceramic bead. After a brief centrifugation to remove foam, samples were transferred to a QIAshredder column and centrifuged for 3 minutes at 20.000 g. Samples were then mixed with exactly one sample volume of 55 % ethanol, transferred to an RNeasy column and processed according to the manual. RNA concentration, integrity and purity were determined using a Nanodrop spectrophotometer (Thermo Scientific) and an Experion automated electrophoresis system (Bio-Rad). Total RNA was reverse transcribed into cDNA using the iScript cDNA Synthesis Kit (Bio-Rad), after which the cDNA was diluted with water to a final concentration corresponding to 5 ng/µl of total RNA.

Expression validation - Droplet digital PCR

Gene expression levels were determined with a QX200 ddPCR system (Bio-Rad) using TaqMan probes. The two genes encoding Ribosomal Protein L18 (RPL18) and TATA-Binding Protein (TBP), with the Genbank accession numbers XM_011064584 and XM_011062766, respectively, were used as housekeeping genes to normalize the expression levels across samples. Primers and probes were designed using the Primer3Plus[94] and PCR efficiency Calculator[95] web interfaces. Primer and probe sequences are presented in Supplementary Table **29**. PCR reactions were run on a Bio-Rad S1000 Thermal Cycler using the ddPCR Supermix for Probes (Bio-Rad) 1 µl of template per reaction (although lower amounts had to be used in some cases to obtain a proper ratio between positive and negative droplets) and a final concentration of primers and probes of 0.9 µM and 0.25 µM, respectively. Each reaction contained primers and probes for one target gene and one housekeeping gene, so the different fluorophores of the probes allowed discrimination between the PCR products. The PCR program was as follows: 95 °C for 10 minutes, 40 cycles of 94 °C for 30 seconds, and 61 °C for 60 seconds, followed by 98 °C for 10 minutes. All steps were performed with a ramp rate of 2 °C per second. Following PCR, the samples were transferred to the ddPCR droplet reader to measure the number of positive and negative droplets. Initial data analysis was performed using the QuantaLife software program.

Expression validation - Data analysis

The absolute transcript concentration of each target gene originating from the QuantaLife software was normalized through division by the geometric mean of the housekeeping gene transcript concentration of the same sample. A pseudocount of 0.08 (corresponding to one

positive droplet in a reaction) was added to all values before taking the base 10 logarithm to stabilize the variances. Differences in mean expression levels of each of the two target genes among the different tissues were investigated using a one-way ANOVA test followed by a *post hoc* Tukey HSD test, using a significance level of 0.05 (n = 4). Expression levels were not significantly different between mesasoma with or without labial glands, which may indicate that other tissues also express these genes.

Supplementary References

1. Nygaard, S. *et al.* The genome of the leaf-cutting ant *Acromyrmex echinatior* suggests key adaptations to advanced social life and fungus farming. *Genome Res.* **21,** 1339–1348 (2011).
2. Suen, G. *et al.* The genome sequence of the leaf-cutter ant *Atta cephalotes* reveals insights into its obligate symbiotic lifestyle. *PLoS Genet.* **7,** e1002007 (2011).
3. Aylward, F. O. *et al. Leucoagaricus gongylophorus* produces diverse enzymes for the degradation of recalcitrant plant polymers in leaf-cutter ant fungus gardens. *Appl. Environ. Microbiol.* **79,** 3770–3778 (2013).
4. Li, R. *et al.* The sequence and de novo assembly of the giant panda genome. *Nature* **463,** 311–317 (2010).
5. Kooij, P. W., Aanen, D. K., Schiøtt, M. & Boomsma, J. J. Evolutionarily advanced ant farmers rear polyploid fungal crops. *J. Evol. Biol.* **28,** 1911–1924 (2015).
6. Luo, R. *et al.* SOAPdenovo2: an empirically improved memory-efficient short-read de novo assembler. *Gigascience* **1,** 18 (2012).
7. Altschul, S. F., Gish, W., Miller, W., Myers, E. W. & Lipman, D. J. Basic Local Alignment Search Tool. *J. Mol. Biol.* **215,** 403–410 (1990).
8. Li, R. *et al.* SOAP2: An improved ultrafast tool for short read alignment. *Bioinformatics* **25,** 1966–1967 (2009).
9. Benson, G. Tandem repeats finder: a program to analyze DNA sequences. *Nucleic Acids Res.* **27,** 573–580 (1999).
10. Smit, A. RepeatMasker Open-3.0. http://www.repeatmasker.org (2010).
11. Jurka, J. *et al.* Repbase Update, a database of eukaryotic repetitive elements. *Cytogenet. Genome Res.* **110,** 462–467 (2005).
12. Xu, Z. & Wang, H. LTR-FINDER: an efficient tool for the prediction of full-length LTR retrotransposons. *Nucleic Acids Res.* **35,** 265–268 (2007).
13. Edgar, R. C. & Myers, E. W. PILER: identification and classification of genomic repeats. *Bioinformatics* **21,** 152–158 (2005).
14. Price, A. L., Jones, N. C. & Pevzner, P. A. De novo identification of repeat families in large genomes. *Bioinformatics* **21,** i351–i358 (2005).
15. Birney, E., Clamp, M. & Durbin, R. GeneWise and Genomewise. *Genome Res.* **14,** 988–995 (2004).
16. Stanke, M. & Waack, S. Gene prediction with a hidden Markov model and a new intron submodel. *Bioinformatics* **19,** ii215–ii225 (2003).
17. Korf, I. Gene finding in novel genomes. *BMC Bioinformatics* **5,** 59 (2004).
18. Trapnell, C., Pachter, L. & Salzberg, S. L. TopHat: discovering splice junctions with RNA-Seq. *Bioinformatics* **25,** 1105–11 (2009).
19. Trapnell, C. *et al.* Transcript assembly and quantification by RNA-Seq reveals unannotated transcripts and isoform switching during cell differentiation. *Nat. Biotechnol.*

28, 511–515 (2010).

20. Elsik, C. G. *et al.* Creating a honey bee consensus gene set. *Genome Biol.* **8,** R13 (2007).
21. Parra, G., Blanco, E. & Guigo, R. GeneID in Drosophila. *Genome Res.* **10,** 511–515 (2000).
22. Boeckmann, B. The SWISS-PROT protein knowledgebase and its supplement TrEMBL in 2003. *Nucleic Acids Res.* **31,** 365–370 (2003).
23. Zdobnov, E. M. & Apweiler, R. InterProScan – an integration platform for the signature-recognition methods in InterPro. *Bioinformatics* **17,** 847–848 (2001).
24. Mulder, N. J. *et al.* InterPro: an integrated documentation resource for protein families, domains and functional sites. *Brief. Bioinform.* **3,** 225–235 (2002).
25. Harris, M. A. *et al.* The Gene Ontology (GO) database and informatics resource. *Nucleic Acids Res.* **32,** D258–D261 (2004).
26. Kanehisa, M. & Goto, S. KEGG: Kyoto Encyclopaedia of Genes and Genomes. *Nucl. Acids Res.* **28,** 27–30 (2000).
27. Moriya, Y., Itoh, M., Okuda, S., Yoshizawa, A. C. & Kanehisa, M. KAAS: an automatic genome annotation and pathway reconstruction server. *Nucleic Acids Res.* **35,** W182–W185 (2007).
28. Lowe, T. M. & Eddy, S. R. tRNAscan-SE: a program for improved detection of transfer RNA genes in genomic sequence. *Nucleic Acids Res.* **25,** 955–964 (1997).
29. Nawrocki, E. P., Kolbe, D. L. & Eddy, S. R. Infernal 1.0: inference of RNA alignments. *Bioinformatics* **25,** 1335–1337 (2009).
30. Griffiths-Jones, S., Bateman, A., Marshall, M., Khanna, A. & Eddy, S. R. Rfam: An RNA family database. *Nucleic Acids Res.* **31,** 439–441 (2003).
31. Grabherr, M. G. *et al.* Full-length transcriptome assembly from RNA-Seq data without a reference genome. *Nat. Biotechnol.* **29,** 644–652 (2011).
32. Iseli, C., Jongeneel, C. V & Bucher, P. ESTScan: a program for detecting, evaluating, and reconstructing potential coding regions in EST sequences. *Proc. Int. Conf. Intell. Syst. Mol. Biol.* 138–148 (1999).
33. Rombel, I. T., Sykes, K. F., Rayner, S. & Johnston, S. A. ORF-FINDER: A vector for high-throughput gene identification. *Gene* **282,** 33–41 (2002).
34. Harris, R. Improved pairwise alignment of genomic DNA. *Penn. State Univ.* (2007).
35. Li, L., Stoeckert, C. J. & Roos, D. S. OrthoMCL: identification of ortholog groups for eukaryotic genomes. *Genome Res.* **13,** 2178–2189 (2003).
36. Enright, A. J., Van Dongen, S. & Ouzounis, C. A. An efficient algorithm for large-scale detection of protein families. *Nucleic Acids Res.* **30,** 1575–1584 (2002).
37. Edgar, R. C. MUSCLE: multiple sequence alignment with high accuracy and high throughput. *Nucleic Acids Res.* **32,** 1792–1797 (2004).
38. Kearse, M. *et al.* Geneious Basic: an integrated and extendable desktop software platform for the organization and analysis of sequence data. *Bioinformatics* **28,** 1647–1649 (2012).
39. Swofford, D. L. PAUP*: Phylogenetic Analysis Using Parsimony (*and Other Methods). http://paup.csit.fsu.edu/ (2002).
40. Lanfear, R., Calcott, B., Ho, S. Y. W. & Guindon, S. PartitionFinder: combined selection of partitioning schemes and substitution models for phylogenetic analyses. *Mol. Biol. Evol.* **29,** 1695–1701 (2012).
41. Stamatakis, A. RAxML version 8: a tool for phylogenetic analysis and post-analysis of large phylogenies. *Bioinformatics* **30,** 1312–1313 (2014).

42. Ott, M., Zola, J., Stamatakis, A. & Aluru, S. Large-scale maximum likelihood-based phylogenetic analysis on the IBM BlueGene/L. *Proc. ACM/IEEE Supercomput. Conf.* **4,** doi:10.1145/1362622.1362628 (2007).

43. Pfeiffer, W. & Stamatakis, A. Hybrid MPI/Pthreads parallelization of the RAxML phylogenetics code. *Parallel Distrib. Process. Work. Phd Forum (IPDPSW), 2010 IEEE Int. Symp.* 1–8, (2010).

44. Sanderson, M. J. r8s: inferring absolute rates of molecular evolution and divergence times in the absence of a molecular clock. *Bioinformatics* **19,** 301–302 (2003).

45. Ward, P. S., Brady, S. G., Fisher, B. L. & Schultz, T. R. The evolution of myrmicine ants: Phylogeny and biogeography of a hyperdiverse ant clade (Hymenoptera: Formicidae). *Syst. Entomol.* **40,** 61–81 (2015).

46. Geml, J., Geiser, D. M. & Royse, D. J. Molecular evolution of *Agaricus* species based on ITS and LSU rDNA sequences. *Mycol. Prog.* **3,** 157–176 (2004).

47. Mikheyev, A. S., Mueller, U. G. & Abbot, P. Comparative dating of attine ant and lepiotaceous cultivar phylogenies reveals coevolutionary synchrony and discord. *Am. Nat.* **175,** E126–E133 (2010).

48. Villesen, P., Murakami, T., Schultz, T. R. & Boomsma, J. J. Identifying the transition between single and multiple mating of queens in fungus-growing ants. *Proc. Biol. Sci.* **269,** 1541–1548 (2002).

49. Helmkampf, M., Gadau, J. & Feldhaar, H. Population- and sociogenetic structure of the leaf-cutter ant *Atta colombica* (Formicidae, Myrmicinae). *Insectes Soc.* **55,** 434–442 (2008).

50. Villesen, P., Gertsch, P. J. & Boomsma, J. J. Microsatellite primers for fungus-growing ants. *Mol. Ecol. Notes* **2,** 320–322 (2002).

51. Hölldobler, B. & Wilson, E. O. *The Ants*. (Harvard University Press, 1990).

52. Hughes, W. O. H., Sumner, S., Van Borm, S. & Boomsma, J. J. Worker caste polymorphism has a genetic basis in *Acromyrmex* leaf-cutting ants. *Proc. Natl. Acad. Sci. U. S. A.* **100,** 9394–9397 (2003).

53. Weber, N. A. Fungus-growing ants. *Science* **153,** 587–604 (1966).

54. Weber, N. A. *Gardening Ants: The Attines*. (American Philosophical Society, 1972).

55. Chapela, I. H., Rehner, S. A., Schultz, T. R. & Mueller, U. G. Evolutionary History of the Symbiosis Between Fungus-Growing Ants and Their Fungi. *Science* **266,** 1691–1694 (1994).

56. Schultz, T. R. *et al.* The most relictual fungus-farming ant species cultivates the most recently evolved and highly domesticated fungal symbiont species. *Am. Nat.* **185,** 693–703 (2015).

57. Masiulionis, V. E. *et al.* A Brazilian population of the asexual fungus-growing ant *Mycocepurus smithii* (formicidae, myrmicinae, attini) cultivates fungal symbionts with gongylidia-like structures. *PLoS One* **9,** e103800 (2014).

58. Flicek, P. *et al.* Ensembl 2014. *Nucleic Acids Res.* **42,** 749–755 (2014).

59. Ayala, F. J. & Coluzzi, M. Chromosome speciation: humans, *Drosophila*, and mosquitoes. *Proc. Natl. Acad. Sci. U. S. A.* **102 Suppl,** 6535–6542 (2005).

60. Larkin, D. M. *et al.* Breakpoint regions and homologous synteny blocks in chromosomes have different evolutionary histories. *Genome Res.* **19,** 770–777 (2009).

61. Simola, D. F. *et al.* Social insect genomes exhibit dramatic evolution in gene composition and regulation while preserving regulatory features linked to sociality. *Genome Res.* **23,**

1235–1247 (2013).

62. Felsenstein, J. Phylip: phylogeny inference package (version 3.2). *Cladistics* **5,** 164–166 (1989).

63. Kent, W. J. BLAT – The BLAST-Like Alignment Tool. *Genome Res.* **12,** 656–664 (2002).

64. Zhang, G. *et al.* Comparative genomics reveals insights into avian genome evolution and adaptation. *Science.* **346,** 1311–1320 (2014).

65. Librado, P., Vieira, F. G. & Rozas, J. BadiRate: estimating family turnover rates by likelihood-based methods. *Bioinformatics* **28,** 279–81 (2012).

66. Horton, P. *et al.* WoLF PSORT: Protein localization predictor. *Nucleic Acids Res.* **35,** W585–W587 (2007).

67. R Core Team. R: A Language and Environment for Statistical Computing. www.R-project.org (2014).

68. Katoh, K. & Toh, H. Recent developments in the MAFFT multiple sequence alignment program. *Brief. Bioinform.* **9,** 286–298 (2008).

69. Loytynoja, A. Phylogeny-aware alignment with PRANK. in *Multiple Sequence Alignment Methods* (Russel, D. J.) 155–170 (Springer, 2014).

70. Landan, G. & Graur, D. Local reliability measures from sets of co-optimal multiple sequence alignments. *Pacific Symp. Biocomput.* **13,** 15–24 (2008).

71. Yang, Z. PAML 4: phylogenetic analysis by maximum likelihood. *Mol. Biol. Evol.* **24,** 1586–1591 (2007).

72. Shannon, P. *et al.* Cytoscape: a software environment for integrated models of biomolecular interaction networks. *Genome Res.* **13,** 2498–2504 (2003).

73. Maere, S., Heymans, K. & Kuiper, M. BiNGO: a Cytoscape plugin to assess overrepresentation of gene ontology categories in biological networks. *Bioinformatics* **21,** 3448–3449 (2005).

74. Lombard, V., Golaconda Ramulu, H., Drula, E., Coutinho, P. M. & Henrissat, B. The carbohydrate-active enzymes database (CAZy) in 2013. *Nucleic Acids Res.* **42,** 490–495 (2014).

75. Eddy, S. R. Accelerated profile HMM searches. *PLoS Comput. Biol.* **7,** e1002195 (2011).

76. Zhao, Z., Liu, H., Wang, C. & Xu, J. Correction: comparative analysis of fungal genomes reveals different plant cell wall degrading capacity in fungi. *BMC Genomics* **15,** 6 (2014).

77. van den Brink, J. & de Vries, R. P. Fungal enzyme sets for plant polysaccharide degradation. *Appl. Microbiol. Biotechnol.* **91,** 1477–92 (2011).

78. Suzuki, R. & Shimodaira, H. Pvclust: an R package for assessing the uncertainty in hierarchical clustering. *Bioinformatics* **22,** 1540–1542 (2006).

79. De Fine Licht, H. H. *et al.* Laccase detoxification mediates the nutritional alliance between leaf-cutting ants and fungus-garden symbionts. *Proc. Natl. Acad. Sci. U. S. A.* **110,** 583–587 (2013).

80. Sigrist, C. J. A. *et al.* PROSITE, a protein domain database for functional characterization and annotation. *Nucleic Acids Res.* **38,** D161–D166 (2010).

81. Schultz, J., Milpetz, F., Bork, P. & Ponting, C. P. SMART, a simple modular architecture research tool: identification of signaling domains. *Proc. Natl. Acad. Sci. U. S. A.* **95,** 5857–5864 (1998).

82. Letunic, I., Doerks, T. & Bork, P. SMART: recent updates, new developments and status in 2015. *Nucleic Acids Res.* **43,** D257–D260 (2014).

83. Marchler-Bauer, A. *et al.* CDD: NCBI's conserved domain database. *Nucleic Acids Res.* **43,** D222–D226 (2015).
84. Rice, P., Longden, I. & Bleasby, A. EMBOSS: the European Molecular Biology Open Software Suite. *Trends Genet.* **16,** 276–277 (2000).
85. Di Tommaso, P. *et al.* T-Coffee: a web server for the multiple sequence alignment of protein and RNA sequences using structural information and homology extension. *Nucleic Acids Res.* **39,** W13–W17 (2011).
86. Notredame, C., Higgins, D. G. & Heringa, J. T-Coffee: a novel method for fast and accurate multiple sequence alignment. *J. Mol. Biol.* **302,** 205–217 (2000).
87. Revell, L. J. Phytools: an R package for phylogenetic comparative biology (and other things). *Methods Ecol. Evol.* **3,** 217–223 (2012).
88. Schmittschmitt, J. P. & Scholtz, J. M. The role of protein stability, solubility, and net charge in amyloid fibril formation. *Protein Sci.* **12,** 2374–2378 (2003).
89. Erthal, M., Peres Silva, C. & Samuels, R. I. Digestive enzymes of leaf-cutting ants, *Acromyrmex subterraneus* (Hymenoptera: Formicidae: Attini): Distribution in the gut of adult workers and partial characterization. *J. Insect Physiol.* **50,** 881–891 (2004).
90. Biasini, M. *et al.* SWISS-MODEL: Modelling protein tertiary and quaternary structure using evolutionary information. *Nucleic Acids Res.* **42,** W252–W258 (2014).
91. Arnold, K., Bordoli, L., Kopp, J. & Schwede, T. The SWISS-MODEL workspace: a web-based environment for protein structure homology modelling. *Bioinformatics* **22,** 195–201 (2006).
92. Kiefer, F., Arnold, K., Künzli, M., Bordoli, L. & Schwede, T. The SWISS-MODEL Repository and associated resources. *Nucleic Acids Res.* **37,** D387–D392 (2009).
93. Waterhouse, A. M., Procter, J. B., Martin, D. M. a, Clamp, M. & Barton, G. J. Jalview Version 2 –A multiple sequence alignment editor and analysis workbench. *Bioinformatics* **25,** 1189–1191 (2009).
94. Untergasser, A. *et al.* Primer3 –new capabilities and interfaces. *Nucleic Acids Res.* **40,** 1–12 (2012).
95. Mallona, I., Weiss, J. & Marcos, E.-C. pcrEfficiency: a Web tool for PCR amplification efficiency prediction. *BMC Bioinformatics* **12,** 404 (2011).
